# Update of the international HerniaSurge guidelines for groin hernia management

**DOI:** 10.1093/bjsopen/zrad080

**Published:** 2023-10-20

**Authors:** Cesare Stabilini, Nadine van Veenendaal, Eske Aasvang, Ferdinando Agresta, Theo Aufenacker, Frederik Berrevoet, Ine Burgmans, David Chen, Andrew de Beaux, Barbora East, Jose Garcia-Alamino, Nadia Henriksen, Ferdinand Köckerling, Jan Kukleta, Maarten Loos, Manuel Lopez-Cano, Ralph Lorenz, Marc Miserez, Agneta Montgomery, Salvador Morales-Conde, Chris Oppong, Maciej Pawlak, Mauro Podda, Wolfgang Reinpold, David Sanders, Alberto Sartori, Hanh Minh Tran, Mireia Verdaguer, Reiko Wiessner, Michael Yeboah, Willem Zwaans, Maarten Simons

**Affiliations:** Department of Surgery (DISC), University of Genoa, Genoa, Italy; Department of Anaesthesiology, University of Groningen, University Medical Centre Groningen, Groningen, The Netherlands; Department of Anaesthesiology, The Centre for Cancer and Organ Diseases, Copenhagen University Hospital Rigshospitalet, Copenhagen, Denmark; Department of Clinical Medicine, University of Copenhagen, Copenhagen, Denmark; Department of Surgery, Vittorio Veneto General Hospital, Vittorio Veneto, Italy; Department of Surgery, Rijnstate Hospital, Arnhem, The Netherlands; Department of Surgery, University Hospital Ghent, Ghent, Belgium; Department of Surgery, University Medical Centre Utrecht, Utrecht, The Netherlands; David Geffen School of Medicine at UCLA, Los Angeles, California, USA; Department of Surgery, Royal Infirmary of Edinburgh, Edinburgh, UK; Department of Surgery, Fakultní Nemocnice v Motole, Prague, Czech Republic; Department of Surgery, Universitat Ramon Llull, Barcelona, Spain; Department of Gastrointestinal and Hepatic Diseases, Copenhagen University Hospital–Herlev and Gentofte, Herlev, Denmark; Vivantes Hospital Berlin, Academic Teaching Hospital of Charité University Medicine, Berlin, Germany; Department of Surgery, Klinik Im Park, Zurich, Zurich, Switzerland; SolviMáx Centre of Excellence for Abdominal Wall and Groin Pain, Eindhoven, The Netherlands; Department of General Surgery, Máxima Medical Center, Veldhoven, The Netherlands; Department of Surgery, Hospital Universitari Vall d’Hebron, Barcelona, Spain; Department of Surgery, Hernia Center 3+CHIRURGEN, Berlin, Germany; Department of Surgery, KU Leuven–University Hospital Leuven, Leuven, Belgium; Department of Surgery, Skåne University Hospital, Malmö, Sweden; Department of Surgery, Universidad de Sevilla, Sevilla, Spain; Department of Surgery, Derriford Hospital Plymouth, Plymouth, UK; North Devon Comprehensive Hernia Centre, North Devon District Hospital, Royal Devon University Healthcare NHS Foundation Trust, Barnstaple, UK; Department of Surgery, Azienda Ospedaliero Universitaria di Cagliari, Cagliari, Italy; Department of Surgery, Gross-Sand Hospital Hamburg, Hamburg, Germany; North Devon Comprehensive Hernia Centre, North Devon District Hospital, Royal Devon University Healthcare NHS Foundation Trust, Barnstaple, UK; Department of Surgery, Ospedale Civile di Montebelluna, Montebelluna, Italy; Westmead Clinical School, Sydney Medical School, University of Sydney, New Galles, Australia; Department of Surgery, Hospital Universitari Vall d’Hebron, Barcelona, Spain; Department of Surgery, Bodden-Kliniken Ribnitz-Damgarten GmbH, Ribnitz-Damgarten, Germany; Department of Surgery, School of Medical Sciences, Kwame Nkrumah University of Science and Technology, P.M.B., Kumasi, West Africa; SolviMáx Centre of Excellence for Abdominal Wall and Groin Pain, Eindhoven, The Netherlands; Department of General Surgery, Máxima Medical Center, Veldhoven, The Netherlands; Department of Surgery, Onze Lieve Vrouwe Gasthuis Hospital, Amsterdam, The Netherlands

## Abstract

**Background:**

Groin hernia repair is one of the most common operations performed globally, with more than 20 million procedures per year. The last guidelines on groin hernia management were published in 2018 by the HerniaSurge Group. The aim of this project was to assess new evidence and update the guidelines. The guideline is intended for general and abdominal wall surgeons treating adult patients with groin hernias.

**Method:**

A working group of 30 international groin hernia experts and all involved stakeholders was formed and examined all new literature on groin hernia management, available until April 2022. Articles were screened for eligibility and assessed according to GRADE methodologies. New evidence was included, and chapters were rewritten. Statements and recommendations were updated or newly formulated as necessary.

**Results:**

Ten chapters of the original HerniaSurge inguinal hernia guidelines were updated. In total, 39 new statements and 32 recommendations were formulated (16 strong recommendations). A modified Delphi method was used to reach consensus on all statements and recommendations among the groin hernia experts and at the European Hernia Society meeting in Manchester on October 21, 2022.

**Conclusion:**

The HerniaSurge Collaboration has updated the international guidelines for groin hernia management. The updated guidelines provide an overview of the best available evidence on groin hernia management and include evidence-based statements and recommendations for daily practice. Future guideline development will change according to emerging guideline methodology.

## Introduction

The European Hernia Society (EHS) has published eight clinical guidelines on all hernia types except diaphragmatic hernias since 2009. The largest project was the (HerniaSurge) International Guidelines for Groin Hernia Management^1^. Fifty expert hernia surgeons, representing all six international hernia societies and the European Association for Endoscopic Surgeons (EAES), published these evidence-based guidelines, including 128 statements and 88 recommendations. Consensus voting sessions were held at international meetings of the EHS, EAES, American Hernia Society and Asia Pacific Hernia Society^2^. The HerniaSurge guidelines were published in 2018, with the literature deadline being January 2015.

Despite a high number of citations that have made HerniaSurge one of the most cited papers in hernia literature, adoption in everyday practice has been limited. Surveys published on the uptake of the recommendations have shown wide variability in the choice of treatment, despite clear guidance in favour of one intervention over another. A recently published paper reported a rate of adoption of laparoscopy below 42 per cent to treat patients with an appropriate indication^3^. Ehlers *et al.*^4^ published how female sex is a risk factor for not receiving a treatment consistent with guidelines and being unhappy with results when undergoing surgery for inguinal hernia.

The same group^5^ tried to explore possible determinants of deviations from recommendations through semi-structured qualitative interviews and realized that factors such as personal beliefs and autonomy of the surgeon and access to resources (availability of devices) are the most relevant influencing factors in the choice of treatment. These observations have highlighted the issues surrounding the publication of evidence-based guidelines that may not be able to be implemented due to barriers and local factors.

The guideline expiry date was June 2018. In June 2020, the HerniaSurge committee members decided to update key chapters where recent publications could alter the statements and/or recommendations published in the ‘expired’ guidelines.

The aim of the present document is to provide updated statements and recommendations pertaining to specific key questions (KQs) from the previous version of HerniaSurge where new evidence is available. Secondary aims include improving patient outcomes, specifically to decrease recurrence rates and reduce chronic pain, the most frequent problems following groin hernia repair.

## Methodology

In 2020 the steering committee of the HerniaSurge collaboration formed a working group (WG) of hernia experts to update the groin hernia guidelines. At the start of the update process, formal tools to help prioritizing key questions were not available^6^. The project was developed from EHS executive board meetings, proposals from the advisory board of quality and on the basis of transparent criteria. These criteria included the time elapsed from the last search in the first publication, availability of new evidence and relevance of the topics. Usually, guidelines are updated in a period ranging from two to five years from their last search^7^; at the time of decision, it was five years since publication, making a new update a priority. The secretary of quality monitors the literature, keeping track of all the new published papers. A working group of senior authors of HerniaSurge was formed and, after consensus, the most relevant topics were chosen and the related KQs prioritized on the basis of presence of new RCTs or systematic reviews and meta-analyses. Ten of the 28 chapters were selected for the update.

### Chapter development group composition and stakeholders’ involvement

Teams of 4–6 members were created to perform the task of updating individual chapters (*Table 1*). At least two prior authors of the expired guidelines were invited for each chapter. Young surgeon researchers were added to join these teams where possible. A total of 18 HerniaSurge experts and 12 new members were appointed. The same group voted on recommendations after discussion.

**Table 1:** Team composition of the updated guideline on groin hernia management

Chapter	Team
6a. Tissue repair	Lorenz (DE), Wiessner (DE), Chen (USA), Miserez (BE)
6d. Open preperitoneal repair	Berrevoet (BE), Lopez-Cano (ES), Garcia-Alamino (ES), Lorenz (DE)
6f. Laparo-endoscopic repair	Simons (NL), Köckerling (DE), Lopez-Cano (ES), Tran (AUS), Verdauguer (ES)
8. Occult	DeBeaux (UK), Burgmans (NL), Reinpold (DE), East (CZE), Stabilini (IT)
10. Mesh	Burgmans (NL), Köckerling (DE), Montgomery (SE), Kukleta (CH)
12. Antibiotic prophylaxis	Kockerling (DE), Montgomery (SE), Henriksen (SE), Aufenacker (NL)
13. Anaesthesia	Agresta (IT), van Veenendaal (NL), Sartori (IT), Simons (NL)
19. Chronic pain treatment	Miserez (BE), Zwaans (NL), Loos (NL), Pawlak (UK), Aasvang (DK), van Veenendaal (NL), Chen (USA)
21. Emergency	Pawlak (UK), de Beaux (UK), Agresta (IT), Podda (IT), East (CZE), Morales-Conde (ES)
28. Non-commercial mesh	Sanders (UK), Berrevoet (BE), Oppong (UK), Yeboah (GH), Simons (NL)

AUS = Australia; BE = Belgium; CZE = Czech Republic; DE = Germany; DK = Denmark; ESP = Spain; IT = Italy; NL = Netherlands; SE = Sweden; UK = United Kingdom; USA = United States of America; GH = Ghana; CH = Switzerland.

It is acknowledged that a certified guideline methodologist would have been preferred to help inform these guidelines; however, due to the coronavirus disease 2019 (COVID-19) pandemic this was not practical. Cochrane experts were consulted for the literature search and provided training on Grade methodology. Subsequently we have relied on the group experience in guideline methodology.

Conflicts of interest were expressed prior to updating the guideline and the numbers of experts meant that a wide breadth of experience was available for recommendations. In each chapter, a balance was sought among members with strong opinions and neutral members. The former were deemed crucial to select and appraise the evidence, the latter were involved in the draft of the chapter to avoid influence and bias coming from strong opinions. In all cases recommendations and statements were presented to the whole panel and subsequently voted through online anonymous surveys.

The composition of the group was planned also according to the multidisciplinary aspect of some of the key questions. Two anaesthetists (N.V.V., E.A.), both experts in abdominal wall and pain management, were involved from the beginning in the update process and were responsible for the chapter on anaesthesia and chronic postoperative pain. Two representatives of low-income countries (M.Y., C.O.) were also included in the group that analysed literature on alternative meshes in low-resource settings.

Patient representatives were involved thanks to a spontaneous group formed on Facebook called ‘Hernia Patients Support Group’ that EHS helps facilitate. This group comprises 3000 members that have had hernia surgery or are on a waiting list for abdominal wall defect and officially engages with EHS. A formal call was launched, and five patients joined the working group for consultation. They were asked to rate the most relevant outcomes according to their values and preferences as well as the thresholds for the decision on these outcomes.

They received written materials in plain English, explaining methodology, KQs, basics of treatment options, main findings of the literature review as well as the recommendations. The document containing the manuscript was also provided for evaluation.

Finally, the recommendations were discussed with them in an online meeting with the steering committee to explore level of agreement, suggestions, patients’ perspectives and values pertaining to the final statements and recommendations.

It was not possible to include patient representatives from low-income countries.

These guidelines are an update from the level 1 publications that informed the original guidelines. It was decided that the same methodology would be used from the HerniaSurge 2018 guidelines. The current standards for guideline production are changing and new tools for evidence appraisal are available with better external validity and reliability (AMSTAR 2, RoB2, ROBINS-I). The steering committee decided to adopt the same tools already used in the older version of HerniaSurge. In order to be consistent with the past document, the Scottish Intercollegiate Guidelines Network (SIGN) checklists were adopted in the preparation of this update.

A literature search was performed for all level 1 evidence and large registry studies using the search term ‘inguinal hernia’ and recorded in Endnote reference manager. The search was performed in PubMed, PubMed Central, MEDLINE, The Cochrane central registry of controlled trials, Google Scholar and Embase. The last literature search was performed on 1 April 2022. Additionally, all teams conducted literature searches. Each team analysed the search results, made a final selection of articles (according to the PRISMA flowcharts), analysed the included articles and created evidence tables. This process started in June 2020 and ended August 2022.

The principles of guideline development were followed according to SIGN, Grading of Recommendations, Assessment, Development, and Evaluations (GRADE) and the Appraisal of Guidelines for Research and Evaluation (AGREE) instrument. Where possible, Patients, Intervention, Comparator, Outcome (PICOs) were developed for comparison of the techniques individually or clustered. The search terms, PICOs, PRISMA charts and tables with articles are published in Supplementary material.

Due to the COVID-19 pandemic, there were some delays in the process and face-to-face meetings were not possible.

During a second online meeting, an expert consensus meeting was organized. The results of each chapter were presented and discussed by all members. A modified Delphi method was used to vote on all statements and recommendations. Refraining from voting was not allowed. Consensus was defined as at least 70 per cent agreement among experts. All statements and recommendations that did not reach consensus were re-evaluated by the responsible teams. The content was reconsidered and/or reformulated. After revisions, a follow-up expert consensus meeting was held online (August 2022). Revised statements and recommendations were presented and voted on by all experts. Finally, consensus was reached on all statements and recommendations of the updated guideline on groin hernia management among the experts (*Table 2*). The consensus methodology used for the updated guideline was similar as in the previous guideline^2^. A total of 23 statements and recommendations were presented at the EHS meeting in Manchester on 21 October 2022 and voted on in order to get feedback and comments from delegates.

**Table 2:** Level of consensus after each expert consensus meeting

First expert consensus meeting	Second expert consensus meeting	Third expert consensus meeting
Consensus: 71 items No consensus: 14 items	Consensus: 33 items No consensus: 7 items	Consensus: 39 items No consensus: 0 items

According to EHS strategy, this is possibly the last update of guidelines as a set of several key questions. In the future, each new updated chapter will be published as a separate document to allow the easier update of a single KQ instead of the entire Guideline.

Each chapter in the present update is structured as in a traditional guideline:

- A summary is provided to help the reader in understanding the process and the challenges encountered in the preparation of the evidence and their appraisal.
- A grid with the final recommendation, level of evidence and strength of recommendation (statements are included whenever needed as findings supporting the recommendation).
- A general introduction.
- Results of evidence search and detailed description of relevant data.
- Discussion with evidence appraisal containing the criteria used to produce the updated recommendation. According to the GRADE method, scientific evidence is not the only guidance but other factors (patients’ values, desirable and undesirable effects, balance among them, cost effectiveness, acceptability, equity and feasibility) are incorporated in the process to inform decisions in a structured and transparent manner.

**Chapter 6a–b. Mesh or non-mesh and best non-mesh repair**

**Key Question 1: Which is the preferred repair method for inguinal hernias: mesh or non-mesh?**

**Key Question 2: Which non-mesh technique is the preferred repair method for inguinal hernias?**

**Table:** Updated Statements and Recommendations

	Text	Level of evidence	Strength of recommendation
KQ 1			
Statement	Mesh and non-mesh repairs are effective surgical approaches in treating groin hernias, each demonstrating benefits in different areas.	☒☒☒☐	
Statement	Mesh-based repair reduces the risk of recurrence without increasing the risk for chronic pain.	☒☒☒☐	
Statement	In selected groups of patients with primary unilateral inguinal hernia repair, the Shouldice technique achieves one-year outcomes comparable to that of Lichtenstein, TEP and TAPP operations providing expertise and competence are available.	☒☒☐☐	
Recommendation	A mesh-based repair technique is recommended for the majority of patients undergoing inguinal hernia repair.	☒☒☒☐	Strong
Recommendation	A non-mesh repair for inguinal hernia repair can be suggested after careful patient selection and shared decision-making if expertise is available.	☒☐☐☐	Weak
KQ 2			
Statement	The Shouldice technique has lower recurrence rates than other suture repairs.	☒☒☒☐	
Statement	The Desarda technique has a shorter learning curve compared to the Shouldice technique with favourable preliminary outcomes. There is insufficient high-quality long-term data on recurrence and chronic pain to make recommendations on generalized adoption.	☒☒☐☐	
Recommendation	The Shouldice technique is recommended in non-mesh inguinal hernia repair.	☒☒☒☐	Strong

##  

### Introduction

In the HerniaSurge guidelines, a mesh-based technique was recommended as first choice for all groin hernias^1^. It was stated that there was not enough evidence to support the use of a Shouldice in L1 and L2 inguinal hernias unless a shared decision with the patient was made. More research was advised to help clarify this issue.

Subsequent to the publication of the International HerniaSurge Guidelines, there has been global interest and public concern regarding the possible deleterious effects of mesh^8^. Patients, healthcare providers and surgeons have shared their concerns over potential risks associated with mesh repair and possible consequences for patients. There are scientific, social, medicolegal, economic, societal and personal implications surrounding this issue.

In this chapter, the evidence is updated with the same key questions as in the original HerniaSurge guidelines. In this introduction a summary of the evidence concerning factual and feared adverse effects of mesh use is offered. The potential risks of mesh are also extensively described in the HerniaSurge Guidelines chapter 10, which is not being updated this year^1^.

It is important to reiterate that the literature demonstrates the benefit and safety of mesh prostheses. However, the following complications of mesh repair, whether due to prosthetic or surgical technique, have been observed and should be taken into consideration when advising patients’ treatment options. Mesh, especially small pore meshes and three-dimensional mesh gadgets, have been found to shrink, migrate, or erode into adjacent structures, serving as a common mechanism for chronic post-inguinal hernia repair pain^8–11^. Dysejaculation and pain associated with sexual activity have been reported as a complication of mesh inguinal hernia repair, although other studies have demonstrated an improvement in sexual function and fertility with hernia repair^10,12^. Mesh repair, especially with preperitoneal mesh placement, confers the potential for rare visceral complications because of the proximity to adjacent organs including the colon, small intestine and bladder^13,14^. Preperitoneal mesh repair can complicate the performance of future radical prostatectomy, especially in the non-minimally invasive era of open prostate surgery^15,16^. Finally, the potential for true mesh allergy seen in autoimmune/inflammatory syndrome induced by adjuvants (ASIA)/Schoenfeld syndrome must be considered, although such cases are extremely rare relative to the global volume of mesh-based inguinal hernia repair^17,18^. Recognizing that these potential complications are infrequent, they can cause concern to such an extent that patients and surgeons in a shared decision process decide a non-mesh repair would be preferable.

**Key Question 1: Which is the preferred repair method for inguinal hernias: mesh or non-mesh?**

### Results

The search yielded 22 relevant publications: 1 guideline^19^, 8 systematic reviews with meta-analysis^14,20–26^, 8 randomized controlled trials^27–34^, 3 database analyses^35–37^, 1 review and 1 cross-sectional study.

The quality of the articles was scored using SIGN checklists by two authors individually and where there was discrepancy a consensus agreement was reached among all four authors regarding quality. Key questions were formulated and answered with available evidence. Statements and recommendations were made depending on the strength of the evidence and on consensus of the Guidelines group.

Since publication of the International HerniaSurge Guidelines for groin hernia management there were two systematic reviews with meta-analysis^21,38^ and one database analysis with high quality^36^. The other five systematic reviews with meta-analysis, nine randomized controlled trials, two database analyses, one review, and one cross-sectional study were of acceptable quality.

#### High-quality systematic reviews, meta-analysis and database studies

A recently updated 2018 Cochrane meta-analysis of RCTs on the use of mesh *versus* no mesh in inguinal (and femoral) hernia repair (studies included up to 9 May 2018) concluded that mesh and non-mesh repairs are effective surgical approaches in treating hernias, each demonstrating benefits in different areas. Compared to non-mesh repair, mesh repairs reduce the rate of hernia recurrence and neurovascular injury. Non-mesh repair is favoured because of less seroma formation and in low-income countries due to significantly lower cost and lack of availability of meshes.

#### Recurrence

Current data show persistent high recurrence rates over 10 per cent with all operation techniques in more than 300 000 patients in registry data (Mayo Clinic, ACS-NSQIP, Premier Database)^39^. Mesh reduces the risk of recurrence (moderate quality of evidence) despite higher seroma formation. In absolute numbers, one hernia recurrence was prevented for every 46 mesh repairs compared with non-mesh repairs^19,20^. In a Database registry analysis of female patients, no significant differences in the recurrence rate were reported between Shouldice, transabdominal preperitoneal (TAPP) and totally extraperitoneal (TEP) hernia repairs^35^.

The long-term follow-up update from the RCT by Barbaro *et al*.^31^ reported a 20-year recurrence rate of 9.7 per cent for the Shouldice operation^31^. This was quite favourable *versus* a recurrence rate of 25.7 per cent for the TEP procedure. However, while this study gives a unique longitudinal assessment of the well-established Shouldice technique, it likely misrepresents the efficacy of the standardized minimally invasive TEP repair found in modern practice. The authors stress that at the time of the initial study (1992–1994), laparoscopic (hernia) repair was still developing without a standardized technique, which contributes to the unfavourable and inconsistent results for TEP^31^.

#### Chronic pain

A meta-analysis and network analysis of all available RCTs in inguinal hernia repair showed no differences in the presence/severity of chronic pain between Shouldice, Lichtenstein and laparoscopic repairs, with up to 5 years postoperative follow-up^25^. With respect to possible male infertility after surgery, mesh does not seem to have a negative effect^27^.

The 2018 database study by Köckerling showed that after 1 year, there was lower pain at rest and on exertion (but not requiring additional treatment) in favour of the Shouldice *versus* the Lichtenstein technique. When the Shouldice technique was compared with TAPP or TEP, no differences for these outcome parameters could be found^36^. The second study analysing only women did not show any difference regarding pain at 1 year between the Shouldice technique, TAPP and TEP. By contrast, the Lichtenstein technique had disadvantages *versus* TAPP and TEP in terms of pain on exertion^35^.

**Key Question 2: Which non-mesh technique is the preferred repair method for inguinal hernias?**

### Results

The search yielded 21 relevant publications: 1 high-quality systematic review^40^, 1 high-quality database study^36^, 1 database study concerning female patients of acceptable quality^35^, 1 database study comparing Lichtenstein with annulorrhaphy of acceptable quality^37^, 11 RCTs in which Desarda and Lichtenstein were compared, 1 RCT in which Shouldice and TEP were compared with 20-year follow-up^31^, 1 study on femoral hernias^27^, and 4 cohort studies concerning herniotomy (low level)^41–44^. The latter articles were best evidence but low quality level.

#### Shouldice repair

All statements and recommendations regarding the primacy of the Shouldice repair among non-mesh-based tissue techniques remain unchanged from the previous Guidelines. The Shouldice technique remains the best evaluated and best standardized non-mesh-based tissue repair.

A large database study reporting 1-year follow-up by questionnaire from Germany has shown no significant differences in selected inguinal hernia cases (mean age 40 years old, 30 per cent women, smaller defects < 3 cm, average BMI 24, and no risk factors) regarding the recurrence rate in Shouldice repair compared to TAPP, TEP and Lichtenstein^36^.

Shouldice repair has lower recurrence rates than other suture repairs and favourable outcomes in primary inguinal hernia repair. Recent data with only short- to medium-term outcomes have supported that Shouldice tissue repair is an acceptable choice for primary hernia repair under certain circumstances. There was one long-term-follow-up study after Shouldice repair under local anaesthesia performed by trainees with a recurrence rate of 2.88 per cent after 18 years (80 per cent follow-up) and moderate or severe pain of 1.8 per cent after 3 years^45^. Two high-quality database studies have shown for selected groups of patients with specific hernia characteristics (that is, smaller indirect and direct hernias <3 cm, female sex after exclusion of any femoral hernia, younger patients under 40, and lower average BMI of 24) that the Shouldice technique can be used for primary unilateral inguinal hernia repair if expertise is present, achieving 1-year outcomes comparable to that of Lichtenstein, TEP and TAPP operations^35,36^.

In addition to the updated Cochrane Review, a systematic review about the Shouldice technique was recently published along with a standardized protocol of the operation technique including clear key points under supervision of the Shouldice hospital^46^.

This paper identified the following indications for the Shouldice technique, suggested mainly based on low evidence:

- primary indirect and small direct inguinal hernias in young men (EHS-Classification LI, LII, MI) below 40 years
- primary indirect and direct hernias in women after ruling out femoral hernias (EHS-Classification LI, LII, MI, MII)
- recurrent indirect hernias following primary TAPP or TEP (EHS-Classification LI, LII–R1)^46^.

#### Desarda repair

In the HerniaSurge guidelines, the Desarda repair did not have enough scientific evidence of acceptable quality to make any specific statements or recommendations. Several studies including RCTs, systematic reviews and meta-analyses report currently the equivalence of the Desarda and Lichtenstein techniques regarding recurrence. Several RCTs of different methodological quality comparing Lichtenstein and Desarda techniques in elective primary inguinal hernia repair have been published. There are three meta-analyses comparing the Desarda and Lichtenstein techniques with acceptable quality^22,23^ and one more recent meta-analysis with high quality^21^. Based on these data, the Desarda technique can achieve equivalent recurrence rates to Lichtenstein mesh repair.

There are no RCTs that directly compare the Desarda and Shouldice techniques. The meta-analysis by Bracale *et al*. indirectly compared the Desarda technique with the Shouldice technique by using studies that compared these techniques with the Lichtenstein repair^40^.

However, the available data on Desarda repair have some limitations and potential for bias. Only five RCTs report recurrence rates with a follow-up of 2 years or longer^28,29,33,47,48^. The quality of these studies, duration of follow-up and level of evidence is heterogeneous. Further high-level studies are needed to support these findings.

The role of the Desarda technique in patients with larger indirect hernias and especially direct hernias (with potential underlying collagen deficit) is unclear for the moment, not only with respect to the long-term outcome but also regarding technique. There is no clear standard protocol delineating limitations of the Desarda technique as well as operative technique and modifications for hernia subtypes (for example, opening of the transversalis fascia to exclude femoral hernias). In addition, all RCTs specifically excluded patients with a divided, thin or weak external oblique fascia, and although this is probably a minority, this is a rather subjective criterion that would confer a selection bias. Additionally, some studies excluded patients with chronic obstructive pulmonary disease (COPD), chronic cough and other co-morbidities. Finally, there does not seem to be a consensus on the suture technique and material used to fixate the strip of the released external oblique fascia cranially and caudally.

The data on the occurrence and severity of chronic pain between Lichtenstein and Desarda are neither designed or powered in the included studies to reasonably answer this question. Due to the lack of a clear definition and timing of evaluation of chronic pain, they have not been included in the present meta-analyses. All comparative RCTs have been performed using a standard ‘normal pore’ polypropylene mesh in the Lichtenstein arm, whereas it has been suggested in previous guidelines and confirmed recently that the use of large pore meshes in the Lichtenstein technique is beneficial in decreasing the rate of moderate/severe chronic pain or foreign body sensation^16^. For other operative parameters and perioperative outcomes such as operation time and early convalescence, the Desarda technique demonstrates some benefit^32^, although this finding is not universal and is not reflected in the current meta-analysis.

For now, the Desarda technique is an interesting option as a pure tissue repair because of its simplicity, based on a low number of small RCTs of mostly acceptable quality. As there is insufficient high-quality data on long-term recurrence rate, incidence of chronic pain and patient selection, it is too early to recommend this technique for everyday practice as an alternative to the well-established Shouldice repair.

##### Other pure tissue repairs

There is no current high-level evidence to provide specific statements or recommendations on other tissue-based techniques including Marcy, Moloney darn or Bassini as an alternative to the Shouldice repair. As they are still used in low-resource regions the evidence is described.

#### Annulorrhaphy/Marcy repair

High ligation of the inguinal hernia sac (Marcy repair) is a standard procedure for most paediatric hernias. There are a few mostly cohort studies that address annulorrhaphy with high ligation for 12–29-year–old male patients including long-term follow-up demonstrating low and acceptable recurrence rates and low cumulative reoperation rates^37,41–43^. Taking into consideration that the same group of young male patients has a higher risk of developing chronic pain after mesh repair, annulorrhaphy could be offered as an alternative for young men with small indirect inguinal hernias wishing to avoid a mesh-based repair, albeit with a known higher rate of recurrence (4.8 per cent on telephone follow-up) and reoperation rate of 8.1–14 per cent (median follow-up 15 years)^37^.

#### Moloney darn

A modified version of the older, but recently re-popularized, non-mesh Moloney darn technique demonstrates comparable outcomes to the Lichtenstein mesh technique, but the quality and validity of these studies do not support specific statements or recommendations. It remains problematic that there are several different ‘modified’ techniques, as described by the extensive systematic review by Finch *et al*.^24^. Analysis of the RCTs include two (low-quality) papers including 473 patients with a follow-up longer than 1 year demonstrating comparable outcomes with Lichtenstein for recurrence rate (between 0 and 1 per cent)^49,50^, but only the paper by Kucuk *et al*. reports on the incidence of chronic pain (0.6 per cent in the non-mesh technique), without sufficient details regarding methodology^50^. All other included studies are of insufficient quality.

### Discussion

The analysis of tissue-based inguinal hernia repairs especially in comparison to mesh-based techniques includes many different specific operations with significant heterogeneity in methodology and technique. Aside from the Shouldice repair, there is no clear standardization of patient selection, operative technique and decision-making based upon hernia subtypes. The specific non-mesh repairs with available evidence include Shouldice, Desarda, Marcy and Moloney darn techniques. There are no comparative RCTs between the various non-mesh techniques, particularly the Desarda and Shouldice techniques, and no comparative studies between minimally invasive and pure tissue procedures. Proficiency in surgical technique and patient selection make rigorous comparison challenging in even the highest-quality studies. As with all techniques, surgeons’ expertise will influence the results of comparative studies of all operation techniques^44^. HerniaSurge guidelines have recommended a tailored approach to inguinal hernia management including being proficient in offering patients both an anterior and a posterior approach^1^. As tissue repair can be indicated in cases of infection and in a shared decision with a patient it is recommended that surgeons master the Shouldice technique or refer patients to a surgeon experienced in the technique. The Shouldice is the best non-mesh technique, but has an unknown but long learning curve.

**Patients’ values and preferences inherent to Chapter 6a–b**

During the meeting patients were asked their perspectives and level of agreement or disagreement on the document. They agreed with the strength and direction of recommendations.

During discussion, the importance of the surgeon’s experience in performing tissue repair was highlighted, acknowledging the issue represented by the reduced number of surgeons trained in this type of procedure. Shared decision-making is crucial between surgeon and patient to select the optimal technique.

*Summary*

The HerniaSurge recommendation to use mesh in all adult patients was altered to the use of mesh in the majority of patients (consensus 88 per cent). Although there is high evidence that mesh repair is superior to non-mesh, there are cases in which a non-mesh repair can be suggested. Due to concerns regarding the use of permanent mesh, related to adverse events in other surgical fields, some patients search for surgeons who are prepared to offer tissue or non-mesh repairs. There are some clinical scenarios where the use of permanent mesh is contraindicated, for example in some infected operative fields. There are parts of the world where mesh is not available or affordable. There is some discussion concerning the value of non-mesh hernia repairs in young male patients with an L1–2 hernia. The evidence for this is very low and does not allow for a recommendation. Shouldice is the best non-mesh repair, although the experts agreed that it has a learning curve that should not be underestimated. In countries where mesh material is available it is infrequently used, and further training is needed but is not always readily available.

**Chapter 6d Update. Which is the preferred open-mesh technique for inguinal hernias: Lichtenstein or any open preperitoneal technique?**

**Key Question 1: Is there new evidence concerning open posterior (preperitoneal) *versus* open anterior repair (Lichtenstein) for inguinal hernias?**

**Key Question 2: Is there new evidence concerning open posterior (preperitoneal) *versus* laparo-endoscopic repair (TAPP or TEP) for inguinal hernias?**

**Table:** Updated Statements and Recommendations

	Text	Level of evidence	Strength of recommendation
KQ 1			
Statement	Currently available open preperitoneal mesh techniques can achieve comparable results in terms of recurrence rate compared to the Lichtenstein technique. There is not enough evidence to compare results between different open preperitoneal techniques.	☒☒☐☐	
Statement	Open preperitoneal mesh techniques can achieve favourable results in terms of operating time, acute and chronic postoperative pain and return to work compared to Lichtenstein repair.	☒☒☐☐	
Statement	There is no evidence regarding the best technique to treat recurrence after former open preperitoneal repair. Repair might be more complex as both the anterior and posterior anatomical planes may have been used in some of those techniques.	☒☒☐☐	
Recommendation	In open surgery a preperitoneal flat mesh technique seems to be an acceptable alternative, providing expertise and competence are available, with at least equal results as Lichtenstein repair.	☒☒☐☐	Weak
KQ 2			
Statement	No recommendation to advocate laparo-endoscopic preperitoneal mesh placement over open preperitoneal repairs can be made due to insufficient and heterogeneous data. However, there are patients and hernia characteristics that warrant a Lichtenstein or an open preperitoneal mesh technique as first choice.	☒☐☐☐	

### Introduction

In the HerniaSurge guidelines, it was suggested that open preperitoneal mesh repairs may result in less short-term postoperative and chronic pain as well as a faster recovery compared to the Lichtenstein repair. However, the use of these often non-flat meshes leads to higher costs and some of these approaches use both anterior and posterior anatomical planes. In this chapter the role of the open preperitoneal technique *versus* the open anterior repair and open preperitoneal technique *versus* posterior laparo-endoscopic approach are updated.

**Key Question 1: Is there new evidence concerning open posterior (preperitoneal) *versus* open anterior repair (Lichtenstein) for inguinal hernias?**

### Results

The search yielded 11 relevant publications (2 meta analyses, 7 RCTs^51–55^ and 2 registry analyses^56,57^). The quality of the articles was scored using SIGN checklists by two authors (F.B., R.L.) individually, and where there was discrepancy a consensus agreement was reached among all four authors with regard to quality.

Since publication of the HerniaSurge guidelines for groin hernia management, two meta-analyses are available^58,59^. They both concluded that there is at least equivalence of the different open preperitoneal techniques compared to the Lichtenstein repair. The seven RCTs showed comparable results as well, but most favour the open preperitoneal techniques in terms of pain^34,51,54,55,60^ and demonstrated quicker convalescence compared to an anterior mesh repair. These findings partly change the recommendations and conclusions published in the previous HerniaSurge guidelines. The concerns regarding the use of three-dimensional meshes such as in the Gilbert technique and the TIPP (transinguinal preperitoneal) technique are only theoretical and not evidence-based^56,57^.

The group of open preperitoneal techniques comprises several techniques. The specific transinguinal preperitoneal techniques include TIPP (Pelissier, Kugel), MOPP (minimal open preperitoneal), TREPP (transrectus extraperitoneal), the Onstep and the Gilbert technique. The evidence of all subgroups is low.

Conclusion update:

- Although the available evidence is rather heterogeneous concerning surgical techniques used for an open preperitoneal mesh placement, they are all at least comparable or favour the open preperitoneal techniques compared to the Lichtenstein approach in terms of recurrence rate, short-term postoperative pain and recovery time.
- Concerns regarding the use of three-dimensional or non-flat meshes (mesh plugs are not considered as a preperitoneal mesh technique) seem only theoretical and are not based on evidence. The dissection technique conducted in the plane used for eventual recurrent repair could be a complicating factor.

**Key Question 2: Is there new evidence concerning open posterior (preperitoneal) *versus* laparo-endoscopic repair (TAPP or TEP) for inguinal hernias?**

The search yielded four relevant publications (three RCTs^61–63^ and one observational comparative analysis^64^). The quality of the articles was scored using SIGN checklists by two authors (M.L.C., C.S.) individually, and where there was discrepancy a consensus agreement was reached among all four authors with regard to quality.

Since the publication of the HerniaSurge guidelines for groin hernia management only three studies comparing the TEP technique *versus* the open preperitoneal technique have been published. The three RCTs are of acceptable but low quality with a lack of information regarding bias control. They all showed comparable results between the laparo-endoscopic TEP and the open preperitoneal technique.

However, the analysed outcomes have been heterogeneous: activity parameters of the lower extremity muscles, quality of life or postoperative complications in the different studies. The comparator ‘open preperitoneal’ has also been heterogeneous, because in some studies an open approach has been used with maximum exposure of the preperitoneal space and in others only minimal exposure was required. Therefore, the results are impossible to interpret given the scarcity of data and patients analysed and no statement or recommendation can be made regarding the question whether in male patients with a unilateral primary inguinal hernia the preferred repair is a laparo-endoscopic or open preperitoneal technique.

Conclusion update:

- The available evidence remains heterogeneous and outcomes, although variable, seem to show equivalence between the open *versus* laparo-endoscopic preperitoneal repair techniques.
- These findings strengthen the statements published in the previous HerniaSurge guidelines.

**Patients’ values and preferences inherent to Chapter 6d**

Patients’ preferences are substantially concordant with panel recommendation direction and strength.

Their choice for intervention is connected to the minimization of adverse effects, improved recovery time and early discharge from hospital.

A unanimous concern is expressed over those techniques that, violating both preperitoneal space and inguinal canal, can be difficult to manage if recurrence occurs.

*Summary*

In the HerniaSurge guidelines, it was suggested that the open preperitoneal mesh repairs may result in less short-term postoperative and chronic pain as well as a faster recovery compared to the Lichtenstein repair. However, the use of these often non-flat meshes leads to higher costs and some of these approaches use both anterior and posterior anatomical planes. In this update it is concluded that there is no scientific evidence that open preperitoneal techniques (of different types) are inferior to Lichtenstein hernioplasty. Indeed, some studies report slightly less postoperative pain. There is no evidence that a recurrence after a preperitoneal mesh repair is more challenging or has a higher risk of complications. Statements that open preperitoneal mesh techniques might show favourable results in terms of operation time, short-term postoperative pain and convalescence compared to Lichtenstein repair and that there is no evidence that the use of non-flat or pre-shaped meshes leads to more postoperative complications received consensus (72 per cent). The recommendation that preperitoneal techniques can be suggested as a good option compared to Lichtenstein repair received a consensus of 72 per cent and after discussion the experts in these techniques (diverse and with follow-up of 3 years) advised that they can be suggested as an alternative to a Lichtenstein repair. However, HerniaSurge and the current WG have a majority albeit only expert opinion that the open preperitoneal technique could have a major downside in comparison to Lichtenstein, as dissection often goes through the groin anteriorly, has a longer learning curve than Lichtenstein repair and uses more frequently specifically engineered meshes, which makes the technique more expensive. No recommendation can be made comparing open preperitoneal techniques to TEP or TAPP, although one RCT was published after our deadline^65^.

**Chapter 6f. Open (Lichtenstein) *versus* laparo-endoscopic repair in unilateral uncomplicated inguinal hernia repair**

**Key Question: When considering recurrence, pain, learning curve, postoperative recovery and costs, which is the preferred technique for primary unilateral inguinal hernias: best open mesh (Lichtenstein) or a laparo-endoscopic (TEP and TAPP) technique?**

**Table:** Updated statements and recommendations

	Text	Level of evidence	Strength of recommendation
Statement	When the surgeon has sufficient experience in the technique, laparo-endoscopic techniques do not take longer than Lichtenstein operations	☒☒☒☐	
Statement	When the surgeon has sufficient experience, no significant differences are observed in the perioperative complications needing reoperation between the laparo-endoscopic and Lichtenstein techniques.	☒☒☒☐	
Statement	Laparo-endoscopic techniques have less chronic pain and faster recovery than the Lichtenstein repair.	☒☒☒☐	
Statement	The direct operative costs for laparo-endoscopic inguinal hernia repair are higher. The difference decreases when the total community costs are considered and the surgeon has sufficient experience.	☒☒☒☐	
Statement	The learning curve for laparo-endoscopic techniques (especially TEP) is longer than for Lichtenstein. There are rare but severe complications mainly described early in the learning curve. It is imperative that laparo-endoscopic techniques be learned in a properly supervised manner in order to minimize complications.	☒☒☒☐	
Recommendation	For patients (all sexes) with primary unilateral inguinal hernia, a laparo-endoscopic technique is recommended because of a lower postoperative pain incidence and a reduction in chronic pain incidence, provided that a surgeon with specific expertise and sufficient resources is available. However, there are patient and hernia characteristics that warrant Lichtenstein as first choice (chapter 7 on individualization).	☒☒☒☐	Strong (upgraded)

### Introduction

The EHS guidelines advocate for open Lichtenstein and laparo-endoscopic inguinal hernia techniques (TEP and TAPP) as the best evidence-based options for repair of primary unilateral inguinal hernias, provided the surgeon is sufficiently experienced and resources needed are available for the specific procedure^1,66,67^.

TEP and TAPP are superior regarding recovery, postoperative pain and chronic pain. Furthermore, laparo-endoscopic techniques seem to be safe and cost-effective in high-volume centres and expert hands. Nonetheless, according to previous guidelines^1^ there is a well-documented difference in learning curve and initial costs favouring Lichtenstein.

However, the studies available in this area have some limitations. They include the lack of clear definitions or end points in pain evaluation, quality of the surgeon's technique and caseload per surgeon.

With the aim to update the key question, all meta-analyses and RCTs that compared laparo-endoscopic techniques with open techniques other than Lichtenstein must be excluded as well as those that enrolled patients other than primary unilateral inguinal hernias.

### Results

The search yielded 12 relevant publications: 4 randomized clinical trials^68–71^, 3 systematic reviews^72–74^, 2 meta-analyses^75,76^ and 3 registry analyses^77–79^. The quality of the articles was scored using SIGN checklists by two authors individually (M.L., M.V.) and where there was discrepancy a consensus agreement was reached among all four authors regarding quality.

Since publication of the HerniaSurge guidelines for groin hernia management, four randomized clinical trials have been published: two of acceptable quality^70,71^ and two of high quality^68,69^. Three of the RCTs concluded in favour of laparo-endoscopic techniques^68,70,71^ and one concluded that they are comparable in terms of recurrence and length of hospital stay as secondary outcomes^69^. Five systematic reviews and meta-analyses were found: three of high quality^72,75,76^ with advantages for laparo-endoscopic techniques compared to Lichtenstein repair. There were three registry analyses found with acceptable quality.

#### Systematic reviews and meta-analyses

Meta-analyses before 2015^80–83^ compared laparo-endoscopic techniques with all open procedures, except for a subgroup analysis from 2005^84^ that identified advantages with the Lichtenstein operation in terms of operating time, seroma formation and recurrences, although it was strongly influenced by a trial^85^ using a smaller mesh size than recommended^1,86,87^.

A recent systematic review and meta-analysis^72,75^ compared TAPP and TEP with Lichtenstein for primary unilateral inguinal hernias in both sexes and found better outcomes for laparo-endoscopic techniques in terms of pain (OR 0.41, 95 per cent c.i. 0.3–0.56, *P* ≤ 0.00001^72^), postoperative recovery and shorter hospital stay, with the same rates of recurrence (OR 1.14, c.i. 0.51–2.55, *P* = 0.76^72^).

Aiolfi *et al.*^73^ concluded both techniques were comparable in terms of chronic pain, recurrence and length of hospital stay, although including a minor percentage of rTAPP in their systematic review. Better outcomes for TAPP and TEP were also found in a late network meta-analysis^76^ with regard to early postoperative pain and chronic pain (TAPP/Lichtenstein RR = 0.36, 95 per cent c.i. 0.15–0.81; TEP/Lichtenstein RR = 0.36, 95 per cent c.i. 0.21–0.54), return to work, haematoma and wound infection, with a similar recurrence rate and hospital length of stay. However, results must be carefully considered because the study includes retrospectively analysed data from a prospective registry^77^ with the largest number of patients included, which could strongly influence the outcome.

Gavriilidis *et al.*^74^ described a higher recurrence rate in the TEP group, including two controversial RCTs^85,88^ that highly influenced their results, one with smaller mesh size than recommended^85^ and the other including one outlying surgeon with higher rates of recurrences^88^, and once they were excluded no differences were found^86,87^.

#### RCTs

For comparison of the laparo-endoscopic (TEP, TAPP) with the open Lichtenstein technique for primary unilateral inguinal hernia many studies must be excluded as they included bilateral or recurrent hernias or compared TEP and TAPP with other open procedures^31,89–93^.

The preceding guidelines^1^ described advantages for laparo-endoscopic techniques in terms of postoperative pain^94^, analgesic consumption and postoperative recovery, with similar recurrences^88^ and operative time in expert hands^95–97^. Direct costs were found to be higher for TEP and TAPP even though the difference decreased when all community costs were evaluated^98^.

Recent RCTs fulfilling the inclusion criteria^68–71^ reinforce the advantages for laparo-endoscopic techniques in the comparison of 469 Lichtenstein operations with 483 laparo-endoscopic procedures.

Postoperative early pain was found to be lower in TEP and TAPP (visual analogue scale score for TEP/Lichtenstein at 24 h of surgery 2.24 ± 1.1 *versus* 2.64 ± 1.3 *P* = 0.005^70^; visual analogue scale score at 10 days for TAPP/Lichtenstein 1.4 ± 0.2 *versus* 2.8 ± 04, *P* < 0.05^71^). Likewise, chronic pain was inferior for laparo-endoscopic groups (TAPP/Lichtenstein 3.6 per cent *versus* 32.1 per cent, *P* < 0.003)^71^. A similar rate of recurrence is reported (TEP/Lichtenstein at 3 years of follow-up 2.2 per cent *versus* 1 per cent, *P* = 0.360)^68–71^.

Recent studies do not report newer evidence about learning curve or direct/total costs, although Sevinç *et al.*^70^ describe a longer hospital stay for the Lichtenstein group (length of hospital stay for TEP/Lichtenstein 1.05 ± 0.256 *versus* 1.25 ± 0.530 days, *P* = 0.001) as a secondary outcome.

#### Large database studies

A 2019 analysis of the Herniamed registry compared the prospective data collected for patients undergoing primary unilateral inguinal hernia repair using Lichtenstein, TEP and TAPP repair^77^. A total of 57 906 patients met the inclusion criteria, including 1 year of follow-up. Comparison revealed disadvantages for Lichtenstein *versus* TEP regarding postoperative complications (3.4 per cent *versus* 1.7 per cent, *P* < 0.001), complication-related reoperations (1.1 per cent *versus* 0.8 per cent, *P* = 0.003) and chronic pain at rest and on exertion (5.2 per cent *versus* 4.3 per cent, *P* = 0.003; 10.6 per cent *versus* 7.7 per cent, *P* < 0.001). Similarly, it reports drawbacks for Lichtenstein in contrast to TAPP according to postoperative complications (3.8 per cent *versus* 3.3 per cent, *P* < 0.029) and chronic pain at rest and on exertion (5 per cent *versus* 4.5 per cent, *P* = 0.029; 10.2 per cent *versus* 7.8 per cent, *P* < 0.001).

Another study based on the Herniamed registry from 2016^78^ compared TEP *versus* Lichtenstein in primary unilateral inguinal hernias in men, with 17 388 patients included and 1 year of follow-up. On multivariable analysis, TEP was found to have benefits regarding operative complication rate (*P* < 0.01), pain at rest (*P* < 0.011) and pain on exertion rate (*P* < 0.001), with a similar recurrence rate (*P* = 0.146) and chronic pain rate (*P* = 0.560).

In 2019, Quispe *et al.*^79^ compared Lichtenstein and TAPP with dissimilar conclusions as no differences were detected between groups in complications or pain scores at 24 h and 8 days after surgery, despite the small number of patients included.

#### Guidelines

The 2018 EHS guidelines^1^ concluded Lichtenstein and laparo-endoscopic techniques have comparable operation times, perioperative complication rates needing reoperation and recurrence rates when the surgeon has sufficient experience in the respective techniques.

TEP and TAPP have benefits in terms of early and later postoperative pain and faster return-to-normal activities or work. Direct operative costs were found to be higher for laparo-endoscopic techniques but were comparable with Lichtenstein when considering quality-of-life aspects and total community costs. In addition, the evidence favours the learning curve for Lichtenstein repair. Open mesh procedures are the most cost-effective operation, although in cost–utility analyses including quality of life the endoscopic techniques may be preferable.

### Discussion

Current literature reinforces precursory guidelines^1^ assertions about laparo-endoscopic techniques having benefits in terms of acute and chronic postoperative pain and faster recovery. According to the latest publications, no differences were found in the outcomes between adult men and women. Both techniques have comparable operation times and perioperative complication rates needing reoperation.

Regarding long-term recurrence rate, as described in prior guidelines^1^, no differences were found between Lichtenstein repair *versus* TAPP and TEP techniques.

Not enough updated information has been reported to change previous statements about the learning curve. As it stands in preceding guidelines^1^, the learning curve for laparo-endoscopic repair, especially TEP, seems to be longer than that for the Lichtenstein technique, and ranges between 50 and 100 procedures, with the first 30–50 being most critical. There are rare but severe complications described and laparo-endoscopic techniques should be learned in a properly supervised manner.

As regards direct costs, no recent studies have been reported. Evidence prior to the present time reveals increased direct costs for laparo-endoscopic techniques, while they become comparable when numbness, chronic pain and quality of life are taken into consideration.

However, studies are heterogeneous, lack clear definitions of acute and chronic pain, quality of surgeon's technique, caseload per surgeon and lack of hernia classification, which make further recommendations difficult.

Large RCTs with good external validity and clear definition of variables and large-scale database studies are needed to clarify inconclusive endpoints to properly compare those techniques. Clear and objective definitions of variables and accurate description of follow-up and surgeon experience are needed. Similarly, further high-quality studies must elucidate the role of other open approaches, such as open preperitoneal repair, in comparison of laparo-endoscopic techniques.

These findings are concordant with the recommendations and conclusions published in the previous HerniaSurge guidelines but reinforce the role of laparo-endoscopic techniques in expert hands. HerniaSurge recommends a standardization of the laparo-endoscopic and Lichtenstein techniques, structured training programmes and continuous supervision of trainees and surgeons within the learning curve.

**Patients’ values and preferences inherent to Chapter 6f**

Patients’ preferences are substantially concordant with panel recommendation direction and strength.

During the online meeting, patients’ representatives underlined how their threshold to evaluate superiority of a treatment over another substantially differs from surgeons’ perspectives in terms of numerical value. A researcher's perception of a clinically meaningful statistically significant outcome may differ from the patients’ perspective.

They agreed on the importance of tailoring treatment to patients’ characteristics and expectations through shared decision-making.

*Summary*

The main recommendation from the HerniaSurge guidelines remains. If expertise and resources are available, the laparo-endoscopic repair methods (TEP/TAPP) offer a quicker recovery and less chronic pain for a simple primary unilateral inguinal hernia. The experts warn of a long learning curve compared to anterior techniques, and for the relative contraindications for TEP/TAPP in general surgical practice. In these situations, an open anterior repair method is the better option. Examples are after prostatic surgery, pelvic radiation, lower abdominal (pelvic) surgery, scrotal hernia, when local anaesthesia is indicated and in regions where expertise in TEP/TAPP is not available or resources are lacking. Tailoring to the patient, type of hernia and surgeons’ expertise is essential. The discussion focused on the fact that the Lichtenstein repair is not the only ‘open’ alternative. Consensus was 84 per cent. The literature on this key question almost exclusively compares TEP or TAPP with Lichtenstein repair. Other open techniques can be good alternatives (see chapters 6a and 6d).

**Chapter 8. Occult hernias and bilateral repair**

**Key Question: What is the best treatment for patients presenting with a contralateral occult hernia at the time of laparo-endoscopic unilateral inguinal hernia repair?**

**Table:** Updated statements and recommendations

	Text	Level of evidence	Strength of recommendation
KQ			
Statement	The repair of a concomitant occult hernia can increase the overall surgical risk of the procedure because of the second procedure but can avoid a second operation for the patient with the cost and anaesthetic risk.	☒☒☐☐	
Statement	The risk of progression from occult to symptomatic clinical defect is unknown but possible at a rate of 1.2% per year.	☒☐☐☐	
Recommendation	The decision whether to perform the repair of an occult contralateral hernia identified during a laparo-endoscopic repair of a unilateral hernia should be discussed with the patient at the time of informed consent.	☒☐☐☐	Weak

### Introduction

An occult hernia, as defined by the HerniaSurge Working Group, is an asymptomatic hernia not detectable by physical examination. Occult hernias can be a problem for the clinician in terms of both diagnosis and strategy during minimally invasive hernia repair because of an unclear balance between benefits and harms as well as a poorly studied natural evolution.

The situation represents a possible issue of informed consent with the patient who is not aware of the medical condition and the possibility of an adverse event involving the asymptomatic side.

### Results

Literature search identified 315 papers; after duplicate removal and screening 12 studies entered the final evaluation.

#### Intraoperative management of contralateral occult hernia

Among the nine selected studies, two meta-analyses^99,100^ and eight observational cohort studies^101–108^ (five of them were already included in the meta-analysis) were retrieved. According to the SIGN checklist all the papers were judged of acceptable quality.

Dhanani *et al.* analysed the results of the management of occult contralateral hernia found in 5000 patients with a starting diagnosis of unilateral primary inguinal hernia and undergoing minimally invasive repair. The meta-analysis included 12 studies from 2001 to 2020 (1 RCT) and created a Markov decision model to evaluate the consequences of exploration and contralateral hernia repair in comparison to expectant management. Overall, the incidence of occult inguinal hernias diagnosed at the time of laparo-endoscopic inguinal hernia repair was 14.6 per cent (TEP 21.4 per cent *versus* TAPP 13.5 per cent; *P* < 0.001); after pooling the results, when undergoing occult hernia repair, 71 per cent of patients would undergo an unnecessary repair and 10.5 per cent would experience a complication. Alternatively, if the hernia was left unrepaired, less than one-third of those patients would eventually require a second operation. Therefore, the model concluded that only around 5 per cent of all patients undergoing a unilateral inguinal hernia repair would benefit from contralateral exploration.

Park *et al.* analysed six studies involving 1774 adult patients to evaluate outcomes associated with prophylactic contralateral laparoscopic inguinal hernia repair in the population who present with a symptomatic unilateral inguinal hernia repair and an asymptomatic contralateral. All studies were retrospective, partially overlapping with the review by Dhanani *et al.*, and judged to have a low to moderate risk of bias. The results showed that unilateral repairs have less operative time and less postoperative pain. Statistical significance was absent for complications, length of hospital stay and postoperative return to normal activities among patients undergoing bilateral and unilateral repair. Based on these observations, the authors concluded that asymptomatic inguinal hernias can be repaired when found to prevent the need for another operation in almost a third of patients.

A multicentre retrospective study in robotic inguinal hernia repair^101^ on 462 patients undergoing rTAPP repair for unilateral inguinal defect found 57 contralateral occult hernias (12.3 per cent) that had a mesh repair. The operative time was higher if having contralateral repair, and the authors showed similar clinical outcomes between unilateral and unplanned bilateral repairs.

A retrospective study from Kou *et al.*^106^ analysed the results of inguinal exploration *versus* no exploration in patients undergoing laparoscopic catheter placement for peritoneal dialysis. The authors found 26/365 (7 per cent) occult hernias in the routine laparoscopic exploration group; 17 were repaired with TAPP. After a mean follow-up time of 33.5 ± 20.8 months (range 3.4–87.9 months), the rate of metachronous hernia in patients that had exploration was 0 for those submitted to repair, 5.6 per cent for those without evidence of hernia and 22.2 per cent in case of no repair of an evident hernia. Overall, the rate of metachronous hernia was statistically higher in patients who did not receive laparoscopic exploration (13.4 per cent *versus* 5.6 per cent).

Another retrospective study from Ota *et al.*^108^ analysed results from a cohort of 259 patients that had TEP inguinal hernia repair; among them there were 70 (27 per cent) patients who underwent repair of an occult contralateral hernia. The contralateral intervention took on average more time in the occult hernia group (166 ± 61 min *versus* 140 ± 50 min in the non-occult hernia group). The hernia recurrence rate had a trend towards less recurrence in the occult hernia repair group (0 *versus* 6, *P* = 0.13).

### Discussion

The occurrence of a clinical occult contralateral hernia is a likely event in the clinical setting with variable rates currently established at around 15 per cent but with various reported ranges from 7.3 to 50.1 per cent^99,100^. The particular features of this condition pose a specific dilemma to the clinicians in terms of strategy and prognosis.

The concomitant repair of an occult contralateral hernia is based on three main concepts:

- The added repair could have the same morbidity as the unilateral hernia repair.
- The risk of recurrence is similar or inferior to the risk of a clinically apparent hernia.
- The patients will develop symptoms associated with the progressing occult hernia and will require a subsequent procedure.

In terms of morbidity, bilateral procedures are more prone to complications than unilateral repair. Recent data from the Herniamed registry^109,110^ have also confirmed that in both TEP and TAPP the risk is doubled for reoperation, intraoperative and postoperative complications.

The evidence on morbidity in the management of occult contralateral hernias is mixed and heterogeneous; the earlier stage of presentation and smaller dimensions of the defect requiring an easier dissection could explain why some of the series reporting postoperative outcomes are similar among unilateral and bilateral repair in this setting^101^ as also summarized by the review from Park *et al.*^100^.

Little is known about the natural evolution of asymptomatic occult hernias. A recent systematic review^111^ on watchful waiting for asymptomatic or minimally symptomatic inguinal hernia in men has shown that this strategy is safe in terms of acute events and that one-third of the patients will cross over from expectant management within 1.5–3 years to surgery and that almost 70 per cent of them will do the same after approximately 7 years from the initial visit. The study highlighted that morbidity, mortality, pain and discomfort both in the elective repair and crossover groups are similar.

It is difficult to extrapolate these results to the occult contralateral hernias, even if the two scenarios are both early-stage hernias. The occult hernia is a preclinical defect that a patient is not aware of. It is unknown if this type of hernia will progress in the presence of promoting factors as shown in peritoneal dialysis patients^106^ or will remain asymptomatic. Nevertheless, from some studies^112,113^ a 1.2 per cent per year rate of progression from asymptomatic to symptomatic hernia is highlighted.

The Markov model from Dhanani *et al.*^99^ in particular showed that hernia repair in this population could be of less benefit than expectant management. Seventy-one per cent of cases would undergo an unnecessary procedure, 10.5 per cent would suffer complications, while only one-third of those not operated would ask for a second intervention in the long term. The authors of the meta-analysis concluded that only around 5 per cent of all patients undergoing a unilateral inguinal hernia repair would benefit from contralateral exploration.

Several factors should be considered in this clinical scenario, but the surgical technique plays an important role: while it is unlikely to advise open surgical exploration, the laparo-endoscopic techniques have different features and the ability to detect small initial defects (TEP 21.4 per cent *versus* TAPP 13.5 per cent *P* < 0.001)^99^. Exploration with TEP requires direct dissection of the myopectineal orifice and is more efficient in finding small defects, but can cause inadvertent damage and weakening of the region. TAPP exploration, even if less invasive, has limitations in the recognition of small defects and cord lipomas.

The quality of the studies included in the present guideline is acceptable overall. No new randomized controlled trial has been published on the topic. Nevertheless, the rating of the level of evidence can be considered low to very low because all the data come from retrospective cohorts and the single available RCT is downgraded for several methodological biases.

There is heterogeneity observed across all studies concerning methodology, outcomes considered and the technique to detect defects. In particular, several definitions of an occult hernia were provided in the studies along with new terms to describe early stage and metachronous defects, making a reliable pooling of the results impossible and highlighting the need for a future definition of what constitutes an occult contralateral groin hernia.

According to all the limitations of the current body of evidence it is not possible to give strong recommendations. The panel of experts agrees that a thorough discussion of the pros and cons of both expectant management and treatment should be discussed with the patients at the time of informed consent, including the specific risk connected to contralateral dissection along with the risk of chronic postoperative pain.

Despite low-quality evidence and a substantial risk of bias in the included studies, immediate repair of occult contralateral inguinal hernias diagnosed at the time of elective hernia repair is not justified. Following intraoperative diagnosis of an occult contralateral hernia, more than 70 per cent of these patients will not require treatment. Without contralateral exploration, less than 10 per cent are likely to present for contralateral repair. Immediate diagnosis and repair will result in more complications than expectant management.

**Patients’ values and preferences inherent to Chapter 8**

Patients’ preferences are substantially concordant with panel recommendation direction and strength.

During the online meeting patients’ representatives underlined how, despite the low level of evidence, they would be in favour of simultaneous repair. They agreed on the importance of discussing the possibility of concomitant occult hernia and options during preoperative informed consent.

*Summary*

The decision whether to repair an occult contralateral hernia found in the course of a laparo-endoscopic repair of a unilateral hernia should be discussed with the patient at the time of informed consent (consensus 84 per cent). With the limitations in the current body of evidence relating to this topic, it is not possible to give strong recommendations. The experts agree that a thorough discussion of the pros and cons of both expectant management and treatment should be done with the patients at the time of informed consent, highlighting the risk connected to contralateral dissection, and the risk of chronic postoperative pain *versus* the likelihood of a future contralateral hernia repair.

**Chapter 10. Meshes**

**Key Question 1: What mesh type (characteristics) is the most suitable for open repair (Lichtenstein)? Is there new evidence concerning recurrence rate and chronic postoperative pain?**

**Key Question 2: What mesh type (characteristics) is the most suitable for laparo/endoscopic repair? Is there new evidence concerning recurrence rate and chronic postoperative pain?**

**Table:** Updated statements and recommendations

	Text	Level of evidence	Strength of recommendation
KQ 1–2			
Recommendation	According to the definition used in most RCTs, even if not universally accepted, the proposed thresholds to differentiate among polypropylene mesh types according to weight is <50 g/m^2^ for lightweight and >70 g/m^2^ for heavyweight meshes.	☒☒☐☐	Strong (upgraded)
Statement	The use of LWM reduces chronic postoperative pain and foreign body sensation compared to HWM in Lichtenstein repair.	☒☒☒☒	
Statement	The recurrence rate is not affected by a LWM in comparison to HWM in Lichtenstein repair.	☒☒☒☐	
Statement	In Lichtenstein repair, the recurrence rate is higher after using partial absorbable LWM compared to regular LWM and HWM.	☒☒☐☐	
Recommendation	In Lichtenstein repair an LWM is recommended to reduce the occurrence of chronic postoperative pain and foreign body sensation.	☒☒☒☒	Strong
Statement	The risk of recurrence is not affected by mesh weight in case of laparo-endoscopic repair of small and lateral defects.	☒☒☒☐	
Statement	The occurrence of chronic pain is not affected by mesh weight in laparo-endoscopic hernia repair.	☒☒☐☐	
Recommendation	In laparo-endoscopic repair an HWM is recommended, especially in a large and direct hernia, to reduce the risk of recurrence. LWM is not recommended as it does not reduce the risk of postoperative pain but increases risk of recurrence.	☒☒☒☒	Strong (upgraded)

### Introduction

Lightweight meshes (LWM) were introduced and further developed with the aim of minimizing chronic pain and the feeling of a foreign body in the groin. This has been an important research field in the last decade. The concept is that a highly engineered mesh with a tensile strength similar to native tissue and reduced material could offer a durable repair and better tissue integration. This may also reduce the risk of chronic postoperative inguinal pain (CPIP). Although CPIP is multifactorial in its origin, the reduction of the amount of scar tissue, foreign body reaction and shrinkage related to heavyweight meshes (HWM) was the basis on which the postulated effect of LWMs was tested. Under the ‘LWM brand’, several devices have been launched, making mesh classification difficult and generating problems comparing outcomes. There is no clearly defined weight limit for LWM and HWM. However, most RCTs use <50 g/m^2^ for LWM and >70 g/m^2^ for HWM, leaving an indeterminate area for meshes between these two levels.

In the last version of the HerniaSurge guidelines the effect of LWMs on pain was considered limited only to the early postoperative period (6 months) for open surgery and absent when using laparo/endoscopic techniques. New evidence has been published in the time frame from the latest analysis on the topic. The aim of this review was to update the recommendations on mesh types to be used in open and laparo-endoscopic hernia repair techniques.

**Key Question 1: What mesh type (characteristics) is the most suitable for open repair (Lichtenstein)? Is there new evidence concerning recurrence rate, chronic postoperative pain?**

### Results

#### Open surgery (Lichtenstein)

In total, five new RCTs^114–118^, one systematic review with meta-analysis (including the RCTs)^119^ and two registry-based studies (Swedish Hernia Register)^120,121^ comparing LWM to HWM in open hernia repair were identified. The RCTs were scored as acceptable or low-quality according to SIGN. All RCTs confirmed a similar recurrence rate for LWM and HWM and a similar occurrence of pain-related outcomes^114–118^. However, two studies showed a reduced foreign body sensation in favour of LWM^116,118^.

The systematic review by Bakker *et al.*^119^ was scored as high quality according to SIGN. A total of 26 papers (including 21 RCTs) reported on 4576 patients. This meta-analysis found no difference between LWM and HWM for severe pain (RR 0.73; 95 per cent c.i.: 0.38–1.41) or recurrence (RR 1.22; 95 per cent c.i.: 0.76–1.96). A significant reduction was seen for ‘any pain’ comparing LWM *versus* HWM (RR 0.78; 95 per cent c.i.: 0.64–0.96) lasting 12 months after surgery. This significance disappeared at long-term (24 months) follow-up (FU). The ‘feeling of a foreign body’ was attenuated in patients having an HWM (RR 0.64; 95 per cent c.i.: 0.51–0.80). This review reported an evaluation of evidence according to GRADE methodology. Outcomes for ‘any pain’ and ‘foreign body sensation’ constituted a high level of evidence, whereas ‘severe pain’ a moderate level of evidence and ‘recurrence’ a low level of evidence. The Trial Sequential Analysis (TSA) of this review (unpublished data) indicated an increased risk of any chronic pain and foreign body feeling when using HWM. The TSA also reported a shortage of evidence for recurrence due to a low event rate. Therefore, there is no need to perform further RCTs that compare LWM and HWM for open inguinal hernia repair.

Data on open mesh repair with LWM *versus* HWM were analysed in two studies using data from the large Swedish population database^120,121^. The first study^120^ analysed chronic pain at 12 months with questionnaires sent to 23 259 male patients after Lichtenstein repair for a unilateral inguinal hernia. HWM > 50 g/m^2^ were compared to various types of LWM < 50 g/m^2^ (regular LWM polypropylene, partially absorbable LWM with poliglecaprone or partially absorbable LWM with polyglactin). There was no difference in chronic pain at 12 months between mesh types used after surgery in a multivariable analysis performed.

In a second study^121^ using the same database, factors predicting reoperation for recurrence were analysed. Only partially absorbable LWM (with absorbable poliglecaprone or polyglactin) resulted in a significant increased risk of recurrence compared with HWM (HR 1.42–2.05, *P* < 0.001). The difference disappeared when a single-material (polypropylene) LWM was used (HR 1.12, 95 per cent c.i. 0.96–1.31).

**Key Question 2: What mesh type (characteristics) is the most suitable for laparo/endoscopic repair? Is there new evidence concerning recurrence rate, chronic postoperative pain?**

#### Results

Five new RCTs^122–126^, four systematic reviews^127–130^ (including the RCTs) and one registry-based study^131^ were identified comparing LWM to HWM in laparo-endoscopic repair. The RCTs were scored according to SIGN as either of acceptable or of high quality. All trials reported similar occurrence of pain and higher recurrence rates using LWM.

Two systematic reviews were scored as acceptable according to SIGN evaluation^128,129^. The systematic reviews by Bakker *et al*.^127^ and Xu and Xu^130^ both scored high quality, but the latter dealt with LWM in TEP only. The evidence delivered by Bakker *et al*. is more recent and complete and formed the basis for this update.

Twelve RCTs, encompassing 2909 patients (LWM 1490 *versus* HWM 1419), were included in the meta-analysis. The risk of a recurrence was increased after LWM (RR 2.21; 95 per cent c.i. 1.14–4.31), especially in non-fixated mesh used in direct inguinal hernia repairs (RR 7.27; 95 per cent c.i. 1.33–39.73) and/or large hernia defects. Specifically, if studies that only performed mesh fixation were included, increased risk of recurrence when using LWM disappeared (RR 1.20–95 per cent c.i.: 0.40–3.61; *I*^2^ 5 per cent) regardless of whether the hernia was indirect or direct. The same meta-analysis demonstrated that the major contribution to this effect was observed in non-fixated direct hernia when an LWM was adopted in comparison to an HWM (RR 7.27–95 per cent c.i.: 1.33–39.73). TSA showed that data are still insufficient to draw conclusions concerning mesh fixation. No difference was seen regarding ‘any pain’ (RR 0.79; 95 per cent c.i.: 0.52–1.20), ‘severe pain’ (RR 0.38; 95 per cent c.i.: 0.11–1.35) and ‘foreign-body sensation’ (RR 0.94; 95 per cent c.i.: 0.73–1.20) between LWM and HWM. No influence on pain outcomes was observed when using macroporous (>1 mm) meshes. TSA on the included studies showed firm evidence for recurrence, but shortage of evidence for pain as an outcome.

The level of evidence was rated according to GRADE. Concerning the outcome ‘recurrence’, the evidence was considered high in general but low for subgroups in direct, large hernias or when fixation was not adopted due to imprecision. Evidence was low for pain and foreign body sensation.

Data on the use of LWM in laparo-endoscopic surgery were also obtained from a population-based study from the Swedish Hernia Register by Melkemichel *et al.*^131^. Male patients undergoing TEP repair with either LWM or HWM were analysed for factors affecting reoperation for a recurrence. The risk was higher when an LWM was implanted (HR 1.56), particularly in large direct hernia defects (HR 1.75). No data on CPIP could be retrieved from the registries.

According to these findings the previous recommendations on LWM in laparo-endoscopic hernia repair are updated.

### Discussion

The results of the adoption of LWM are conflicting in both open and laparoscopic hernia repair. Several factors can affect chronic pain occurrence in inguinal hernia repair, such as operative technique, nerve handling, mesh type, mesh fixation and other patient- or postoperative-related factors. It is very simplistic to define the role of the single material in such a confused and multifactorial environment, but some evidence, even if conflicting, from the analysed studies has been observed.

In this update of guidelines, the statements and recommendations for open and laparo-endoscopic techniques were split to better highlight the different behaviour of LWM and HWM in relation to technique used for mesh placement.

#### Open anterior mesh repair (Lichtenstein)

In open surgery the effect on pain and foreign body sensation caused by LWM has become clearer from recent added trials with improved quality of evidence^119^. These findings have already been presented in previous systematic reviews but were not considered of clinical relevance^132,133^. The subsequent trials published strengthen the concept of LWM being able to reduce postoperative pain and foreign body feeling, both in early and late follow-up. LWMs have become a valid choice, supported by the strong recommendation made in these guidelines.

The issue of a possible higher risk of a recurrence after LWM is no longer relevant when regular, non-absorbable LWMs are used. The systematic review from Bakker *et al.* has shown that fixation of an LWM in Lichtenstein repair is sufficient to act as an efficient barrier both in small and large hernia defects^119^. These results were confirmed in a large Swedish database trial^121^. Only partial absorbable LWM with poliglecaprone or polyglactin might result in higher recurrence rates.

The 22 per cent reduction in chronic pain and the 36 per cent reduction of foreign body sensation using an LWM at open hernia repair with a similar recurrence rate is better for patient outcomes.

#### Laparo-endoscopic repair

The results of this update, even with heterogeneity, demonstrate the absence of an effect of LWMs on pain-related outcomes with laparo-endoscopic repair. However, the trial sequential analysis (TSA) detected a shortage of evidence to establish firm conclusions. Findings could be due to the laparo/endoscopic dissection technique or the mesh position in comparison to the open technique. The reduced trauma on muscular and aponeurotic tissue at preperitoneal dissection and mesh location might preserve the most sensitive structures from being exposed to a large foreign body reaction induced by a mesh.

A potential increased risk of recurrence when adopting LWM in laparo-endoscopic repair is a major drawback. Although the mesh has textile characteristics that make it resistant to burst when a tacker fixation technique is adopted, non-fixation in direct and large defects poses higher risk of failure for LWM with a 7-fold increase compared to HWM^127^.

Different strategies include using a larger mesh, mesh with higher reinforcement capacity or to glue/tack the mesh for fixation^115^. Glue causes little harm, but it is expensive and takes more time for application. The use of tacks has similar limitations but could also result in a higher risk of pain.

The statements rely mainly on high-quality systematic reviews, but with some limitations for quality concerning results. Publication bias is mainly absent, but several trials suffer from methodological flaws. The patient selection criteria were heterogeneous among studies. The definition and measures of pain and foreign body sensation were not uniform across studies, leading to a potential inconsistency of results. Some studies had a shorter FU, leading to a possible underestimation of the true recurrence rate.

The systematic reviews included were of high quality and the overall sample size was sufficient to draw well-founded conclusions. Moreover, subgroup analysis and TSA compensated for some of the limitations, producing reliable evidence to support the statements and recommendations presented.

**Patients’ values and preferences inherent to Chapter 10**

Patients’ preferences are substantially concordant with panel recommendation direction and strength.

They point out that for open surgery, even if transient, the reduction in almost 20 per cent of pain at 12 months and 30 per cent in foreign body sensation is clinically meaningful and they are in favour of lightweight meshes.

*Summary*

There was a long discussion on the important characteristics of synthetic mesh for inguinal hernia repair. There was general consensus among the experts that the weight (or better, ‘density’ reported as g/m^2^) of the mesh is a poor parameter to predict its tissue integration and performance. Large pore size (>1000 µm), perhaps in combination with ‘weight’ (and other characteristics), should be used to define the possibility of successful integration of a mesh at the same time as minimizing events that can lead to mesh shrinkage or the sensation of a foreign body. Nevertheless, a number of studies (high quality) comparing mesh in open and in laparo-endoscopic repair all use weight (lightweight, LWM and heavyweight, HWM) as the comparator. That is despite some of the lightweight meshes being partially resorbable, adding a further variable to the mix. Furthermore, most LWMs in these RCTs are of pore <1000 µm. However, some LWMs can be ‘too light’ and lead to bulging of the mesh or mesh rupture. The experts agreed that according to the definition used in most RCTs, even if not universally accepted, the proposed thresholds to differentiate among mesh types according to weight are <50 g/m^2^ for lightweight and >70 g/m^2^ for heavyweight meshes (consensus 74 per cent). With high-quality systematic reviews, it is concluded that in Lichtenstein repair an LWM is recommended to reduce the occurrence of chronic postoperative pain and foreign body sensation; in laparo-endoscopic repair an HWM is recommended in a large and direct hernia to reduce the risk of recurrence and in laparo-endoscopic repair; LWM does not appear to reduce the occurrence of early and chronic postoperative pain, so HWM can be suggested in all laparo-endoscopic repairs. All received high consensus. However, there are many meshes on the market, with different pore size, weight, weave patterns and so on, that have never been tested in an RCT, making generalization of these findings scientifically challenging.

**Chapter 12. Antibiotic prophylaxis**

**Key Question: Value of prophylactic antibiotics in open or laparo-endoscopic techniques. Is there new evidence concerning the indication for prophylactic antibiotics in open anterior or laparo-endoscopic inguinal hernia repair?**

**Table:** Updated statements and recommendations

	Text	Level of evidence	Strength of recommendation
KQ			
Statement	Inguinal hernia surgery appears to be currently conducted worldwide in a low infection risk environment.	☒☒☐☐	
Recommendation	Antibiotic prophylaxis is not recommended in elective open inguinal mesh hernia repair in average-risk patients in a low infection risk environment.	☒☒☒☒	Strong
Recommendation	Antibiotic prophylaxis is suggested in elective open inguinal mesh repair in high-risk patients in a low infection risk environment.	☒☒☐☐	Weak
Recommendation	Antibiotic prophylaxis is recommended in elective open inguinal mesh repair in any patient in a high-risk environment.	☒☒☒☒	Strong
Recommendation	Antibiotic prophylaxis is not suggested in elective laparo-endoscopic inguinal hernia repair in any patient and in any risk environment.	☒☒☒☐	Weak
Recommendation	First-generation cephalosporins and β-lactam/β-lactamase inhibitors are recommended as antibiotic prophylaxis.	☒☒☒☒	Strong

### Introduction

After the publication of the HerniaSurge guidelines^1^, the use of a mesh continues to be an argument in favour of antibiotic prophylaxis in hernia surgery. Two recent surveys have revealed that surgeons still administer antibiotic prophylaxis in inguinal hernia surgery^134,135^ even though the International Guidelines do not recommend this. That is also true for laparo-endoscopic inguinal hernia surgery^135^. Both surveys were conducted in a low-risk environment for surgical-site infections (SSIs)^134,135^. The surgeons in both surveys stated that their decision to administer antibiotic prophylaxis was in line with the scientific evidence.

The findings of these surveys demonstrate just how important it is to collate and evaluate all available data on the use of antibiotic prophylaxis in inguinal hernia surgery. Accordingly, this chapter now evaluates and reports on the new studies published between January 2015 and November 2021 on antibiotic prophylaxis and SSIs in inguinal hernia surgery for their relevance to the update of the HerniaSurge guidelines^1^. The HerniaSurge guidelines stated that antibiotic prophylaxis in average-risk patients in low-risk environments is not recommended in open surgery. In laparo-endoscopic repair it is never recommended. The update aimed to answer the following key question on the basis of the new studies.

### Results

**Fig. 1:**
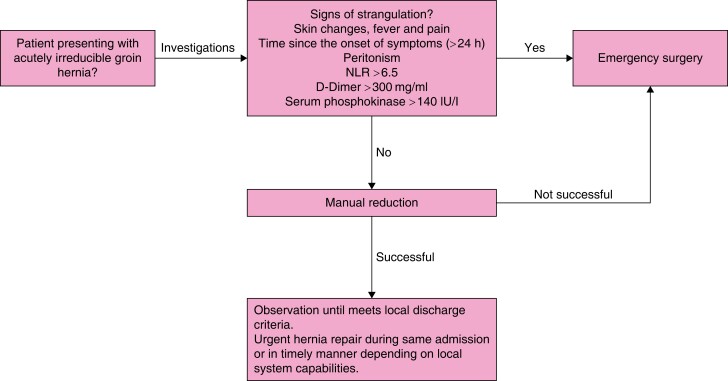
The proposed algorithm for the treatment of acute hernias

In addition to the surveys cited above^134,135^, the search yielded 12 relevant publications (*Fig. 1*): 1 guideline^1^, 4 meta-analyses^72,136–138^, 3 systematic reviews^139–141^ and 4 large database studies^142–145^.

#### Comparison of outcome in open groin hernia repair with and without antibiotic prophylaxis

##### Meta-analyses

Since January 2015 three new meta-analyses have been published on antibiotic prophylaxis (AP) in open inguinal hernia surgery^136–138^. The maximum number of included RCTs is 27 with a total of 8308 patients (*Table 1*).

The Cochrane analysis distinguished between open suture and mesh technique as well as between high infection and low infection risk environments^136^.

Studies with an SSI rate of ≥5 per cent are assigned to a high infection risk environment and those with an SSI rate of <5 per cent to a low infection risk environment.

For the five studies on suture repair with 1865 patients no significant difference was identified in the SSI rate for either the high infection risk environment (8.8 per cent with AP *versus* 8.9 per cent without AP; *P* = 0.97) or the low infection risk environment (1.6 per cent with AP *versus* 3.2 per cent without AP; *P* = 0.26).

Matters were different for the open mesh techniques with a total of 22 studies and 6443 patients.

Nine studies reported on a low infection risk environment and 13 studies on a high infection risk environment.

The nine studies conducted in a low infection risk environment reported an SSI rate of 2.6 per cent following open inguinal hernia mesh repair without antibiotic prophylaxis *versus* 1.8 per cent (*P* = 0.16) with antibiotic prophylaxis. The use of antibiotic prophylaxis for the open mesh technique in a low infection risk environment did not have a significant effect on the SSI rate^136^.

However, the 13 studies conducted in a high infection risk environment identified a significant influence of antibiotic prophylaxis on the SSI rate following the open mesh technique in that it was reduced from 8.5 per cent to 4.3 per cent (*P* = 0.0002).

As such, antibiotic prophylaxis should be administered for the open inguinal hernia mesh repair technique in a high infection risk environment. The evidence suggesting that antibiotic prophylaxis has no effect on the SSI rate in the open mesh technique in a low infection risk environment is classified by the Cochrane Collaboration as being of moderate quality.

Another meta-analysis^137^ investigated the influence of antibiotic prophylaxis on open mesh repair of groin hernias and included 16 RCTs with 5519 patients. Considering all RCTs, antibiotic prophylaxis significantly reduced the overall SSI incidence from 4.8 per cent to 3.2 per cent (OR 0.68, 95 per cent c.i. 0.51–0.91). However, after removal of two outlier studies, which were identified by evaluating the standard residual, the results of the meta-analysis became non-significant (OR 0.76, 95 per cent c.i. 0.56–1.02).

Another meta-analysis explored the efficacy of various antibiotics for prophylaxis of SSI following open inguinal hernia surgery^138^. Fifteen RCTs with 5159 patients were included. Ten of the 15 RCTs were from a high infection risk environment^137^. The meta-analysis showed that β-lactam/β-lactamase inhibitors and first-generation cephalosporins were significantly superior to placebo, with a pooled risk ratio of 0.44 (95 per cent c.i. 0.25–0.75) and 0.62 (95 per cent c.i. 0.42–0.92), respectively. If using antibiotics these are the family of antibiotics that are recommended^138^.

#### Current data on the SSI rates following open and laparo-endoscopic repair of groin hernias with no information on antibiotic prophylaxis

The Cochrane Collaboration meta-analysis reports an SSI rate of <5 per cent as constituting a low infection risk environment. As some of the studies included in the meta-analysis are older, this present publication aimed to collate more recent data on the SSI rates expected. No information was given in any of the studies on the use of antibiotic prophylaxis.

One meta-analysis with 12 RCTs and 1926 Lichtenstein and 2040 laparo-endoscopic inguinal hernia repairs compared the SSI rates for these two techniques^139^, identifying a significantly higher SSI rate following open Lichtenstein repair (1.7 per cent *versus* 0.95 per cent; *P* = 0.09).

An analysis of data from the Health Core Integrated Research database with 77 666 groin hernia repairs reported an SSI rate of 0.48 per cent for the open and 0.34 per cent for the laparo-endoscopic technique (*P* = 0.020)^139^.

Another analysis of data from the American College of Surgeons National Surgical Quality Improvement Program (ACS NSQIP) identified SSI rates of 0.51 per cent for 45 582 open and of 0.33 per cent for 17 919 laparo-endoscopic groin hernia repairs (*P* = 0.002)^140^.

A systematic review of the perioperative complications in inguinal hernia repair identified an overall complication rate of 2.9 per cent for 571 445 patients and an SSI rate of 0.48 per cent for 345 746 patients^141^. Another systematic review of SSIs after inguinal hernia repair performed in low and middle human development index countries identified for open groin hernia repair a rate of 4.1 per cent for open and of 0.4 per cent for laparo-endoscopic repair^143^.

#### Antibiotic prophylaxis in laparo-endoscopic groin hernia repair

For laparo-endoscopic inguinal hernia repair, HerniaSurge guidelines recommend no antibiotics in all patients and in any risk environment^1^.

The search did not yield any RCT studies. The best evidence was derived from a registry-based study.

The SSI rates presented demonstrate the beneficial effect of the laparo-endoscopic technique for the prevention of postoperative complications. All studies show lower SSI rates for the laparo-endoscopic technique compared with the open operation. SSI rates following the laparo-endoscopic technique have been consistently below 1 per cent, so it is not surprising that antibiotic prophylaxis did not confer any additional benefit. That was also confirmed by a multivariable analysis of data from the Herniamed registry for 48 201 patients^143^. No other potential influencing factors were identified^144^. Laparo-endoscopic groin hernia repair can be conducted independently of potential risk factors and in any risk environment without antibiotic prophylaxis.

#### Risk factors for SSIs in open inguinal hernia repair

Previous studies show a significantly higher SSI rate following open compared with laparo-endoscopic groin hernia repair. If one takes the threshold value of 5 per cent as per the guidelines of the Cochrane Collaboration, all studies are below the threshold including the open technique. The quality of groin hernia repair has improved worldwide as it is only being performed in a low infection risk environment. This suggests that based on the analysis by the Cochrane Collaboration, antibiotic prophylaxis is not required for groin hernia repair.

Another analysis of data from the ACS NSQIP with 57 951 patients with primary open inguinal hernia repair identified an SSI rate of 0.4 per cent^145^. A significantly higher SSI rate was reported for diabetes mellitus, BMI ≥ 35 kg/m^2^ and current smoking^145^.

The multivariable analysis of data from the Herniamed registry identified additional risk factors for SSI following open inguinal hernia repair: high ASA score (ASA IV *versus* I: OR 5.106, 95 per cent c.i. 1.836–14.200; *P* < 0.001), operation for recurrence (primary *versus* recurrent: OR = 0.512, 95 per cent c.i. 0.339–0.774; *P* = 0.001) and female sex (male *versus* female: OR = 0.532, 95 per cent c.i. 0.350–0.807; *P* = 0.003)^143^.

Therefore, in low infection risk environments, antibiotic prophylaxis should be considered for open inguinal hernia in these patient groups.

**Patients’ values and preferences inherent to Chapter 12**

Patients’ preferences are substantially concordant with panel recommendation direction and strength.

No relevant comments were added to the discussion.

*Summary*

In agreement with the HerniaSurge guidelines, in an environment with low risk for infection, antibiotic prophylaxis is not recommended in open or in laparo-endoscopic inguinal hernia surgery. The expert panel are aware that these recommendations are often not followed possibly for medico-legal and cultural factors. The recommendation that antibiotic prophylaxis is not recommended in elective laparo-endoscopic inguinal hernia repair in any patient and in any risk environment was downgraded to a suggestion (weak) with consensus of 80 per cent after only achieving a 64 per cent agreement when defined as strong. The argument was that antibiotics may still be appropriate in a small group of patients with certain risk factor profiles.

**Chapter 13. Anaesthesia in open inguinal hernia repair**

**Key Question 1: What is the preferred form of anaesthesia in open inguinal hernia repair in adults with primary unilateral hernias? General, spinal or local.**

**Key Question 2: What is the best anaesthesia in elderly and frail patients? Local, spinal or general.**

**Key Question 3: What is the best anaesthesia in the teaching/university hospitals?**

**Key Question 4: Is there evidence that short-acting lignocaine is safer than long-acting lignocaine in spinal (regional) anaesthesia?**

**Table:** Updated statements and recommendations

	Text	Level of evidence	Strength of recommendation
KQ 1–4			
Recommendation	Local anaesthesia is recommended for open repair of reducible inguinal hernias by surgeons/teams experienced with this technique.	☒☒☒☒	Strong
Recommendation	If performed correctly, local anaesthesia is a good alternative to general or regional anaesthesia in frail or co-morbid patients.	☒☒☐☐	Weak
Statement	Regional compared to general anaesthesia in patients aged 65 and older might be associated with a higher incidence of medical complications including myocardial infarction, pneumonia and venous thromboembolism.	☒☒☒☐	
Recommendation	General or local anaesthesia is suggested instead of regional in patients aged 65 and older.	☒☒☐☐	Weak
Statement	Open inguinal hernia repair under local anaesthesia can be safely performed by trainees under supervision of surgeons experienced in the administration of local anaesthesia.	☒☒☐☐	

**Key Question 1. What is the preferred form of anaesthesia in open inguinal hernia repair in adults with primary unilateral hernias? General, spinal or local.**

### Introduction

Open inguinal hernia repair can be performed under either local (LA), regional or general anaesthesia. Regional anaesthesia includes spinal, epidural and paravertebral routes. The ideal anaesthesia technique should provide good peri-/postoperative analgesia, have a low complication rate and be cost-effective. The HerniaSurge guidelines on groin hernia management recommended that local anaesthesia is preferred for open repair in reducible inguinal hernias, provided surgeons/teams are experienced in the technique. Paravertebral and epidural anaesthesia are not included in this chapter, due to limited studies on these anaesthetic techniques in inguinal hernia repair.

The HerniaSurge guidelines on groin hernia management demonstrated that local anaesthesia has several advantages over general or regional anaesthesia in elective reducible inguinal hernia repairs. When compared with general anaesthesia, local anaesthesia is more cost-effective when hospital and total healthcare costs are considered and provides earlier patient mobilization and earlier hospital discharge. A review article demonstrated lower urinary retention in local anaesthesia compared to spinal anaesthesia^146^. However, hernia registry data showed that local anaesthesia is associated with an increased risk of reoperation for recurrence in open inguinal hernia repair.

### Results

The literature review identified 75 articles (July 2015 to August 2020). After exclusion of RCTs already covered in the HerniaSurge guidelines, three RCTs^147–149^, two systematic reviews with meta-analysis^150,151^ and one network meta-analysis^152^ comparing different techniques in open anterior inguinal hernia repairs in adults were included in the analysis. The 2021–22 search for level 1 studies revealed one RCT of moderate quality that describes spinal *versus* general anaesthetic in TAPP inguinal hernia repair^153^.

The RCTs were scored according to SIGN as high quality. One RCT compared LA with spinal anaesthesia^149^, one RCT compared LA with general anaesthesia^147^ and one RCT analysed all three anaesthetic techniques^148^. The two RCTs comparing LA with spinal anaesthesia demonstrated that LA is effective, has good postoperative analgesia and fewer postoperative complications. The RCT by Rafiq *et al.*^147^ showed a shorter hospital stay in LA compared with general anaesthesia. When comparing all three anaesthetic techniques, patients receiving LA could be discharged faster^148^. These results confirm the findings of HerniaSurge and do not influence the statements and/or recommendations.

The systematic review by Prakash *et al.*^150^ evaluated LA compared to spinal anaesthesia in unilateral primary inguinal hernias. The new RCT from 2016^149^ was included in this systematic review in comparison to the publications used in HerniaSurge. Prakash *et al*. included 10 RCTs, with a total of 1379 patients. There was no significant difference in operative time between the two groups (*P* = 0.79). However, patients in the LA group reported significantly less pain (*P* < 0.01), lower rates of urinary retention (*P* < 0.01) and significantly increased satisfaction (*P* < 0.01). The conclusions confirm the findings of HerniaSurge and do not influence the statements and or recommendations.

The systematic review of Argo *et al.*^151^ evaluated all three anaesthetic techniques and included 18 RCTs. It was scored as high quality. The RCT by Zamani *et al.*^149^ was not included in this review. The overall complication rate and surgical time were similar in LA compared to the other anaesthetic techniques (*P* = 0.06 resp. *P* = 0.86). Urinary retention and operating room time were significantly decreased in LA (*P* = 0.0002 resp. *P* < 0.0001). Despite these advantages in favour of LA, patients under LA reported the same degree of satisfaction as the other anaesthetic techniques (*P* = 0.03). The literature review of the included studies showed a significantly decreased length of hospital stay and lower cost in the LA group. The conclusions confirm the findings of HerniaSurge and do not influence the statements and or recommendations.

A network meta-analysis by Olsen *et al.*^152^ included 53 studies (12 RCTs and 41 cohort studies), including 11 683 patients and rated as high quality. The aim of this study was to investigate possible differences in urinary retention and mortality after Lichtenstein repair under different types of anaesthesia. Urinary retention was seen in 0.1 per cent for LA, 8.6 per cent for regional anaesthesia and 1.4 per cent for general anaesthesia. The risk of urinary retention for regional anaesthesia had an odds ratio of 15.73 (*P* < 0.001) and for general anaesthesia an odds ratio of 4.07 (*P* = 0.04) compared with local anaesthesia. The mortality rate was zero in all three anaesthetic groups. These results strengthen the conclusions found in HerniaSurge and do not influence the statements and/or recommendations.

### Discussion

The RCTs, two systematic reviews and the network analysis were all scored as high quality with a low risk of bias. The results are very consistent, stating that local anaesthesia has advantages over general and regional anaesthesia. A consistent lower length of stay and lower cost is seen comparing LA *versus* either general or regional anaesthesia as well as a very significant lower risk of urinary retention in favour of LA. In some studies, LA offers less postoperative pain and higher patient satisfaction. Few studies had pain as the primary outcome. This last conclusion is biased by a lack of information on technique specifics, additional analgesia and/or sedatives during the operation and the exact definitions of pain. Complication rates are comparable between all techniques although two registry studies^154,155^ showed a possible higher risk of recurrence after LA.

When considering the advantages, it is recommended to perform open reducible primary inguinal hernia repair under local anaesthesia as first choice. There was expert consensus within HerniaSurge that operating under LA requires expertise (there is a learning curve to overcome) and experience. It is generally accepted that LA is particularly suitable for frailer or co-morbid patients.

**Key Question 2. What is the best anaesthesia in the elderly and frail patients? Local, spinal or general.**

### Introduction

The world’s elderly population is increasing; it has been estimated that by 2050 the number of elderly people will make up about 20 per cent of the world's population. The World Health Organization defines an elderly person as a patient over 65 years of age; however, this age limit is not universally recognized. Another limitation of the research has been the concept of frailty, because in the literature many of the studies regarding surgery in elderly and frail patients involve stratification of this population to assess operative risks. The HerniaSurge guidelines on groin hernia management report one registry study that found a higher incidence of medical complications in patients aged 65 years and older after regional anaesthesia (1.17 per cent) compared with general anaesthesia (0.59 per cent)^156^. The medical complications include myocardial infarction, pneumonia and venous thromboembolism. The recommendation is made that general or local anaesthesia is suggested over regional in patients aged 65 and older.

### Results

Covering the period from 2015 to July 2020, using the search terms above, only one article related to this key question has been identified. Faisal *et al*. performed a prospective study of 100 patients on the acceptability and outcome of operating on inguinal hernias among a population over 65 years of age who are at high risk for general or regional anaesthesia. Local anaesthesia was tolerated well in 95 per cent of the patients. Pain during the procedure was seen in 3 per cent of the patients and 1 per cent reported inguinodynia. No mortality was reported. The authors conclude that local anaesthesia is well tolerated and has favourable outcomes in elderly patients who are at high risk for general or regional anaesthesia.

### Discussion

High-quality medical evidence on the best anaesthetic technique in elderly and frail patients is lacking. Faisal’s study considered a single cohort of patients without stratification^157^. General anaesthesia seems to be associated with an increase in cognitive dysfunction in the elderly patient as well as an increased risk of developing Alzheimer’s disease^158^. Regional anaesthesia is less suitable due to a higher incidence of medical complications, and urinary retention in elderly patients. There is no evidence on health economics. Although the evidence is weak, general or local anaesthesia is suggested over regional anaesthesia in elderly and frail patients.

**Key Question 3. What is the best anaesthesia in the teaching hospital?**

### Introduction

Although the previous guidelines recommended the presence of an experienced supervisor for inguinal hernia surgeries performed under local anaesthesia by surgeons new to the technique, the HerniaSurge guidelines provide low evidence and make no statements or recommendations on the best anaesthetic technique for open inguinal hernia repair in the teaching hospital.

### Results

Covering the period from 2015, no articles were identified relating to the key question using the search terms.

### Discussion

As limited data are available on this topic, it is not possible to answer this key question. As a consequence, what has been outlined in the previous guidelines can still be considered valid. However, research is warranted to provide data on this topic. Therefore, we believe that the previous statement is still valid.

**Key Question 4. Is there evidence that short-acting is safer than long-acting lignocaine in spinal (regional) anaesthesia?**

### Introduction

The HerniaSurge guidelines on groin hernia management provided no evidence and made no statements or recommendations regarding the type of anaesthetic used during loco-regional anaesthesia. It was decided to review the literature regarding the use of short- or long-acting lignocaine in spinal anaesthesia for open inguinal hernia repair.

### Results

Covering the period from 2015, using the search terms, no articles relating to the key question were identified.

### Discussion

As limited data are available on this topic, it is not possible to answer this key question. More research is warranted to provide data on this topic. No recommendations were formulated.

**Patients’ values and preferences inherent to Chapter 13**

Patients’ preferences are substantially concordant with panel recommendation direction and strength.

Similarly to the choice of surgical technique, it is acknowledged that there are multiple options for anaesthesia, and these should be adapted to the hernia type and patient characteristics. Patients would choose the anaesthetic option that offers the best outcomes.

*Summary*

There is high-quality evidence with low risk of bias showing that local anaesthesia (LA) has advantages over general anaesthesia and especially over regional anaesthesia. A lower length of stay and lower costs are seen comparing LA with either general or regional anaesthesia. A significant lower risk of urinary retention is seen in favour of LA. In some studies, LA resulted in less postoperative pain and higher patient satisfaction. Few studies had pain as the primary outcome, so this conclusion is less strong. The experts agreed that performing inguinal hernia repair under local anaesthesia has a learning curve. The statement that when compared with general anaesthesia, regional anaesthesia in patients aged 65 and older is associated with a higher incidence of medical complications like myocardial infarction, pneumonia and venous thromboembolism received a low consensus of 68 per cent. With increasing patient frailty and co-morbidities, the experts agreed that there are benefits to using open repair under local anaesthesia and avoiding regional anaesthesia. However, the evidence is weak. The choice for local, regional or general anaesthesia in all patients should be tailored to minimize harm.

**Chapter 19. Chronic postoperative inguinal pain treatment**

**Key Question 1: What are the diagnostic modalities (including dermatome mapping, ultrasound (US), MRI (magnetic resonance imaging), CT (computed tomography) scan, infiltrations, nerve blocks) in the evaluation of postoperative chronic inguinal/scrotal/groin pain?**

**Key Question 2: What are the possible surgical therapeutic options (including neurectomy and (partial) mesh removal) in the treatment of postoperative chronic inguinal/scrotal/groin pain?**

**Key Question 3: What evidence is available on non-surgical therapeutic options (including role of centralization and multidisciplinary team approach) in the treatment of postoperative chronic inguinal/scrotal/groin pain?**

**Table:** Updated statements and recommendations

	Text	Level of evidence	Strength of recommendation
KQ			
Statement	In clinical practice, trigger point infiltrations and peripheral nerve blocks can be useful in the diagnostic management of chronic pain after inguinal hernia repair.	☒☒☐☐	
Statement	In patients with CPIP after laparoscopic preperitoneal inguinal hernia repair, MRI of the groin can be useful, mainly to exclude other pathologies.	☒☐☐☐	
Statement	For chronic neuropathic pain after open hernia repair, both open neurectomy and endoscopic retroperitoneal neurectomy provide acceptable outcomes.	☒☐☐☐	
Statement	Painful conditions interfering with sexual function after open hernia repair can also be improved by neurectomy, release of the spermatic cord and mesh removal.	☒☒☐☐	
Statement	In general, there is a risk of around 30 per cent that CPIP surgery will not be effective, with even a small risk for more pain.	☒☒☐☐	
Recommendation	It is recommended to inform patients clearly that evidence on the effectiveness of CPIP surgery is low and comes with a risk of pain intensification and other complications.	☒☒☐☐	Strong (upgraded)
Recommendation	A tailored approach to CPIP surgery (neurectomy, open mesh removal or combination) is suggested depending on the original repair method, experience of the surgeon, distribution and symptoms of pain, physical findings and potential radiographic images.	☒☒☐☐	Weak
Recommendation	It is suggested that microsurgical spermatic cord denervation is only performed in research settings.	☒☒☐☐	Weak
Statement	In clinical practice, peripheral nerve blocks can be useful in the therapeutic management of chronic pain after open inguinal hernia repair.	☒☒☐☐	
Statement	There is low evidence of the therapeutic value of repetitive trigger point infiltrations in CPIP after Lichtenstein repair.	☒☒☐☐	
Statement	No benefit has been shown for lidocaine and capsaicin patch for treatment of CPIP.	☒☒☐☐	
Statement	Pulsed radio frequency ablation may be an effective treatment for CPIP.	☒☐☐☐	
Statement	Early findings suggest that neuromodulation of the dorsal root ganglia (DRG) may be an effective treatment for chronic neuropathic pain conditions in the groin region.	☒☐☐☐	
Recommendation	The treatment of CPIP is complex. It is recommended to centralize CPIP evaluation and treatment in specialist centres with an experienced multidisciplinary team, depending on local settings.	☒☒☐☐	Strong (upgraded)
Recommendation	Pharmacologic and interventional measures—including therapeutic injection therapy—are suggested to continue for a minimum of 3 months (minimum of 6 months after hernia surgery).	☒☒☐☐	Weak

### Introduction

Chronic pain is a significant complication after inguinal hernia surgery leading to disability, dissatisfaction, and impaired productivity and quality of life. The international HerniaSurge guidelines for groin hernia management were published in 2018. They included a literature review until 1 January 2015^1^. Despite various interpretations of chronic pain, the HerniaSurge guidelines stated that chronic postoperative inguinal pain (CPIP) can be defined as ‘postoperative inguinal pain including a level of discomfort rated by the patient as at least “moderate” and impacting daily activities and lasting longer than a three-month time period’. While certain predisposing neuroanatomic and technical factors can be avoided, CPIP remains a complex challenge with several psychological, social, genetic and behavioural influences. In addition, it is important to determine whether the CPIP is indeed new postoperative pain (intensity, type, location) compared to preoperative pain status.

The previous HerniaSurge guidelines concluded that there is a paucity of evidence-based data on the management of CPIP. Therefore, the statements and recommendations were, respectively, (very) low evidence and weakly supported. As the guidelines needed an update, in June 2020 the HerniaSurge committee decided to review key chapters where recent publications could have an effect on the statements and/or recommendations. One of the topics distinguished by the committee was Chapter 19 on CPIP. The aim of this update is to review the most recent literature regarding CPIP and examine if previously included recommendations and statements are still valid. Additionally, the aim was to examine if there are new statements and recommendations that should be proposed in the light of new evidence in the literature. Publications from January 2015 until April 2021 were included. The original key questions were modified to three more logical new key questions.

**Key Question 1: What are the diagnostic modalities (including dermatome mapping, ultrasound (US), MRI (magnetic resonance imaging), CT (computed tomography) scan, infiltrations, nerve blocks) in the evaluation of postoperative chronic inguinal/scrotal/groin pain?**

### Introduction

When evaluating CPIP, it is important to perform an extensive history and physical examination. The use of an inguinal pain assessment form can be helpful to register these aspects in a standardized way. Traditionally, a distinction has been made between neuropathic and nociceptive pain due to, respectively, nerve damage or mesh interference as a cause of pain. However, it is unclear if and to what extent both pain patterns overlap and/or interfere. Therefore, it remains a question whether discrimination between both is clinically possible and useful. Still, dermatome mapping can be helpful to describe more objectively the superficial pain distribution and allows a comparison of both groins. It can also be used to document the evolution of skin sensitivity disturbances in the groin and potential peripheral and central pain sensitization^159^. After open repair, the ilioinguinal and iliohypogastric nerves are most at risk, whereas after laparoscopic repair, the genital branch of the genitofemoral nerve (and the lateral cutaneous nerve of the thigh) are more endangered. When patients present with so-called scrotal or testicular pain, it is important to differentiate between scrotal skin pain (which is often related to the genital branch of the genitofemoral nerve) *versus* scrotal content or true parenchymal testicular pain (orchialgia). The latter can be due to inherent testicular problems or due to involvement of the paravasal nerves in the spermatic cord^160^. In those circumstances, a formal urological evaluation is also warranted. Furthermore, it is key to obtain the original operation report(s) of the groin hernia operation(s) performed and of the different diagnostic and therapeutic actions that were already taken before. Information on interference of pain in sexual activity or work status and presence of other chronic pain problems (migraine, gynaecological, intestinal, back pain, hip, etc.) should also be documented clearly.

### Results

One randomized study has been performed to date. Due to the lack of high-level evidence, published cohort studies will be discussed here as well. An additional two articles were selected for the present update.

The study by Wijayasinghe *et al*. is a methodologically high-quality crossover study on US-guided local fascial plane trigger point infiltration (10 ml bupivacaine 0.25 per cent *versus* placebo) in 14 patients after open inguinal hernia repair (8 patients of the 22 randomized were excluded)^161^. Infiltrations were done around the spermatic cord and the authors also use the term ‘nerve block’. The median pain intensity decrease (primary endpoint) with bupivacaine was 63 per cent compared to 36 per cent with placebo (*P* = 0.003). However, there was no difference in movement-related pain, summed pain intensity scores, or sleep quality scores between the two groups; 10 of 14 patients had at least 50 per cent reduction in pain after local anaesthetic (LA) injection compared with 3 of 14 patients after placebo. There were no major complications. Follow-up was only 7–14 days. Because of the low number of patients this study provides only very low evidence for tender point blocks with bupivacaine.

A small retrospective study compared landmark-based (*n* = 20) *versus* US-guided (*n* = 16) ilioinguinal/iliohypogastric nerve blocks in patients with CPIP^162^. The number of patients that experienced at least 50 per cent pain reduction was comparable in both groups (70 and 79 per cent, respectively). There were no complications in both groups. No information was given on the duration of the effect.

Burgmann *et al*. performed a retrospective study in 53 patients on the comparison of MRI findings in patients with pain post-TEP (*n* = 55) *versus* pain-free post-TEP (*n* = 12) and unoperated (*n* = 39)^163^. Two experienced radiologists assessed the MRI examinations independently, according to a protocol. They concluded that for patients with post-TEP hernia groin pain, MRI is useful to confirm a correct flat mesh position and to identify possible (not operation-related) causes of groin pain. MRI revealed treatable findings explanatory for persisting groin pain in 15 per cent of the patients. However, MRI is of little help to identify a specific cause of groin repair related pain, as none of the predefined disorders on MRI (hernia, bulging, fibrosis, etc.) was observed significantly more in painful *versus* pain-free operated groins. Altogether, this study provides very low evidence for performing systematically an MRI in persistent pain after TEP.

### Discussion

This update of the HerniaSurge guidelines provides some new evidence on the diagnostic value of trigger point infiltrations. Because injections are minimally invasive and generally a safe procedure to perform, this might be an appropriate first diagnostic modality for CPIP after open inguinal hernia repair. Multiple authorities consider that peripheral nerve blocks serve an important diagnostic function and can be effective in the workup of CPIP. However, no evidence-based recommendations for the preferred technique of these blocks (US-guided, neuro-stimulator directed, anatomic landmark) could be made.

The literature does not always differentiate clearly between a peripheral nerve block or a trigger point infiltration. The fact that the latter also seems to be effective, at least temporarily, in a number of patients suggests that CPIP might be (partially) related to neuroma formation or chronic peripheral nerve inflammation. On the other hand, many trigger points are probably only inflammation/foreign body reaction/fibrosis with or without adjacent nerve or mesh, or even only referred pain points without actual anatomical abnormality. Future studies involving trigger point infiltration should be clearly differentiated from a peripheral nerve block, where the purpose is to block a specific (group of) nerve(s) proximal of the suggested nerve lesion. Also, a clear distinction should be made between US-guided *versus* landmark-based blocks, especially for peripheral ilioinguinal/iliohypogastric and spermatic cord blocks. Despite the fact none of the techniques for peripheral nerve blocks is superior, it is recommended that US-guided blocks are used in order to obtain optimal visualization of the injection site.

An infiltration at a trigger point or a nerve block may be beneficial for a certain time. To our knowledge, no objective criteria exist to determine whether an infiltration can be considered as effective. Moreover, different variables that might play a role in the size of the effect are the location and the amount and composition of the injection. In addition, some unmeasurable personal psychological and genetic factors are possibly involved. This also means that a certain placebo effect is likely, as demonstrated by Wijayasinghe *et al.*^161^, and ideally sham injection *versus* infiltrations with or without active substance injected should be compared in studies.

Concerning radiological investigations, it is logical to start with a dynamic US. No new qualitative data are available in this respect. The study by Burgmann *et al*. offers very low evidence to perform a systematic MRI after laparoscopic preperitoneal mesh placement^163^. Whether US would be an equally useful tool at much lower cost remains unclear. Anyhow, an expert radiologist and sufficient clinical input are needed for evaluating this difficult pathology.

**Key Question 2. What are the possible surgical therapeutic options (including neurectomy and (partial) mesh removal) in the treatment of postoperative chronic inguinal/scrotal/groin pain?**

### Introduction

Very low evidence suggested that both open and endoscopic retroperitoneal neurectomy provide acceptable outcomes for patients with CPIP. A tailored approach to such a neurectomy (with or without mesh removal) was suggested depending on the original repair method and presentation of complaints. It is logical from a pathophysiological point of view that neurectomy (as is also the case for nerve blocks) should be done proximal to the expected nerve lesion. The decision about neurectomy type—selective or triple—is debatable and at the moment left to the surgeon's discretion. There was insufficient evidence to support mesh removal alone without neurectomy. Also, painful conditions interfering with sexual function after open hernia repair can be improved by the same surgical interventions, including release of the spermatic cord (funiculolysis). The decision whether to opt for surgery (and the type of surgery) should be taken after multidisciplinary team evaluation including pain specialists. This was also highlighted in the previous HerniaSurge guidelines^1^.

### Results

From 2015 on, only one randomized study was published^164^. Verhagen *et al*. describe a non-blinded, single-centre study in which 54 patients with CPIP after primary Lichtenstein repair and at least 50 per cent pain reduction after a diagnostic trigger point infiltration were randomized to undergo an open (tailored) neurectomy of one or more inguinal nerves or trigger point infiltrations using LA. After 6 months, trigger point infiltration was successful in 22 per cent, but a neurectomy was successful in 71 per cent. Nineteen patients (76 per cent) crossed over from the infiltration to neurectomy group. Overall, the success rate of neurectomy was 65 per cent (*n* = 11/19). No major complications were observed, although pain worsened in one patient in the neurectomy group. The overall risk of bias was considered low, except the small numbers of patients and lack of blinding may have influenced outcomes of the study.

Other randomized studies have not been performed to date and thus firm conclusions regarding the effectivity of neurectomy or mesh removal cannot be drawn based on a high level of evidence. Due to the lack of high-level evidence, the published cohort studies will be discussed here as well. An additional 15 articles were selected for the present update.

Valvekens *et al*. reported on 15 patients operated for chronic postoperative groin pain. In three patients the original operation was not an inguinal hernia repair^165^. With a follow-up of one and a half years, only one in three patients benefitted from surgical intervention (with open neurectomy and/or mesh removal in half of the patients). The authors contemplate that most initial hernia repairs were laparo-endoscopic repairs in which the genitofemoral nerve can be injured, which was not adequately addressed at the reoperation.

Magnusson *et al*. published their experience on surgical treatment of CPIP in 2015^166^. Between 1999 and 2006, 111 patients who had undergone (more than one) surgery for persistent pain after previous groin hernia surgery were analysed. Open neurectomy was usually done in a selective manner after preoperative nerve blocks. Mostly the ilioinguinal nerve was treated. In 45 procedures the mesh was (partially) removed. In 14 cases a suture at the pubic tubercle was removed. Sixty-two per cent reported a decrease in pain, with a median follow-up of 3.6 years.

In 2016 Sun *et al*. concluded that most of their 44 patients did not experience immediate relief with reoperation for groin pain^167^. However, the majority (64 per cent patients) was (almost) pain-free at long-term follow-up (median 7 years). Mostly selective neurectomies were carried out (in 45 per cent) and the mesh was removed in 30 per cent. A recurrent hernia was repaired in more than half of the patients simultaneously.

Irrespective of the type of neurectomy (selected or triple) for CPIP, a laparo-endoscopic or open approach can be performed. Laparo-endoscopic surgery approaches the nerves in the retroperitoneal plane and thus dissects the nerve more proximal to its origin.

Most of the triple neurectomy data are derived from a single institute. Moore *et al*. described a prospective study of 62 patients undergoing laparo-endoscopic (totally extraperitoneal) retroperitoneal triple neurectomy (histopathologically confirmed) for (primary) neuropathic pain after laparoscopic (*n* = 26) or open (*n* = 36) repair^168^. Their results demonstrated complete pain relief in 21 per cent and a reduction of pain of Numerical Rating Scale (NRS) < 4 (scale from 0 to 10) in 81 per cent of the patients, average follow-up time of 1.9 years. Furthermore, the measured quality of life improved in 94 per cent. Thirty-two per cent of patients developed deafferentation hypersensitivity and in 31 per cent lateral abdominal wall laxity was found. In a subset of 10 patients that also underwent additional quantitative sensory testing, two interesting observations were made: duration of chronic pain showed a negative relationship to clinical outcome and two patients with complete pain relief had received multiple nerve blocks before surgery without meaningful effect^169^.

Moreno-Egea *et al*. described the results of 16 patients that underwent selective, double or triple transabdominal preperitoneal laparoscopic neurectomy for treatment of refractory inguinodynia (following a variety of groin repair techniques in 13/16 patients)^170^. About 69 per cent of patients were pain-free after surgery, with an average follow-up of 2 years.

Karampinis *et al*. evaluated a laparoscopic transabdominal retroperitoneal approach for double or triple neurectomy in nine patients, of which eight for CPIP after inguinal hernia repair^171^. Four patients were pain-free after neurectomy, three described an improved pain status, whereas two did not observe any pain reduction at 14 months of follow-up.

In a prospective, non-controlled explorative study by Pedersen *et al*., 66 of 240 referred patients received an open triple neurectomy with mesh removal in case of CPIP after Lichtenstein repair, or a laparoscopic triple neurectomy after previous endoscopic hernia repair^172^. Inclusion was based solely on clinical criteria. Patients were excluded for surgery if the index hernia repair was less than 6 months before, if a multimodal analgesic treatment had not been attempted for longer than 3 months, if a preoperative chronic pain syndrome unrelated to the inguinal intervention was present, or in case of non-compliance. Approximately 70 per cent experienced at least a 25 per cent improvement of pain-related functional impairment, with a median follow-up of 3 months.

Gangopadhyay *et al*. described 12 patients undergoing selective (*n* = 3) or triple (*n* = 9) open retroperitoneal neurectomy with intraoperative electrical nerve stimulation and proximal crush injury of the nerve (after open inguinal hernia repair in eight patients)^173^. Before surgery, there was a favourable response to diagnostic nerve blocks in all patients. Sixty-seven per cent of patients had partial/complete pain relief, but no information on duration of follow-up is available. Initially, a simultaneous peripheral nerve stimulator was placed simultaneously in three triple neurectomy patients, which has since been discontinued.

Three papers evaluated more specifically the role of mesh removal. Ramshaw *et al*. describe 94 consecutive operations (mesh removal with/without selective neurectomy) for CPIP after elective inguinal hernia repair in 93 patients^174^. Twenty-six laparoscopic (after previous laparoscopic repair) and 68 open and laparoscopic (after previous open repair) procedures were done. Other details were not mentioned. Forty-eight per cent of patients reported significant improvement, 41 per cent moderate, 11 per cent little or no improvement. There were 11 per cent of patients who developed recurrent hernias. Unfortunately, information on duration of follow-up was lacking.

Zwaans *et al*. performed a retrospective, non-comparative study of 74 consecutive patients undergoing open (partial or total) mesh removal with selective neurectomy (in 74 per cent) after Lichtenstein hernioplasty (median interval 35 months)^175^. A ‘meshoma’ was described in 31 per cent of patients. Sixty-four per cent had a successful outcome at a median follow-up of 18 months (success rate similar in both groups). Testicular atrophy was described in 2.7 per cent, and 7 per cent of patients developed a hernia recurrence.

The paper by Slooter *et al*. is also a retrospective, non-comparative study from the same group^176^. They report on 14 patients undergoing laparoscopic mesh removal after TEP/TAPP with large pore mesh (intentional partial resection (*n* = 2) and planned genitofemoral neurectomy (*n* = 2)). Exploration revealed no meshomas and only slight mesh folding in seven patients. There were no conversions or major intraoperative/early postoperative complications except from a small bladder laceration (*n* = 1). With a median follow up of 8 months, at least 50 per cent pain reduction was found in 64 per cent of patients. Patient satisfaction was excellent/good in 71 per cent of cases, although 14 per cent developed a recurrent hernia.

A multivariable analysis on the outcome of a retrospective cohort of 136 patients undergoing open surgery for CPIP after Lichtenstein repair suggests better outcome of surgery after spinal anaesthesia and worse outcome in female patients and patients using opioids^177^.

Regarding testicular pain, two publications describe the effect of microsurgical spermatic cord denervation (MSCD) if conservative therapies have failed. Marconi *et al*. report a prospective series of 50 patients (10 per cent CPIP) who were operated after a positive response to a spermatic cord block test and no response to a placebo injection^178^. With a follow-up of 6 months, 80 per cent of patients were pain-free. There were no intra- or postoperative testicular complications. Calixte *et al*. report a retrospective cohort of 772 patients (15 per cent CPIP), operated with a more targeted approach and robotic assistance, after temporary resolution of pain with a spermatic cord block^179^. Two testicular artery injuries were repaired during the same surgery without further consequences. Testicular ischaemia in another patient led to orchidectomy. In the CPIP group, pain had decreased by 25 per cent after 1 year.

### Discussion

A high level of expertise and experience are required for positive outcomes after surgery for CPIP. It is very difficult to evaluate data on the effect of mesh removal or neurectomy separately because the majority of studies combined mesh removal with a neurectomy or vice versa.

Neurectomy leads to improvement or even complete resolution of pain in the majority of patients. Selective neurectomy avoids unnecessary dissection of nerves and thus potentially diminishes the risk of deafferentation hypersensitivity, sensory disturbances (numbness), and potential motor deficits. On the other hand, as nerve anatomy is complex, varies vastly and the inguinal nerves can have interconnections, the risk of ongoing CPIP due to a wrong pre- or intraoperative estimation of the affected nerve is minimized by a triple neurectomy. After preperitoneal inguinal hernia repair, a retroperitoneal neurectomy is a more logical choice. The advantage of retroperitoneal neurectomy is that surgery is performed outside the field of scarring. However, this approach increases the risk of abdominal wall bulging (in particular after iliohypogastric and ilioinguinal motor denervation) and postoperative pseudohernias may occur. Previous studies have demonstrated a lateral abdominal wall laxity in up to 31 per cent^168^. In addition, the larger distribution of numbness may be bothersome for patients, especially in patients who did not experience pain in a particular numb area before neurectomy. Obvious disadvantages of open anterior neurectomy are that surgery is performed in a previously scarred surgical field, the highly variable inguinal neuroanatomy, and the inability to access nerves proximal to the site of damage after prior preperitoneal repair. Anyhow, the type (selective/triple) and approach (open/laparoscopic) of the neurectomy is probably a secondary consideration relative to the selection of appropriate patients that are likely to benefit from nerve resection.

With respect to MSCD, there is low evidence to suggest this as a last resort step for chronic orchialgia. The authors stress the importance of temporary pain resolution before surgery with a spermatic cord block. The latter seems a logical prerequisite, but the fact that a spermatic cord block also targets (largely) the genital branch of the genitofemoral nerve is an important confounding factor of concern.

Partial or total open mesh removal (with or without neurectomy) can be considered in CPIP due to mesh complications after Lichtenstein repair. Compression of adjacent structures like the spermatic cord and surrounding inflammation is thought to be the mechanism of this pain. Often the mesh is wrinkled and fibrotic, causing pain in certain positions like sitting. Laparoscopic mesh removal is a much more complex procedure with potential life-threatening complications. There is currently insufficient data to consider (partial) laparoscopic mesh removal.

Numerous important variables such as patient selection, previous treatments, surgeons’ experience, surgical technique, side effects, duration and type of follow-up (pain scores, questionnaires, neurologic examination techniques, etc.) are inconsistent in the literature and mostly unclear. Of note, outcomes are highly dependent on the definition of success. Therefore, heterogeneity in patient data, the small numbers of included patients in the individual reports and the retrospective character of most studies prohibits firm conclusions due to a high risk of bias. In the absence of a control group, it is very difficult to compare these data to the natural course of CPIP. Sham surgery would be ideal from a methodological perspective but raises ethical considerations. The importance of a better description of neuropathic pain by means of self-reported questionnaires like DN4^180^ and PainDETECT^181^, which report on pain sensitivity and neuropathic-like pain, remains unclear, as is the impact and optimal technique of preoperative peripheral nerve blocks.

**Key Question 3. What evidence is available on non-surgical therapeutic options (including role of centralization and multidisciplinary team approach) in the treatment of postoperative chronic inguinal/scrotal/groin pain?**

### Introduction

The HerniaSurge guidelines suggested with a weak recommendation to include a multidisciplinary team to manage chronic pain patients^1^. However, further specification on the definition and role of the different partners involved was not given. It is crucial that the team has experience with this pathology and that the important medical and paramedical disciplines are part of this team. Also, when a patient first presents to the pain specialist, we believe it is strongly advisable that he or she is seen at an early stage by an experienced surgeon in order to evaluate potentially important surgical aspects with relevance for the further work-up. A stepwise approach starting with minimally invasive measures like analgesics and nerve blocks was advocated. The HerniaSurge guidelines suggest that these should continue for a minimum of 3 months (minimum of 6 months after hernia surgery). Diagnostic/therapeutic nerve blocks or infiltration of trigger points can be done (blindly or US-guided) before a first multidisciplinary discussion. Although the HerniaSurge guidelines state that no benefit has been shown for lidocaine and capsaicin patch treatment of CPIP, it is not contraindicated to also try pharmacological therapy. More invasive management should ideally be done after evaluation by and discussion with the whole team. The HerniaSurge guidelines mentioned in this respect that pulsed radio frequency (RF) ablation may be an effective treatment and early findings suggested also that neuromodulation of the dorsal root ganglia (DRG) may be an effective treatment for chronic neuropathic pain conditions in the groin region^1^. Different algorithms have been suggested and published, but none of these is clearly based on high-level evidence.

### Results

Since 2015 there has been one randomized study^164^. This paper was discussed under the heading of KQ 2 because it compared open (tailored) neurectomy of one or more inguinal nerves with trigger point infiltrations using LA.

One retrospective cross-sectional study included 106 subjects with MR neurography-diagnosed groin pain (*n* = 41 after inguinal hernia repair), of which 58 subjects received CT-guided perineural injections based on abnormal inguinal nerve findings on MR (for example, hyperintensity or thickening of the respective nerves)^182^. Improvement was seen in 84 per cent of the cases. Although the results are promising, the retrospective, non-controlled, non-randomized study design and unclear duration of effect makes the study of too low quality to make a recommendation. Future research on this topic is warranted.

Another retrospective study included 10 patients (9 with CPIP) who underwent US-guided microwave ablation of inguinal nerves (mainly ilioinguinal nerve) after a positive response to diagnostic US-guided nerve block^183^. The results showed immediate pain reduction in 92 per cent of the patients. The average duration of clinically significant pain reduction was 10.5 months.

A study by Shaw *et al*. evaluated six patients undergoing peripheral nerve (field) stimulation after an externalized trial for the first week^184^. There were no major complications. Eighty-five per cent of patients were completely satisfied with an average follow-up of 22 months.

Again, no recommendations can be based on these two studies due to their retrospective, non-controlled, non-randomized study design with very small numbers of patients.

US-guided targeted cryoablation (UTC) of the peri-spermatic cord (branches of genitofemoral, ilioinguinal and inferior hypogastric nerves) has been described as a minimally invasive method for relieving scrotal content pain. During this procedure the targeted nerves are frozen with the intention to desensitize the nerves. Calixte *et al*. report on a retrospective series of 221 patients (15 per cent CPIP) as salvage treatment for patients who failed targeted MSCD. Although ‘minimally invasive’, the authors do not prefer to perform UTC first, because they believe that more scarring after ‘more aggressive’ UTC will make MSCD too challenging technically^185^. After a median follow-up of 3 years, success rates of 64 per cent with significant pain reduction (Pain Index Questionnaire-6) have been described for the whole cohort. No major complications were reported.

Injections with Botulinum-A toxin adjacent to the spermatic cord have been suggested for scrotal pain previously. In a study by Calixte *et al*. significant pain relief was obtained in 62.5 per cent of 44 patients at 7 months' follow-up^186^.

The problem of CPIP remains challenging due to its multidimensional aetiology. Multimodal treatment for CPIP has been suggested, such as with cognitive behavioural therapy (CBT)^187^. Although the CBT approach before CPIP surgery has been proven effective for selective patients, the study is methodologically heterogenic, resulting in difficulties identifying phenotypes with high success rate. On the other hand, the technique seems to have a minimal risk of adverse effect apart from time used.

### Discussion

LA injection therapy is minimally invasive and generally a safe procedure to perform. Repetitive trigger point infiltration is an early modality in a stepwise approach in the treatment of CPIP after Lichtenstein repair. Trigger point infiltration might prevent a number of patients from needing surgery, at least for 6 months. No data are available on the ideal interval of repetitive infiltrations to obtain a longer and/or more pronounced effect. However, if the outcome of repetitive trigger point infiltrations or repetitive blocks of a specific (group of) nerve(s) is comparable (or better), a causal factor at this site becomes highly likely. Therefore, therapeutic infiltrations serve as an important diagnostic tool.

Cryoablation and RF ablation have been the subject of a few case reports involving few patients and limited follow-up. Initial positive results should be viewed accordingly. All available studies on neuromodulation for CPIP cite sustained pain relief, quality-of-life improvement and/or analgesic use reduction or cessation. However, these studies have significant limitations, such as a retrospective design, case reports or series, lack of control groups, short follow-up times, and no report of adverse events or complications.

The importance of the subject and the paucity of evidence on this topic highlights the need for future high-quality research. Until more high-quality data emerge, it seems logical to propose a pragmatic treatment algorithm. In treating CPIP the repetitive effect of more proximal nerve blocks needs to be explored (transversus abdominis plane block, quadratus lumborum block, thoracolumbar block), if ‘standard’ peripheral nerve blocks or infiltration of trigger points are ineffective. Once the ideal level of optimal pain relief has been determined, more invasive techniques (cryoablation, RF, other neuromodulation) can be pursued if repetitive injections have insufficient long-term effect.

Accumulating evidence suggests that central sensitization is also driven by neuroinflammation in the peripheral and central nervous system (CNS). Due to the complexity of the pathology, treatment options, difficulties integrating a specific standardized stepwise therapeutic plan and need for an individualized approach, it is recommended that patients with CPIP should be managed in specialist centres.

**Patients’ values and preferences inherent to Chapter 19**

Patients’ preferences are substantially concordant with panel recommendation direction and strength.

Patients’ representatives agree that there is a need for specialized centres for the management of CPIP to manage this difficult condition that can severely impact patients’ quality of life.

*Summary*

In an extensive chapter developed by a larger team including two anaesthetists with an interest in chronic pain, 10 statements and 5 recommendations were formulated. There is only very low or low level evidence for the various modalities in the treatment of chronic postoperative inguinal pain (CPIP), in particular surgical interventions. This has led to the recommendation to inform patients clearly on the limited data on the effectiveness of CPIP surgery, with a potential risk of pain intensification and other complications with such interventions. A tailored approach to CPIP surgery (neurectomy, open mesh removal or combination) is suggested depending on the original repair method, experience of the surgeon, distribution and symptoms of pain, physical findings and potential radiographic images. The treatment of CPIP is complex and it is recommended to centralize the evaluation and treatment of CPIP in specialist centres with an experienced multidisciplinary team where possible.

**Chapter 21. Emergency groin hernia**

**Key Question 1: What is an acute groin hernia?**

**Key Question 2: What are the best management algorithm and the factors influencing the decision-making in the treatment of acute groin hernias?**

**Key Question 3: Which is the best surgical approach for acute groin hernias?**

**Table:** Updated statements and recommendations

	Text	Level of evidence	Strength of recommendation
KQ 1			
Statement	Acutely irreducible hernia—a hernia in which the contents cannot be reduced on physical exam but were previously reducible prior to the acute onset of symptoms.	☒☐☐☐	
Statement	Chronically irreducible hernia—a hernia in which the contents cannot be reduced on physical exam, which is of long standing and is not associated with sudden onset of new symptoms.	☒☐☐☐	
Statement	Strangulated hernia—hernia with strangulated content. Can only be described as such after the diagnosis is confirmed by preoperative imaging or intraoperative findings.	☒☐☐☐	
Recommendation	The term incarcerated hernia should be abandoned as not correctly describing the problem of acute hernias and substituted with aforementioned definitions.	☒☐☐☐	Strong (upgraded)
Recommendation	When managing a potential bowel ischaemia in an acutely symptomatic inguinal hernia it is suggested to use a combination of clinical symptoms together with biochemical parameters as the latter have a low specificity. Biochemical parameters of bowel ischaemia should not be used alone because of their poor specificity, but together with the clinical symptoms and signs can be utilized to aid the decisions around the management of acutely symptomatic groin hernias.	☒☒☐☐	Weak
Statement	Acutely irreducible groin hernia is a potentially life-threatening emergency situation and needs urgent surgical attention. The success of treatment depends on time from onset of symptoms to treatment and the bowel viability in the hernia sac.	☒☐☐☐	
Recommendation	Manual reduction is suggested to be attempted in all acutely irreducible hernias without suspicion of bowel ischaemia. After successful reduction, patients should undergo a period of observation until the analgesic/sedative drugs have worn off and the patient feels well enough to go home. If manual reduction is unsuccessful emergency surgery is indicated.	☒☐☐☐	Weak
Recommendation	Emergency surgery is recommended immediately when a suspicion of strangulation is made, or manual reduction was unsuccessful.	☒☐☐☐	Strong (upgraded)
KQ 2			
Recommendation	An algorithm is proposed to approach emergent cases.		
KQ 3			
Recommendation	When approaching an acutely irreducible groin hernia it is suggested to use diagnostic laparoscopy if expertise and resources are available and the patient's conditions allow it.	☒☐☐☐	Weak
Recommendation	A laparoscopic hernia repair can be attempted if expertise is available. However, a flat mesh repair for inguinal hernia individualized to the technique that gives best possible results in the centre where surgery is performed is recommended regardless of whether bowel strangulation is present or not.		
Recommendation	In patients with intestinal strangulation and/or concurrent bowel resection (clean-contaminated surgical field) a mesh repair could be used with a trend in favour of macroporous meshes.	☒☐☐☐	Weak

Acute irreducible hernias can have strangulated intestinal content and therefore need urgent surgical attention. This chapter will try to answer questions around the best definitions for common acute groin hernia events, and the best ways to investigate and treat acute irreducible hernias. Identification of the ischaemic content in an irreducible hernia is important. This chapter will describe known risk factors, biomarkers and investigations to help with decision-making on who needs an urgent operation and when to operate, including opinion on when surgery is futile.

All the recommendations reported in this chapter are based on very weak evidence. They should be introduced into clinical practice with a degree of caution.

**Key Question 1. What is an acute groin hernia?**

There is a limited amount of evidence in the available literature to support the currently widely used terms for describing acute groin hernias^188–192^. There is a lack of consistency among the authors in using terms such as irreducible, incarcerated and strangulated groin hernia. Previous HerniaSurge guidelines used the definition of *Incarceration*: inability to reduce the hernia mass into the abdomen and *Strangulation*: the blood supply to the herniated tissues is compromised^1^.

As the terms incarcerated, strangulated and irreducible are very ambiguous and often used interchangeably, the authors of this chapter decided to update these definitions.

There was consensus reached about changes to currently used nomenclature based on the observed lack of uniformity in hernia surgery.

The term incarcerated hernia should be abandoned as not correctly describing the problem of acute hernias. New terms to describe acute hernia occurrences should be used:

- *Acutely irreducible hernia*—a hernia in which the contents cannot be reduced on physical exam but were previously reducible prior to the acute onset of symptoms.
- *Chronically irreducible hernia*—a hernia in which the contents cannot be reduced on physical exam, which is of long standing and is not associated with sudden onset of new symptoms.
- *Strangulated hernia* **—**hernia with strangulated content. Can only be described as such after the diagnosis is confirmed by preoperative imaging or intraoperative findings.

**Key Question 2. What are the best management algorithm and the factors influencing the decision-making in the treatment of acute groin hernias?**

### Biochemical predictors of bowel resection/ischaemia in acutely symptomatic groin hernia patients

A number of studies focused on the presence of clinical (Systemic Inflammatory Response Syndrome – SIRS, long duration between symptoms and operation, high BMI, coronary heart disease, shock, pulmonary embolism, mesenteric arterial occlusion, organ failure), radiological (bowel loop dilatation, pneumatosis intestinalis, superior mesenteric vein thrombosis, free intraperitoneal fluid, portal vein thrombosis, splenic vein thrombosis) and biochemical (elevated serum lactate, acidosis, leucocytosis, haemoconcentration, hyperamylasaemia, elevated NLR (neutrophil-to-lymphocyte ratio), elevated PLR (prolactin)) markers of bowel ischaemia have been published in recent years^193^.

Time from the onset of symptoms more than 24 h, body temperature over 37°C, female sex, femoral type hernia^193^, age over 65 years, and signs of bowel obstruction^194^ are commonly mentioned predictors of bowel resection in patients with acutely symptomatic groin hernias and therefore poorer perioperative outcome^195^.

Focusing on biochemical markers and groin hernia patients specifically, a recent meta-analysis identified a raised white blood cell count and raised neutrophil count without any cut-off value as risk factors for bowel resection^195^. A review published by East *et al*.^191^ has also reported on serum levels of D-dimer above 300 ng/ml and serum phosphokinase levels of 140 IU/l and higher (compared to 90 in the control group) together with signs of bowel obstruction as good predictors of bowel ischaemia with relatively low specificity, but both sensitivity and negative predictive values over 90 per cent.

Several retrospective cohort studies have reported on the relationship between NLR and bowel resection ranging from 6.5 to 11.5 as a cut-off value^189,196,197^. The authors of the trial with the largest patient number out of these suggest an NLR of 6.5 as a good cut-off value especially when combined with signs of bowel obstruction, but also mention a total white blood cell count >8.5 and a neutrophil leukocyte count >7 to be good predictors of the need for bowel resection. The need to combine any of these markers with clinical symptoms and signs is evident because of the low specificity of these markers to indicate bowel ischaemia. For example, the NLR was over 6.5 in 80 per cent of the patients who required a bowel resection and in 50 per cent of the patients who did not^197^.

A patient with a strangulated bowel in an irreducible groin hernia often behaves differently to a patient with mesenteric ischaemia for other reasons. It is important to keep in mind that these people might not have the same symptoms and change in standard biochemical markers used to aid the diagnosis of bowel ischaemia in other scenarios. There is often not enough strangulated content to raise these markers, such as serum lactate, enough. Some studies have, however, shown more specific markers specific to strangulated tissue in groin hernias.

In a patient with an acutely symptomatic groin hernia, an NLR of 6.5 or greater (in combination with signs of bowel obstruction), and/or a D-dimer over 300 ng/ml, and/or a phosphokinase over 140 IU/l and/or prothrombin time over 13.5 s are potential biochemical markers of bowel ischaemia.

Time of onset of symptoms > 24 h, body temperature >37°C, signs of small bowel obstruction, female sex and age > 65 years are unfavourable influencing factors for emergency hernia repair perioperative outcomes.

Biochemical parameters of bowel ischaemia should not be used alone because of their poor specificity, but together with the clinical symptoms and signs can be utilized to aid the decisions around the management of acutely symptomatic groin hernias.

### When to safely attempt manual reduction—group of patients/symptoms/findings

There is a limited number of studies focusing on the safety of manual reduction in treatment of acute irreducible hernias. A systematic review by East and colleagues^191,193^ found that reduction can be successful in up to 70 per cent of patients presenting with a symptomatic irreducible inguinal hernia. The main factor associated with a reduced chance of successful reduction was the time from the onset of worsening pain in the groin. There is a linear relationship between the time of onset of symptoms to strangulation. The likelihood of strangulation and the necessity of bowel resection doubles for every 24 h from the onset of symptoms. Manual reduction is suggested in the acute setting providing contraindications, which include clinical features associated with strangulated hernia content such as red, painful skin overlying the hernia, are not present. Following successful manual reduction, a mesh repair in the elective setting is recommended. Definitive surgery to repair the hernia can be arranged for either the first elective list or delayed by weeks until surgery can be safely arranged. The main limitation of this comprehensive review is that it included very low to low-quality articles. The evidence and strength of recommendations coming from this study are weak.

A large retrospective cohort based on 13 028 patients with emergency admission and operation within 24 h included in the Herniamed registry between 2010 and 2019 was recently published^195^. It identified that the group of patients with successful manual reduction prior to surgical intervention had the lowest perioperative complication rates.

A retrospective cohort of 112 patients reported that elective surgery after reduction was significantly associated with a number of superior outcomes and a higher percentage of mesh repair^198^. Emergency surgery was found to be an independent risk factor for developing postoperative complications of grade II or higher.

There is only one study available that focuses on the algorithm for acutely irreducible groin hernias^199^. The review uses the most recent evidence to create a protocol for the use of manual reduction. The authors reached consensus to use the proposed algorithm on the topic of acute groin hernia presentation and the use of manual reduction in the first instance when there are no signs of bowel strangulation.

Acutely irreducible groin hernia is a potentially life-threatening emergency situation and needs urgent surgical attention. The success of treatment depends on time from onset of symptoms to treatment and the bowel viability in the hernia sac.

Manual reduction should be attempted in all acutely irreducible hernias without signs and risk factors of bowel strangulation. Following successful reduction patients should undergo a period of observation until the analgesic/sedative drugs have worn off and the patient feels well enough to go home. If manual reduction is unsuccessful, emergency surgery is indicated.

Patients should undergo emergency surgery immediately when a diagnosis of strangulation is made, or manual reduction was unsuccessful. (Strength of recommendation: strong.)

For a proposed treatment algorithm, see *Fig. 1*.

**Key Question 3. Which is the best surgical approach for acute groin hernias?**

This question has been addressed with previous guidelines KQ 21.e^1^ with the recommendation of a tailored approach. At the time of writing there was not enough evidence supporting an optimal surgical approach. In the last 5 years there were a number of papers published dedicated to the topic around the use of laparoscopy in emergency groin hernia surgery.

A prospective non-randomized trial^200^ comparatively analysed the surgical outcomes of 106 patients who underwent open (50.9 per cent) and laparoscopic repair (49.1 per cent) for acutely incarcerated/strangulated groin and obturator hernias. Hernia repair was performed through an open approach in patients seen from December 2000 to November 2011, whereas a laparoscopic TAPP or TEP approach was performed in patients seen from December 2011 to March 2017. Operative time was statistically significantly longer in the laparoscopic group (126.4 min *versus* 104.6 min, *P* = 0.0079), and postoperative length of hospital stay was longer in the open group (5.6 days *versus* 14.7 days, *P* = 0.0096). Patients in the laparoscopic group reported a lower incidence of postoperative complications (3.9 per cent *versus* 18.5 per cent, *P* = 0.0172). The study was low quality, mainly attributable to the non-randomized and the before/after study design that carries a high risk of selection/assignment bias.

A study of 94 patients with acutely incarcerated/strangulated inguinal hernias without contraindications for general anaesthesia, signs of peritonitis, definitive diagnosis of bowel perforation before surgery, and severe bowel distension preventing the use of a laparoscopic technique underwent TAPP repair^201^. Mean operating time was 61.6 ± 17.7 min and mean hospital stay was 3.9 ± 2.2 days. No patients were converted to open surgery and hernia reduction was successfully performed in all patients. The morbidity rate was 20.2 per cent. Nine (9.6 per cent) patients who were highly suspected to have had necrotic bowel avoided unnecessary bowel resections because the vitality of the incarcerated bowel recovered to normal after the TAPP procedure. No recurrence or infection was recorded during a mean follow-up period of 26.8 ± 9.8 months. Although it is limited by a single-centre retrospective cohort design, this study shows that TAPP appears to be safe and feasible for treatment of patients with acutely incarcerated/strangulated inguinal hernias when performed by experienced laparoscopic surgeons.

Liu *et al*. suggested that when approaching an irreducible groin hernia, a midline laparotomy should be avoided as much as possible. Conversely, a mesh repair through a preperitoneal approach is advisable^202^. The recent large registry study from Germany, including 13 028 patients with emergency admission and groin hernia repairs within 24 h, showed that the most commonly used technique was the Lichtenstein operation at 40.1 per cent, followed by TAPP at 29.7 per cent, TEP at 9.2 per cent and the Shouldice operation at 3.8 per cent. Looking at developments over the past 20 years, TAPP was used increasingly more often (21.9 per cent in 2013 *versus* 38.0 per cent in 2019; *P* < 0.001). In particular, in the case of the patients with emergency operation after reduction/taxis of the hernia sac contents, the proportion of TAPP repairs rose significantly from 25.8 to 45.6 per cent, whereas the proportion of Lichtenstein, Shouldice and ‘other techniques’ declined. The increase was consistent both among the emergency operations without bowel resection (30.6 per cent *versus* 37.4 per cent) and for the emergency operations with bowel resection (10 per cent *versus* 22.2 per cent)^195^.

When approaching an acutely irreducible groin hernia, a midline laparotomy should be avoided as much as possible due to the higher morbidity rate. When expertise is available, diagnostic laparoscopy may be a useful tool with the target of assessing bowel viability in all acutely irreducible groin hernias.

A laparoscopic hernia repair can be attempted if expertise is available. However, a flat mesh repair for inguinal hernia individualized to the technique that gives the best possible results in the centre where surgery is performed is recommended regardless of whether bowel strangulation is present or not.

### Mesh *versus* suture repair

Polypropylene mesh repair for acutely incarcerated groin hernia is associated with a decreased recurrence rate compared with non-mesh repair. Dai *et al*. found that polypropylene mesh repair for incarcerated groin hernia was associated with a decreased recurrence rate compared with non-mesh repair (2.3 per cent *versus* 19 per cent, *P* = 0.019), although mesh repair was not attempted in patients with bowel necrosis with/without perforation^203^. However, according to Bessa *et al*., the presence of non-viable intestine cannot be regarded as a contraindication for prosthetic repair, unless frank pus or faecal contamination is found in the hernia sac^204^. The results of the recent systematic review and meta-analysis by Ndong *et al*. suggest that the Desarda technique is a feasible and safe option in an emergency context with any particularly high rate of complications (considering the surgery in an emergency context) compared with mesh techniques^205^. Non-mesh repair for incarcerated or strangulated hernias could be considered a practicable option in low-resource settings.

In patients with intestinal strangulation and/or concurrent bowel resection (clean-contaminated surgical field) a polypropylene macroporous mesh repair is suggested.

**Patients' values and preferences inherent to Chapter 21**

Patients’ preferences are substantially concordant with panel recommendation direction and strength.

Patients' representatives acknowledge the importance and the relevance of the patient's safety requiring hernia repair in a possible life-threatening condition.

*Summary*

In this chapter the HerniaSurge guideline was significantly updated and improved. New definitions were proposed. It is suggested to introduce the following classification, avoiding the use of the term incarcerated hernia, which can also apply for some to a chronically irreducible hernia which can be asymptomatic:

- acutely irreducible hernia
- chronically irreducible hernia
- strangulated hernia.

The evidence for all key questions was low or very low. Weak recommendations include using a combination of risk factors for strangulation, clinical symptoms and biochemical markers to assist with a likely diagnosis of strangulation of the hernia contents, to facilitate timely treatment as necessary. Manual reduction can be attempted in an acutely painful irreducible hernia in the absence of symptoms or signs suggestive of strangulation of the hernia contents. If successful, after a period of observation (several hours), discharge with urgent elective surgery (if patient fit) is suggested, or same admission surgical repair preferably including a laparoscopic bowel exploration if resources and expertise are available. It is recommended to perform emergency surgery (within hours) when the diagnosis of a likely strangulated hernia is made (upgraded and obvious) and to consider a large pore mesh repair (upgraded) after bowel resection in a clean contaminated situation. An algorithm is proposed to aid medical professionals to assess such patients, make an early diagnosis and thus provide timely medical or surgical intervention as necessary.

**Chapter 28. Inguinal hernia surgery in low resource settings, type of mesh**

**Key Question: What is the value of non-commercial meshes in terms of safety (complications) and cost-effectiveness? Is there new evidence?**

**Table:** Updated statements and recommendations

	Text	Level of evidence	Strength of recommendation
KQ			
Statement	The use of low-cost mesh (with known chemical and physical characteristics) has had comparable results to commercial mesh in studies with 1 year follow-up.	☒☒☐☐	
Recommendation	When using a non-licensed low-cost mesh, outcome audits at a local level are recommended.	☒☐☐☐	Weak
Recommendation	When using a non-licensed low-cost mesh, it is recommended to be well informed of the type of mesh, origin and the safest method to sterilize it.	☒☐☐☐	Strong (upgraded)

### Results

The search yielded six relevant publications (two meta-analyses^206,207^, one RCT cost-effectiveness study^208^, two case series^209,210^ and one preclinical study^211^). The quality of the articles was scored using SIGN checklists by each author individually and where there was discrepancy a consensus agreement was reached with regard to quality. See PRISMA chart.

Since the publication of the HerniaSurge guidelines for groin hernia management there has been publication of one high-quality meta-analysis^206^ and one moderate-quality meta-analysis^207^. They all concluded that there is no significant difference in outcomes in the short term (1 year) between low-cost mesh and commercial mesh. The five RCTs in the high-quality meta-analysis had already been analysed in the original HerniaSurge chapter.

The conclusions are comparable to the recommendations published in the HerniaSurge guidelines.

In a high-quality cost-effectiveness analysis^208^ conducted on the same group of patients as those included in the RCT of Lofgren *et al*.^212^ (included in the original HerniaSurge guidelines), the cost difference resulting from the choice of mesh was $124⋅70 (€118⋅10), although the cost of the commercial mesh ($125) was higher than would be expected. In the low-cost mesh group, the costs per disability-adjusted life-years (DALY) averted and quality-adjusted life-years (QALY) gained were $16⋅80 (€15⋅90) and $7⋅60 (€7⋅20), respectively. The corresponding costs were $58⋅20 (€55⋅10) and $33⋅30 (€31⋅50) in the commercial mesh group. A sensitivity analysis was undertaken including cost variations and different health outcome scenarios. The maximum costs per DALY averted and QALY gained were $148⋅40 (€140⋅50) and $84⋅70 (€80⋅20), respectively.

In a preclinical study^211^ analysing the effect of sterilization on the mechanical structure of nine different mosquito net meshes, the authors reported that the reduction of the mosquito net surface area by more than 40 per cent due to sterilization at 121°C resulted in a loss of macroporous structure, turning the mesh into a hard, shrunken, non-pliable mass. Sterilization at 134°C caused some mosquito nets to melt, completely destroying their porous structure. In addition, there remains a lack of evidence about the quality control of the polymer matrix and the efficacy of low-cost mesh in the long term, where mesh shrinkage or degradation may occur resulting in recurrence. In an article concerning quality of non-commercial mesh and sterilization strategies large differences were seen after different methods of sterilization of 10 different types of mesh. Non-commercial mesh has a risk of melting and shrinking when incorrectly sterilized. The clinical consequences are unknown, but caution is advised. Quite often low resource hospitals only have autoclave sterilization machines, and the temperature will often be too high. The quality of the different types of non-commercial mesh especially after sterilization remains a concern.

The two case series only indicated that the use of low-cost mesh is feasible and seems safe with good results in a short-term analysis by Rouet *et al.* in Cameroon^209^ and Yenli *et al*. in Ghana^210^.

**Patients' values and preferences inherent to Chapter 28**

Patients’ preferences from low- and middle-income countries (LMIC) are lacking because patients’ representatives from those areas could not be interviewed for logistical reasons.

*Summary*

Low-cost mesh appears to be safe when compared to commercial mesh in the short term as long as the polymer matrix is known and has been tested to ensure there are no harmful chemical additives or contaminants left after cleaning and sterilization of the material. The best low-cost polymer matrix and sterilization technique is not clear but is critical for safe use. When using a non-licensed low-cost mesh, it is recommended to be well informed of the type of mesh, its origin, chemical properties and a safe method to sterilize it prior to use.

#### Robotic surgery in inguinal hernia

The application of the robotic approach in inguinal hernia repair was not addressed in the present guideline as the steering committee concluded that this technology is too early in its implementation. The robot could play an important role in complex cases where higher dexterity and enhanced view could represent the ideal tool to address those scenarios where standard minimally invasive surgery is limited^213^. Currently, evidence showing promising results for this approach exists^214^; nevertheless, the high costs and low penetration in clinical practice still limit the production of high-level studies that could form the basis of robust recommendations. There are limitations such as sustainability and uptake is mainly by specialized surgeons for standard operations^215,216^.

#### Future of guidelines development

The EHS has a large team working on guideline development.

A dedicated group will evaluate the prioritization of other KQs that were left unevaluated in this update. The methodological approach will likely be different, using the latest techniques, and the KQs themselves may change. As a consequence, some chapters were left unchanged due to the scarcity of new papers and the possibility of being changed completely.

Guideline methodology is constantly evolving and improving to appraise evidence and create recommendations. The methodology chosen for this process and for the future is the Grading of Recommendations, Assessment, Development, and Evaluations (GRADE)^217^. GRADE is very different in comparison to the traditional model adopted in surgical guidelines preparations and is currently becoming the international standard adopted by many scientific associations. New methods need a transition period to be adapted to the unique aspects of surgical research. The EHS has currently tested and used this approach in the parastomal hernia guidelines, the updated KQ on parastomal hernia prevention with mesh, and for the incisional hernia guidelines that are currently being developed. Cochrane experts have been involved to define search strategy and retrieval of publications, as well as guide the group in the difficult methodological choices in the process.

The hernia specialists gathered in a panel of experts have shared and selected publications online with a dedicated platform (Rayyan)^218^. Data extraction from the articles is also performed by Cochrane analysts creating tables of evidence and performing statistical analysis and graphs to synthesize data.

The final presentation of the evidence and voting on the statements and recommendations was done during several expert meetings. An independent chair and co-chair facilitated the discussion and aimed to avoid the influence of strong opinions.

During the writing process of previous guidelines, it was concluded that developing guidelines in the traditional EHS manner was very time-consuming. Not all surgeons are experts in guideline development and perform the work on a voluntary basis in their own time with many conflicting commitments. The workflow is at continuous risk as guidelines are reaching the expiry date too soon after publication. Moreover, the preparation of a single key question requires skills that are not evenly distributed among general surgeons ranging from software management (Rayyan, Excel, revMAn, GRADE pro, GDT), advanced statistical knowledge, and familiarity with new appraisal methodology. Therefore, the importance of embarking in our future initiatives requires professionals specialized in guideline methodology. In our opinion the GRADE approach will give the EHS the opportunity to adopt a system that is clear and not only focused on evidence but also on feasibility, patients' perspectives and the potential impact on health systems following guideline publication. The opportunity to give recommendations that are clear, unequivocal and balanced among the perspective of the different stakeholders is something needed and eagerly asked for from abdominal wall specialists. Consensus meetings and Delphi panels can increase/decrease the level of recommendations where indicated, adding specific expertise and collective knowledge to the often low level of evidence available. Input and active participation of expert hernia surgeons in developing guidelines is a *sine qua non*. The main vision we share is to create dynamic guidelines that are up to date and easily accessible for the users. Naturally they should be evidence-based and comply with the current standards of quality (AGREE II domains).

The EHS Board wants to create a working group under the guidance of the secretary for science that will be tasked with forming small teams responsible for single Key Questions (KQs) production. They will update on all Hernia topics in a modular fashion where topics are considered according to a time limit criterion, associated with surveys and dedicated instruments for KQ prioritization^6,7^. On a continuous basis these KQ teams will have access to the most recent publications by Cochrane professionals. These articles are appraised, and evidence tables and recommendations prepared in a GradeProGDT environment. The teams are expected to stay active for longer periods but the bulk of the tasks to accomplish this will be focused on limited literature appraisal and prepared with the help of professional methodologists and statisticians. The team members will have to be diverse in all aspects such as training expertise, surgical expertise, European geographical regions and groups of stakeholders.

#### Dissemination and assessing impact

The impact of the present updated guidelines on the clinical community will be assessed through online surveys sent to EHS members 2 years after publication to allow for a sufficient period of dissemination and uptake. EHS has requested each affiliated national chapter to produce a native language version of this paper to help the dissemination process.

All the process of development has been shared during EHS meetings in Manchester and Barcelona and voted with >75 per cent of concordance on statements and recommendations. Conferences for the guideline's dissemination have also been planned through the help of national chapters all across Europe. EHS has a section on the website dedicated to Guidelines as well as open discussions on social media.

Similarly to initiatives with Primary Ventral Hernia Guidelines, patients will also be provided with a plain-language summary of the present document (translated in local languages) to help them and their clinicians in the shared decision process regarding the best treatment for their condition.

We will need a large number of surgeons, young researchers and patients' representatives to accomplish this ambitious mission. Conflicts of interest will have to be monitored and all funds for the costs will need to be transparently communicated to assure our readers on the impartiality and quality of our analyses and recommendations.

## Supplementary Material

zrad080_Supplementary_Data

## Data Availability

All data used in the present work are presented in Supplementary material.
