# Corrigendum to: Update of the international HerniaSurge guidelines for groin hernia management

**DOI:** 10.1093/bjsopen/zrae034

**Published:** 2024-03-14

This is a corrigendum to: Cesare Stabilini, Nadine van Veenendaal, Eske Aasvang, Ferdinando Agresta, Theo Aufenacker, Frederik Berrevoet, Ine Burgmans, David Chen, Andrew de Beaux, Barbora East, Jose Garcia-Alamino, Nadia Henriksen, Ferdinand Köckerling, Jan Kukleta, Maarten Loos, Manuel Lopez-Cano, Ralph Lorenz, Marc Miserez, Agneta Montgomery, Salvador Morales-Conde, Chris Oppong, Maciej Pawlak, Mauro Podda, Wolfgang Reinpold, David Sanders, Alberto Sartori, Hanh Minh Tran, Mireia Verdaguer, Reiko Wiessner, Michael Yeboah, Willem Zwaans, Maarten Simons, Update of the international HerniaSurge guidelines for groin hernia management, *BJS Open*, Volume 7, Issue 5, October 2023, zrad080, https://doi.org/10.1093/bjsopen/zrad080

In the originally published version of this article, the supplementary data file was inadvertently omitted.

This has been corrected.

