# Supplementary material for: Update of the international HerniaSurge guidelines for groin hernia management
Source: BJS Open. 2023 Oct 20;7(5):zrad080. doi: 10.1093/bjsopen/zrad080 (PMC10588975; doi:10.1093/bjsopen/zrad080)
Supplement: zrad080_Supplementary_Data [file zrad080_supplementary_data.docx]

Update of the International HerniaSurge Guidelines for groin hernia management.

Cesare Stabilini University of Genoa, Department of Surgery (DISC), Genoa, Italy

Nadine van Veenendaal University of Groningen, University Medical Center Groningen, Department of Anesthesiology, the Netherlands

Eske Aasvang Department of Anaesthesiology, the Center for Cancer and Organ Diseases, Copenhagen University Hospital Rigshospitalet, Copenhagen, Denmark

Department of Clinical Medicine, University of Copenhagen, Copenhagen, Denmark

Ferdinando Agresta Vittorio Veneto General Hospital, Vittorio Veneto, Italy

Theo Aufenacker Rijnstate Hospital, Arnhem, Netherlands

Frederik Berrevoet University Hospital Ghent, Ghent, Belgium

Ine Burgmans University Medical Center Utrecht, Utrecht, Netherlands

David Chen David Geffen School of Medicine at UCLA, Los Angeles, United States

Andrew de Beaux Royal Infirmary of Edinburgh, Edinburgh, United Kingdom

Barbora East Fakultní Nemocnice v Motole, Prague, Czech Republic

Jose Garcia-Alamino Universitat Ramon Llull, Barcelona, Spain

Nadia Henriksen Department of Gastrointestinal and Hepatic Diseases, Copenhagen University Hospital–Herlev and Gentofte, Herlev, Denmark

Ferdinand Köckerling Vivantes Hospital Berlin, Academic Teaching Hospital of Charite´ University Medicine, Berlin, Germany

Jan Kukleta Klinik Im Park, Zurich, Zurich, Switzerland

Maarten Loos SolviMáx Centre of Excellence for Abdominal Wall and Groin Pain, Eindhoven, the Netherlands.

Department of General Surgery, Máxima Medical Center, Veldhoven, the Netherlands

Manuel Lopez-Cano Hospital Universitari Vall d'Hebron, Barcelona, Spain

Ralph Lorenz Hernia Center 3+CHIRURGEN, Berlin, Germany

Marc Miserez KU Leuven– University Hospital Leuven, 3000 Leuven, Belgium

Agneta Montgomery Skåne University Hospital, Malmö, Sweden

Salvador Morales-Conde Universidad de Sevilla, Sevilla, Spain

Chris Oppong Derriford Hospital Plymouth, Plymouth, UK

Maciej Pawlak North Devon Comprehensive Hernia Centre, North Devon District Hospital, Royal Devon University Healthcare NHS Foundation Trust, Barnstaple, UK

Mauro Podda Azienda Ospedaliero Universitaria di Cagliari, Cagliari, Italy

Wolfgang Reinpold Gross-Sand Hospital Hamburg, Hamburg, Germany

David Sanders North Devon Comprehensive Hernia Centre, North Devon District Hospital, Royal Devon University Healthcare NHS Foundation Trust, Barnstaple, UK

Alberto Sartori Ospedale Civile di Montebelluna Montebelluna, Italy

Hanh Minh Tran Westmead Clinical School, Sydney Medical School, University of Sydney, Australia

Mireia Verdaguer Hospital Universitari Vall d'Hebron, Barcelona, Spain

Reiko Wiessner Bodden-Kliniken Ribnitz-Damgarten GmbH, Ribnitz-Damgarten, Germany

Michael Yeboah Department of Surgery, School of Medical Sciences, Kwame Nkrumah University of Science and Technology, P.M.B, Kumasi, Ghana, West Africa

Willem Zwaans SolviMáx Centre of Excellence for Abdominal Wall and Groin Pain, Eindhoven, the Netherlands.

Department of General Surgery, Máxima Medical Center, Veldhoven, the Netherlands

Maarten Simons Department of Surgery, Onze Lieve Vrouwe Gasthuis Hospital, Amsterdam, the Netherlands.

Corresponding Author:

Cesare Stabilini, MD, PhD

Policlinico San Martino IRCCS

Largo R. Benzi 10, 16132

Genoa, Italy

Email: [cesarestabil@hotmail.com](mailto:cesarestabil@hotmail.com)

**Supplementary Materials - Index**

| **Supplementary Methods** |  |
| --- | --- |
| Search strategy for Herniasurge update | *pag. 2* |
| **Supplementary Figures and Tables** |  |
| PRISMA chart for herniasurge update | *pag. 7* |
| Table with selected articles | *pag. 16* |

**Supplementary Methods**

**Search strategy and PICO questions**

**6a. Tissue repair**

Search terms: “Inguinal hernia”, “pure Tissue repair“, “Shouldice repair“, “Desarda repair“, “Bassini repair“,“ Moloney-Darn repair“, “McVay repair”, “Marcy Repair“

PICO Question: Is there new evidence concerning mesh versus non-mesh repair for inguinal hernias?

PATIENT: Adult patients who have had a pure tissue inguinal hernia repair without mesh

INTERVENTION: open inguinal hernia repair without mesh

COMPARISON: open and endoscopic inguinal hernia repair with mesh

OUTCOME: recurrence, chronic pain, complications, QoL, operating time

PICO Question: Is there new evidence concerning best non-mesh technique for inguinal hernias?

PATIENT: Adult patients who have had a pure tissue inguinal hernia repair without mesh

INTERVENTION: Open inguinal hernia repair without mesh in a comparison technique (Desarda, Moloney-Darn, Bassini, Marcy, McVay)

COMPARISON: Open inguinal hernia repair without mesh in a established technique (Shouldice)

OUTCOME: recurrence, chronic pain, complications, QoL, operating time

**6d. Pre-peritoneal repair**

**KQ: Is there new evidence concerning open posterior (preperitoneal) versus open anterior repair (Lichtenstein) for inguinal hernias?**

**PICO:**

Patient: Adult patients who have had a preperitoneal inguinal hernia repair with mesh

Intervention: open posterior inguinal hernia repair with mesh

Comparison: open anterior inguinal hernia repair (Lichtenstein)

Outcome: recurrence, chronic pain, complications, QoL, operating time

Search terms: (((((((((Hernia, Inguinal[MeSH Terms]) OR (Hernia, Femoral[MeSH Terms])) OR ("Inguinal Hernia"[Title/Abstract])) OR ("Inguinal Hernias"[Title/Abstract])) OR ("Femoral Hernia"[Title/Abstract])) OR ("Femoral Hernias"[Title/Abstract])) OR (Herniorrhaphy[MeSH Terms])) OR ("Open hernia repair"[Title/Abstract])) OR ("groin hernia repair"[Title/Abstract])) OR ("repair of an inguinal hernia"[Title/Abstract]).

(((((((((((((((((("preperitoneal"[Title/Abstract]) OR ("transinguinal preperitoneal"[Title/Abstract])) OR ("posterior repair"[Title/Abstract])) OR ("posterior approach"[Title/Abstract])) OR (Rives[Title/Abstract])) OR (Wantz[Title/Abstract])) OR ("Kugel"[Title/Abstract])) OR (Grid-Iron[Title/Abstract])) OR (Trep[Title/Abstract])) OR ("TIPP"[Title/Abstract])) OR ("Transinguinal Preperitoneal Technique"[Title/Abstract])) OR ("Modified Kugel"[Title/Abstract])) OR ("Stoppa Repair"[Title/Abstract])) OR ("GPRVS"[Title/Abstract])) OR ("Giant Prostetic Reinforcement of the Visceral Sac"[Title/Abstract])) OR ("READ"[Title/Abstract] AND "Hernia"[Title/Abstract])) OR ("Ugahary"[Title/Abstract])) OR ("Preperitoneal gridiron hernia repair"[Title/Abstract])) OR ("horton/florence"[Title/Abstract]). Limits: From 2015-june 2020.

**Key question:**

**Is there new evidence concerning open posterior (preperitoneal) versus laparo-endoscopic repair (TAPP or TEP)for inguinal hernias?**

**PICO:**

Patient: Adult patients who have had a preperitoneal inguinal hernia repair with mesh

Intervention: open posterior inguinal hernia repair with mesh

Comparison: laparo-endoscopic inguinal hernia repair (TAPP or TEP)

Outcome: recurrence, chronic pain, complications, QoL, operating time

Search terms: (((((((((Hernia, Inguinal[MeSH Terms]) OR (Hernia, Femoral[MeSH Terms])) OR ("Inguinal Hernia"[Title/Abstract])) OR ("Inguinal Hernias"[Title/Abstract])) OR ("Femoral Hernia"[Title/Abstract])) OR ("Femoral Hernias"[Title/Abstract])) OR (Herniorrhaphy[MeSH Terms])) OR ("Open hernia repair"[Title/Abstract])) OR ("groin hernia repair"[Title/Abstract])) OR ("repair of an inguinal hernia"[Title/Abstract]).

((((((Laparoscopy[MeSH Terms]) OR (Laparoscopes[MeSH Terms])) OR ("tapp"[Title/Abstract])) OR ("transabdominal preperitoneal approach"[Title/Abstract])) OR ("tep"[Title/Abstract])) OR ("totally extraperitoneal patch plasty"[Title/Abstract])) OR ("enhanced-view totally extraperitoneal technique"[Title/Abstract]).

**6f. Endo-laparoscopic repair**

*PICO:*

| PATIENT: Adult patients with primary unilateral inguinal hernia  INTERVENTION: Open anterior inguinal hernia repair (Lichtenstein technique)  COMPARISON: Laparo-endoscopic inguinal hernia repair (TEP or TAPP technique)  OUTCOME: Recurrence, pain, learning curve, postoperative recovery, costs |
| --- |

*Search strategy:* (Hernia, inguinal) OR (Inguinal hernia) OR (groin hernia) AND (TEP) OR (total extraperitoneal laparoscopic repair) OR (TAPP) OR (transabdominal preperitoneal laparoscopic repair) OR (Lichtenstein) OR (open anterior hernia repair) OR (laparo-endoscopic hernia repair). Limits: Humans, from January 2015 - May 2021 (included).

**10. Mesh**

**PICO – KQ1 open (Lichtenstein)**

Patient: Patient with an uncomplicated unilateral primary inguinal hernia treated with open anterior mesh repair (Lichtenstein)

Intervention: Inguinal hernia repair using LWM

Comparison: Inguinal hernia repair using HWM

Outcomes: Pain >3 months p.o.; any pain, recurrence, foreign body feeling.

**Search terms**

The following search terms were used: “inguinal hernia”, “groin hernia”, “open hernia repair”, “open mesh repair”, “lightweight mesh”, “highweight mesh”, “heavyweight mesh”, “general anesthesia”, “treatment”. Only publications within the timeframe of January 2015 - July 1, 2020 for level one evidence and large cohort studies were included.

**PICO KQ2– laparo-endoscopic**

Patient: Patient with an uncomplicated unilateral primary inguinal hernia treated with laparo-endoscopic mesh repair (TAPP/TEP)

Intervention: Inguinal hernia repair using LWM

Comparison: Inguinal hernia repair using HWM

Outcomes: Pain >3 months p.o., any pain, recurrence, foreign body feeling

**12. Antibiotic prophylaxis**

**PICO**

Patient: Patient with an inguinal hernia repair (open and laparo-endoscopic)

Intervention: Prophylactic antibiotics

Comparison: No prophylactic antibiotics

Outcome: Complications, wound infection, costs

**Search terms:**
(Inguinal hernia) AND (Antibiotic prophylaxis), (groin hernia) AND (Antibiotic prophylaxis), (Hernia) AND (Antibiotic prophylaxis).
Limits: Humans, January 2015 – November 2021

**13. Anesthesia**

KQ1

**PICO**

**P**atient: patient with a reducible unilateral primary inguinal hernia

**I**ntervention: inguinal hernia repair under local

**C**omparison: inguinal hernia repair under regional and or under general anesthesia

**O**utcome: complications, length of stay, recurrence, QoL, urinary retention, mobilization, acute and chronic pain

**Search terms**

The following search terms were used: “inguinal hernia”, “groin hernia”, “open hernia repair”, “open mesh repair”, “local anesthesia”, “spinal anesthesia”, “general anesthesia”, “treatment”. Only publications within the timeframe of January 2015- July 1, 2020 for level one evidence including large cohort studies were included.

KQ 2

**PICO**

**P**atient: elderly and frailty patients with groin hernia 65 years

**I**ntervention: local anesthesia and possible combinations adding pain management or PSA

**C**omparison: spinal (regional) or general anesthesia or Tap-block or other combined techniques

**O**utcome: complications, Length of stay, costs, mortality, urinary retention

**Search terms**

The following search terms were used: “inguinal hernia”, “groin hernia”, “open hernia repair”, “open mesh repair”, “local anesthesia”, “spinal anesthesia”, “general anesthesia”, “treatment”, “elder and frail patients”.

KQ3

**PICO:**

**P**atients: hernia in teaching hospital

**I**ntervention: local anesthesia

**C**omparator: spinal (regional) or general or Tap-block or other combined techniques?

**O**utcomes: length of stay, complications, mortality, morbidity, recurrences, urinary retention

**Search terms**

The following search terms were used: “groin hernia”, “inguinal hernia”, “teaching hospital”, “anesthesia”.

KQ 4

**PICO**

**P**atients: patients with hernia

**I**ntervention: short acting lido and all types (Bupi)

**C**omparator: longacting lido and all types

**O**utcomes: urinary retention, length of stay, QoL

**Search terms**

The following search terms were used: “inguinal hernia”, “groin hernia”, “long acting lidocaine”, “short acting lidocaine”, “anesthesia”.

**21. Emergency**

Search strategies and results:

Two independent searches were done on 5/10/2020 and 12/10/2020 by two independent guideline collaborators.

Search limitations: time of publication - 01.01.2015 and 05/06/2021

An additional reference cross check and literature search has been performed on 5^th^ of June 2021 before completion of all full text reviews to verify if any new articles of relevance had been missed.

385 articles were screened form the first search, 68 articles were selected after excluding irrelevant

articles.

((inguinal hernia Title/Abstract) OR (femoral hernia Title/Abstract ) OR (groin herniaTitle/Abstract)) AND ((incarcerated Title/Abstract) OR (strangulated Title/Abstract ) OR (irreducible Title/Abstract))

Additional 305 articles were screened from 4 additional searches:

- (((((groin hernia) OR (inguinal hernia)) OR (femoral hernia)) AND (incarcerated hernia)) OR (strangulated hernia)) AND (definition)
- (((((((groin hernia) OR (inguinal hernia)) OR (femoral hernia)) AND (incarcerated hernia)) OR (strangulated hernia)) OR (irreducible hernia)) AND (emergency surgery) AND (outcomes)
- ((((((groin hernia) OR (inguinal hernia)) OR (femoral hernia)) AND (strangulated hernia)) OR (incarcerated hernia)) AND (laparoscopic repair))
- (((((incarcerated groin hernia) OR (incarcerated inguinal hernia)) OR (incarcerated femoral hernia)) AND (contaminated wound)) OR (dirty wound)) AND (mesh repair)

**28. Non-commercial mesh**

**PICO:**

Patient: Adult patients who have had an open inguinal hernia anterior mesh repair

Intervention: Non-commercial mesh

Control: Commercial mesh

Outcome: SSO, mortality, sepsis, recurrence, cost

Search terms: “Hernia” AND “Mosquito net” OR “non-commercial mesh” OR “low cost mesh”. Limits: From 2015-june 2020.

**Supplementary Figures**

**6a. Tissue repair**

**Prisma: Key Question 1**

Records identified through Pubmed, WoS, Cochrane and SCOPUS

**63 papers**

Additional records identified through cross referencing

**5 papers**

**Identification**

Records after duplicates removed
**68 papers**

**Records excluded**

- No PICO
- Not related

Low quality

**31 papers**

Records screened
**68 papers**

**Screening**

**Full-text excluded**

- Studies about diagnosis
- Past guidelines

**16 papers**

Full-text articles assessed for eligibility

**37 papers**

**Eligibility**

Studies included in qualitative synthesis
**21 papers**

15

**Included**

**Prisma: Key Question 2**

Additional records identified through cross referencing

**0 papers**

Records identified through Pubmed, WoS, Cochrane and SCOPUS

**64 papers**

**Identification**

Records after duplicates removed
**64 papers**

**Records excluded**

- No PICO
- Not related

Low quality

**26 papers**

Records screened
**64 papers**

**Screening**

**Full-text excluded**

- Studies about diagnosis
- Past guidelines

**17 papers**

Full-text articles assessed for eligibility

**38 papers**

**Eligibility**

**Included**

Studies included in qualitative synthesis
**21 papers**

6d. Pre-peritoneal repair

**Identification of studies via other methods**

**Identification of studies via databases and registers**

Records removed *before screening*:

Duplicate records removed
(n = 0)

Records marked as ineligible (2015-2020) after previous HerniaSurge (n = 7657)

Records removed for other reasons (n = 10)

Records identified from:

Citation searching (n = 10)

Handsearching (n = 2)

Records excluded (n = 44)

Reports excluded (n = 29)

No comparable study (n = 15)

Reports sought for retrieval

(n = 12)

Reports not retrieved

(n = 0)

Not relevant to the research question and outcome (n =11)

Records identified:

Databases (n = 7733)

**Identification**

Records screened

(n = 66)

Reports sought for retrieval

(n = 22)

Reports not retrieved

(n = 12)

**Screening**

Reports assessed for eligibility

(n = 22)

Reports assessed for eligibility

(n = 0)

Studies included (n = 11)

2 meta analyses, 7 RCTs and 2 registry analysis

**Included**

6f. Endo-laparoscopic repair

**Full-text excluded**

(n = 15)

- Including bilaterals or recurrences (n=7)
- Different open techniques or no inclusion of Lichtenstein (n=8)

Full-text articles assessed for eligibility

(n = 27)

Studies included in qualitative synthesis

(n = 12)

Records after duplicates removed

(n = 358)

**Records excluded**

(n = 331)

Records screened

(n = 358)

Additional records identified through cross referencing

(n = 4)

Records identified through Pubmed, WoS, Cochrane and SCOPUS

(n = 354)

**Included**

**Eligibility**

**Screening**

**Identification**

8. Occult hernia

**
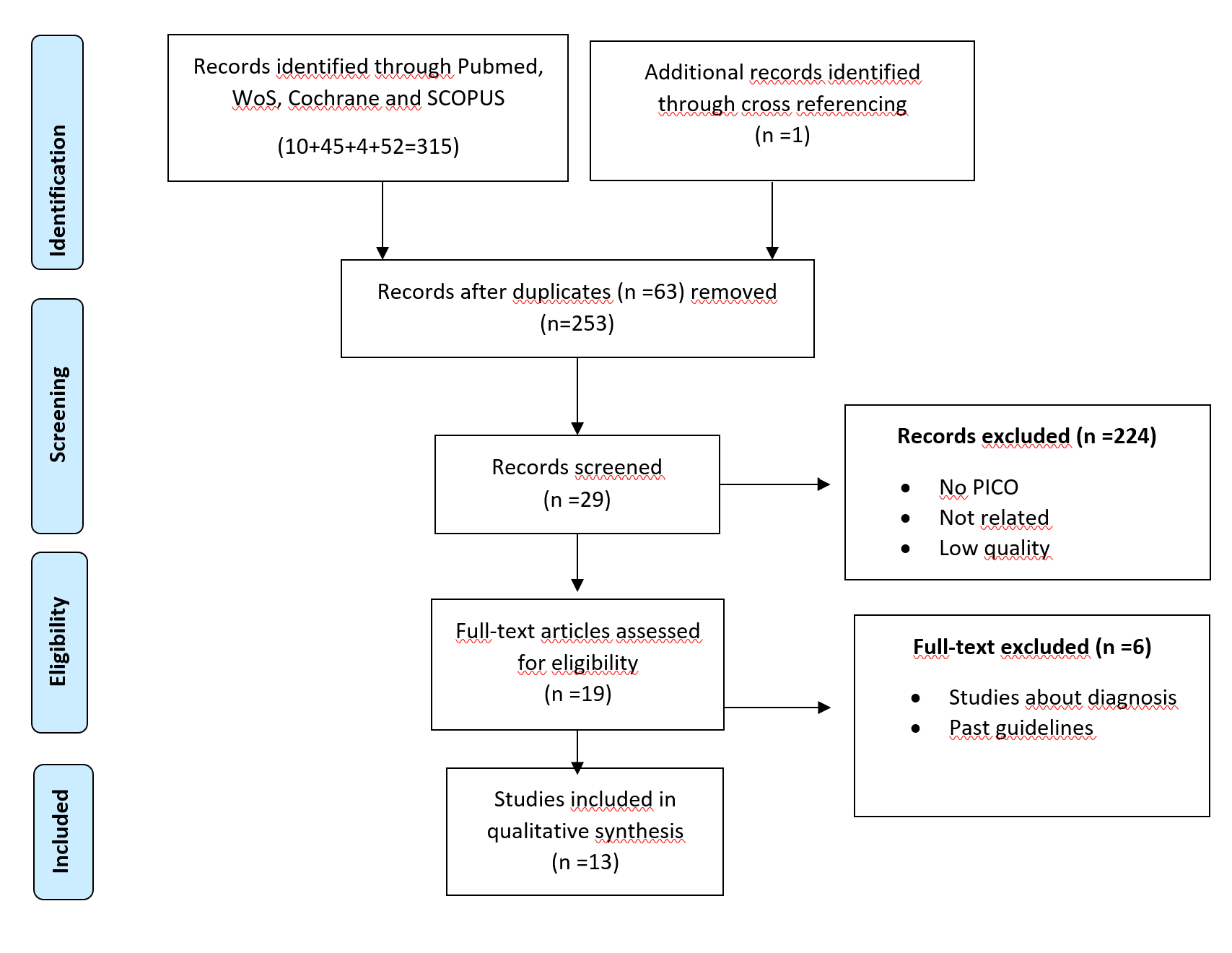
**

10. Mesh

**
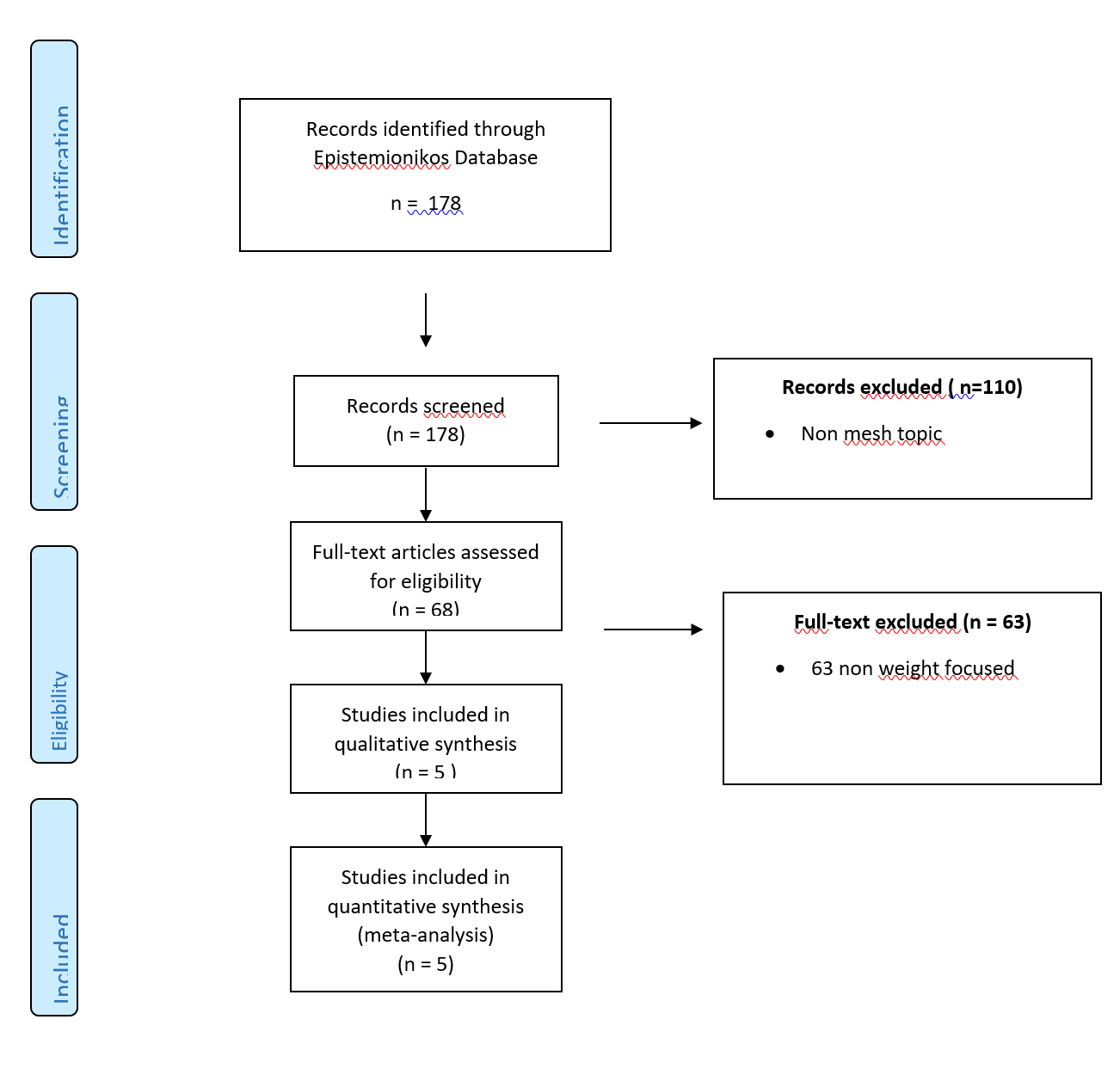
**

12. Antibiotic prophylaxis


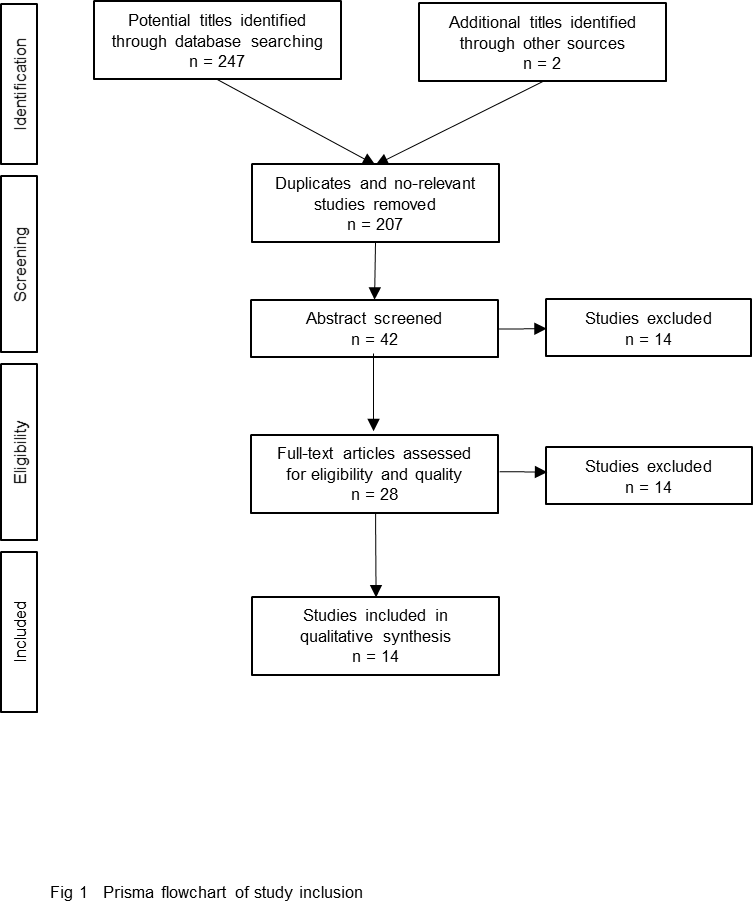


13. Anesthesia


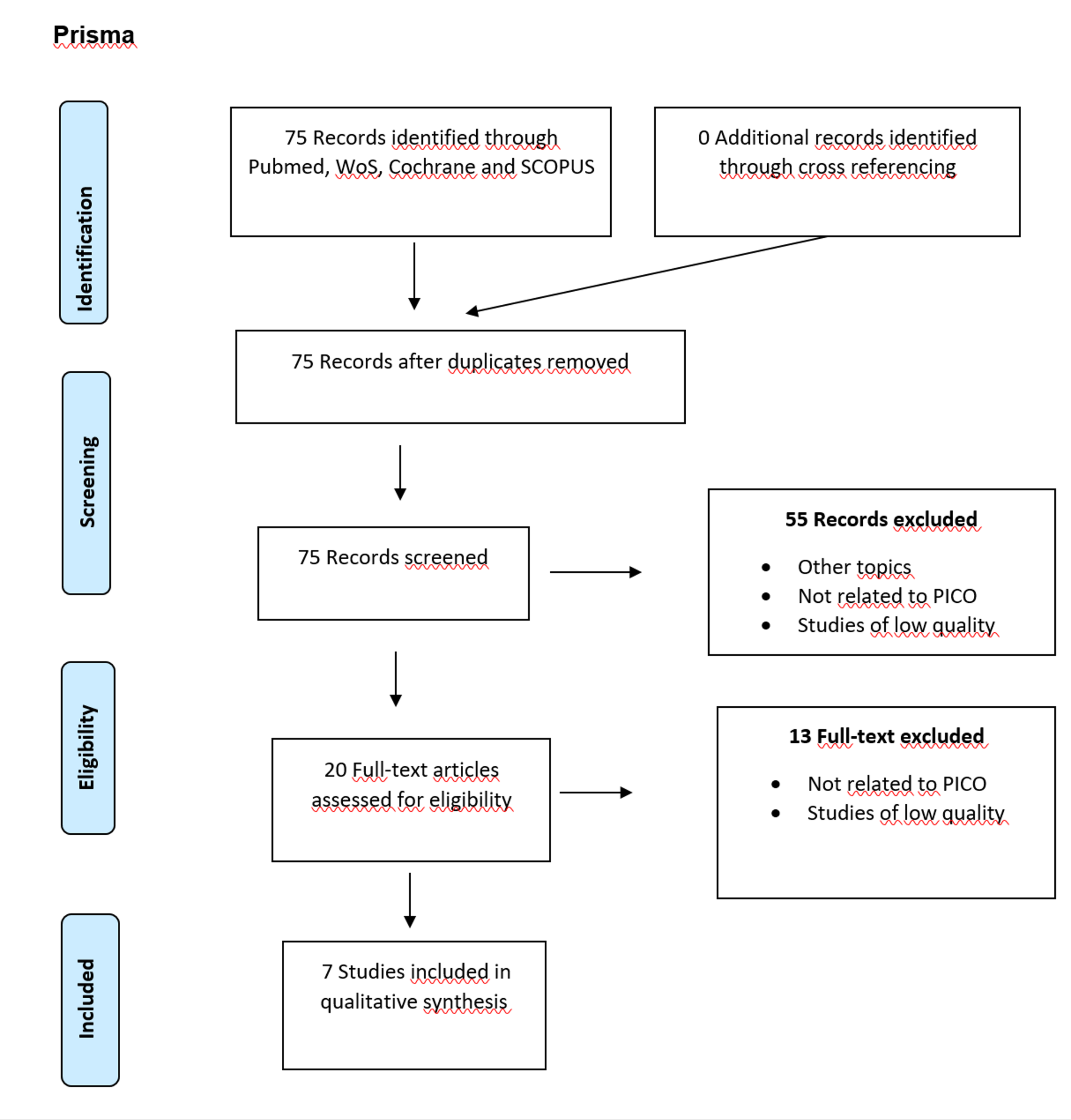


19. CPIP

|  |
| --- |
| **Figure 1. Prisma flowchart of study inclusion** |

21.emergency**
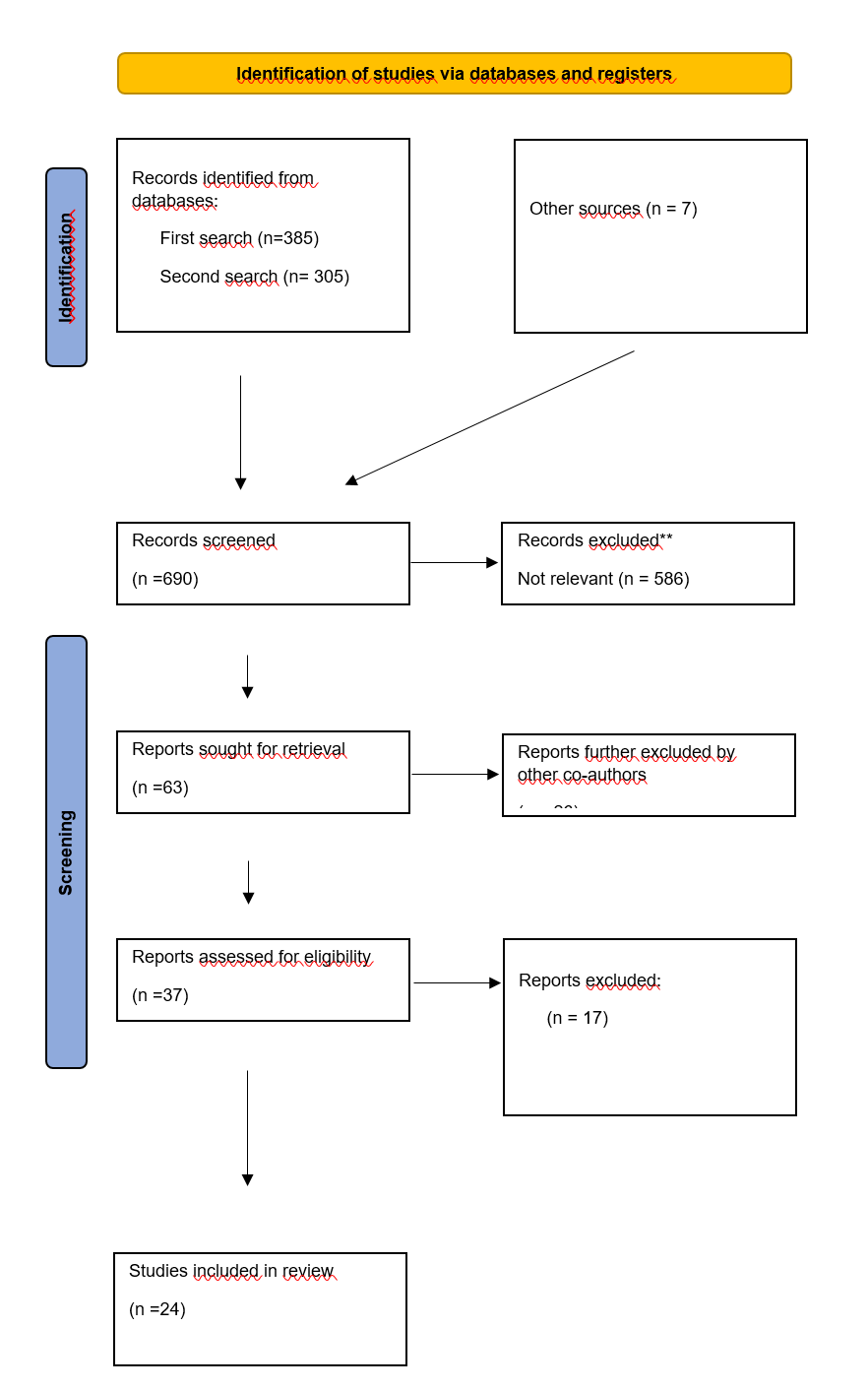
**

28. Non-commercial mesh

**
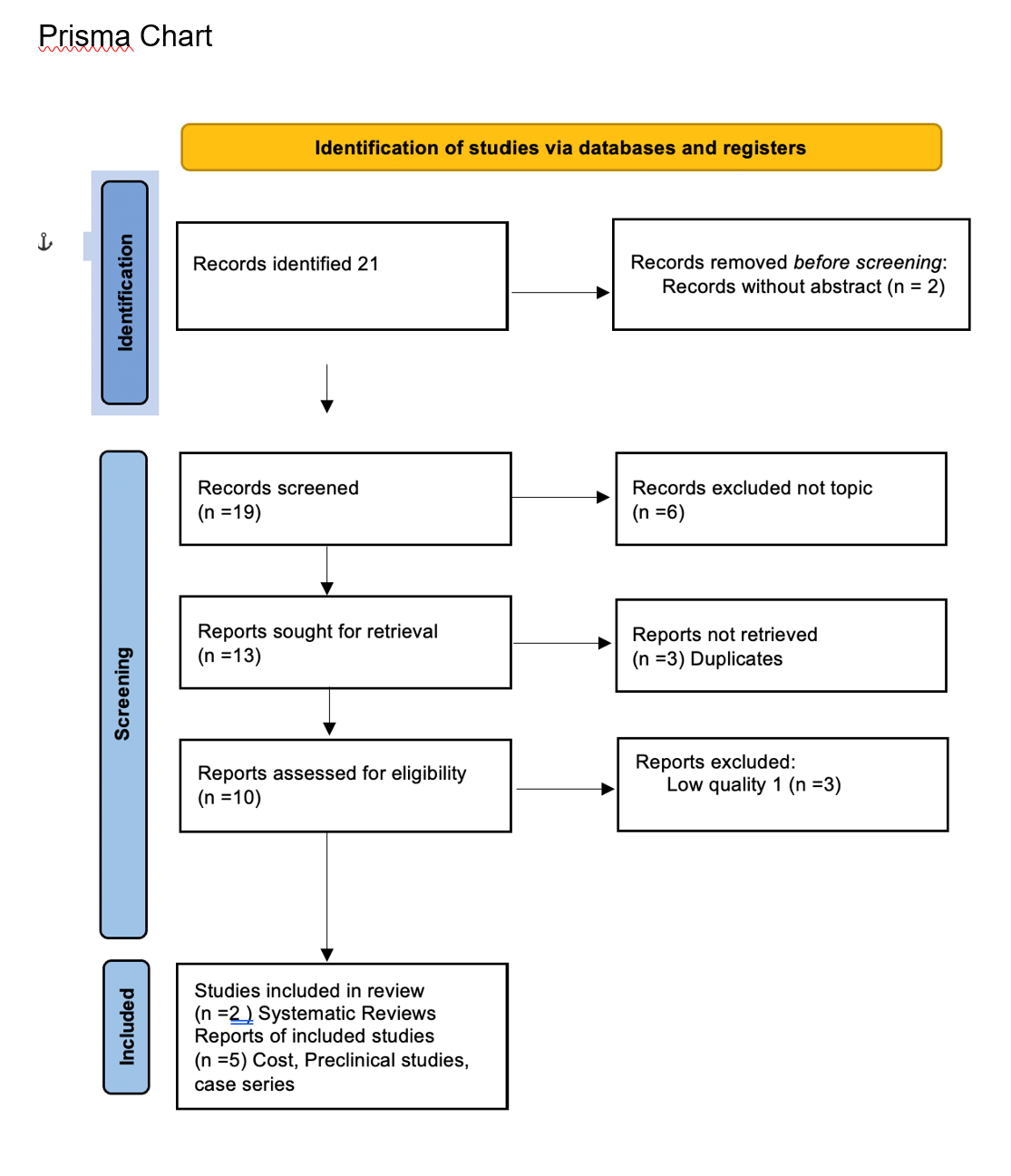
**

**Supplementary Table**

**6a. Tissue repair**

21 Selected Studies for KQ 6ª mesh versus non-mesh repair

| **Author, Year, Journal,**  **(Study Type)** | **Comparison**  **(n of patients)** | **Recurrence** | **Chronic pain** | **Other Complications** | **SIGN** |
| --- | --- | --- | --- | --- | --- |
| Lockhart K et al  2018 Cochrane Database Syst Rev.  **Systematic Review and Meta-Analysis [14]** | **25 RCTs**  6,293 patients | Mesh reduces risk of recurrence.  In absolute numbers, one hernia recurrence was prevented for every 46 mesh repairs compared with non-mesh repairs | - | - | **High Quality (++)** |
| Köckerling F, Koch A et al  2018 World J Surg  **Database Study [30]** | Shouldice versus Lichtenstein (n = 2115/2608; 81.1%), Shouldice versus TEP (n = 2225/2608; 85.3%) and Shouldice versus TAPP (2400/2608; 92.0%) | Shouldice, Lichtenstein TAPP and TEP equal after one year in selected cases | Shouldice, Lichtenstein TAPP and TEP equal after one year in selected cases | Shouldice, Lichtenstein TAPP and TEP equal after one year in selected cases | **High Quality (++)** |
| Mohamedahmed AYY et al  2020 World J Surg.  **Sytematic Review and Meta-Analysis [15]** | **8 RCT**  1,551 Desarda  1,626 Lichtenstein | Desarda and Lichtenstein equal |  | There was a lower rate of overall post-operative complications [P = 0.003], seroma [P = 0.0004] and surgical site infections (SSIs) [P = 0.04] in the Desarda group. | **High Quality (++)** |
| Ge H et al  2018 Int J Surg  **Review and Meta-Analysis [16]** | **8 RCTs**  500 Desarda  516 Lichtenstein | Desarda and Lichtenstein equal | Desarda and Lichtenstein equal | Desarda and Lichtenstein equal | Acceptable (+) |
| Emile SH et al  2018 Hernia  **Review and Meta-Analysis [17]** | **6 RCTs**  1,099 Desarda  1,155 Lichtenstein | Desarda and Lichtenstein equal | Desarda and Lichtenstein equal | The overall complication rate of LT was significantly higher than DT (OR = 1.86; P < 0.001). LT had significantly higher rates of seroma formation and surgical site infection | Acceptable (+) |
| Gedam BS et al  2017, Int. J Surg  **RCT [26]** | 92 Desarda  95 Lichtenstein | Desarda and Lichtenstein equal | Desarda and Lichtenstein equal | Less Postoperative pain in Desarda group | Acceptable (+) |
| Vupputuri H et al  2018, Int J Abdom Wall Hernia Surg  **RCT [27]** | 62 Desarda  61 Lichtenstein | Desarda and Lichtenstein equal after 3 years | Significantly higher number of patients from M group suffered from chronic groin pain compared to the NM group | - | Acceptable (+) |
| Köckerling F, Lorenz R et al  2019 Ann Surg  **Database Study Females [35]** | 15,601 female Patients  1,306 Shouldice | Shouldice, Lichtenstein TAPP and TEP equal after one year | Lichtenstein more chronic pain | Shouldice, Lichtenstein TAPP and TEP equal | Acceptable (+) |
| Finch DA et al  2019 Hernia  **Systematic Review and Meta-Analysis [18]** | **6 RCTs and**  **4 observational studies**  1,480 + 1,564 patients  Darn and Lichtenstein | Darn and Lichtenstein equal | Darn and Lichtenstein equal | Darn and Lichtenstein equal | Acceptable (+) |
| Öberg S et al.  2018 Surgery  **Systematic Review and Meta-Analysis [19]** | **23 RCTs**  5,444 patients | - | prevalences of pain after nonmesh repairs and mesh repairs were similar after median 1.4 years | - | Acceptable (+) |
| Clyde DR et al  2020 Hernia  **RCT [21]** | 297 femoral hernias | Of the 138 cases with complete follow-up, 25 patients experienced recurrence at 5 years (18%), with 60% of recurrences evident within the first post-operative year. | - | - | Acceptable (+) |
| Dong Z et al  2018 Reproductive Health  **Systematic Review and Meta-Analysis [20]** | **29 RCTs**  36,552 patients | - | - | - | Acceptable (+) |
| Claus CMP et al  2019 Rev Col Bras Cir  **Review**  Brasilian Guidelines [13] | NA n patients | Meshes should be used to reduce hernia recurrence. | The incidence of postoperative chronic pain with mesh use is similar to or lower than without its use. | In case of strangulated hernias with contamination of the operative field (intestinal loop perforation, peritonitis or abscess), the use of meshes should be avoided. | Acceptable (+) |
| Youssef T et al  2015 Int J Surg  **RCT [22]** | 85 Desarda  86 Lichtenstein | Desarda and Lichtenstein equal after 2 years | Desarda and Lichtenstein equal after 2 years | - | Acceptable (+) |
| Ahmed AE et al  2018 Int Surg J  **RCT [53]** | 65 Desarda  65 Lichtenstein | Desarda and Lichtenstein equal after 6 months | Desarda and Lichtenstein equal after 6 months | Desarda and Lichtenstein equal after 6 months | Acceptable (+) |
| Arafa AA.  2020 The Egyp. J. of Surgery  **RCT** | 40 Desarda (DT)  40 Lichtenstein (LT)  (80 patients in 3 years!?) | Desarda and Lichtenstein equal (2 patient in DT, 1 patient inLT group) | Chronic groin pain and numbness, nerve entrapment are equal (DT: 2 patients, LT: 4 patients) | - | Acceptable (+) |
| Rodriguez PRL et al  2018 Biomed J Sci & Tech Res  **RCT [54]** | (**2225** patients with **2336 hernias** from 2002-2016)  1,075 Desarda  1,150 Lichtenstein, (median Follow-up: 6.5 years) | Desarda (recurrence 0.6%) and Lichtenstein (recurrence 0.4%) equal | Desarda and Lichtenstein equal | 11 cases of mesh-infection in LT group, morbidity was significant more in LT group (6.0%) vs. DT group (3.6%);  0.4% of patients in the Lichtenstein group required a further surgical intervention for either recurrence or sepsis which was significantly higher than the Desarda group, | Acceptable (+) |
| Olasehinde O et al., Hernia. 2016;  **RCT [24]** | 34 Darning repair  33 Lichtenstein  (mean follow-up 7.5 months) | no recurrence in both groups after 6 months | A greater proportion of patients who had darning  (47.1 %) experienced pain at 10 days postoperatively  compared to those who had Lichtenstein repair (24.2 %). While none of the patients in the Lichtenstein repair group  had pain at 1 month postoperatively, pain still persisted in 8.8 % of patients in the darning group.  But: **chronic pain** (one patient (2.9%) in each group after 3 month) is **equal** in both groups | - | Acceptable (+) |
| Barbaro A et al.  2017 Hernia  **RCT [25]** | 72 SHOULDICE  35 TEP  20-year Follow-up | There were 7/72 (9.7%) recurrences in the open group and 9/35 (25.7%) recurrences in the laparoscopic group. This difference in recurrence rates was statistically significant (HR = 2.94; 95% CI 1.05-8.25; p = 0.041.) | - | - | Acceptable (+) |
| Haastrup et al  2017  **Database Study [31]** | 52,281 primary repairs of open indirect inguinal hernia were included of which 49,951 were Lichtenstein repairs and 2330 were annulorrhaphies. | The overall reoperation rates increased year after year in both groups but the 18-29 year old males had a significant lower risk of reoperation after annulorrhaphy compared with all other age groups (cumulative reoperation rate 8.1% versus 12.5%, log rank p = 0.001). | - | - | Acceptable (+) |
| Djokovic et al  2021  **RCT [28]** | 300 patients  100 Lichtenstein  100 Shouldice  100 TIPP | - | The visual analog scale (VAS) score after 6, 12, 24 and 48 h, and 14 d was lower in TIPP than the Lichtenstein and Shouldice groups (*p* < .0001). The satisfaction level was higher in TIPP than in the Lichtenstein and Shouldice groups (*p* < .0001). | - | Acceptable (+) |

21 Selected Studies for KQ 6b – best non-mesh repair

| **Author, Year, Journal,**  **(Study Type)** | **Comparison**  **(n of patients)** | **Recurrence** | **Chronic pain** | **SIGN** |
| --- | --- | --- | --- | --- |
| Köckerling F, Koch A et al  2018 World J Surg  **Database Study** | Shouldice versus Lichtenstein (n = 2115/2608; 81.1%), Shouldice versus TEP (n = 2225/2608; 85.3%) and Shouldice versus TAPP (2400/2608; 92.0%) | Shouldice, Lichtenstein TAPP and TEP equal after one year in selected cases | Shouldice, Lichtenstein TAPP and TEP equal after one year in selected cases | **High Quality**(++) |
| Bracale U. et al,  2019 Int J Surg,  **Systematic Review and Network Meta-Analysis** | **14 RCTs,**  2791 patients,  Comparing Lichtenstein respectively with Desarda and Shouldice techniques | Desarda and Shouldice equal | early postoperative pain, numbness and chronic pain are similar | **High quality** (++) |
| Mohamedahmed AYY et al  2020 World J Surg.  **Sytematic Review and Meta-Analysis** | **8 RCT**  1,551 Desarda  1,626 Lichtenstein | Desarda and Lichtenstein equal | - | **High Quality** (++) |
| Ge H et al  2018 Int J Surg  **Review and Meta-Analysis** | **8 RCTs**  500 Desarda  516 Lichtenstein | Desarda and Lichtenstein equal | Desarda and Lichtenstein equal | Acceptable (+) |
| Emile SH et al  2018 Hernia  **Review and Meta-Analysis** | **6 RCTs**  1,099 Desarda  1,155 Lichtenstein | Desarda and Lichtenstein equal | Desarda and Lichtenstein equal | Acceptable (+) |
| Gedam BS et al  2017, Int. J Surg  **RCT** | 92 Desarda  95 Lichtenstein | Desarda and Lichtenstein equal | Desarda and Lichtenstein equal | Acceptable (+) |
| Vupputuri H et al  2018, Int J Abdom Wall Hernia Surg  **RCT** | 62 Desarda  61 Lichtenstein | Desarda and Lichtenstein equal after 3 years | Significantly higher number of patients from M group suffered from chronic groin pain compared to the NM group | Acceptable (+) |
| Köckerling F, Lorenz R et al  2019 Ann Surg  **Database Study Females** | 15,601 female Patients  1,306 Shouldice | Shouldice, Lichtenstein TAPP and TEP equal after one year | Lichtenstein more chronic pain | Acceptable (+) |
| Finch DA et al  2019 Hernia  **Systematic Review and Meta-Analysis** | **6 RCTs and**  **4 observational studies**  1,480 + 1,564 patients  Darn and Lichtenstein | Darn and Lichtenstein equal | Darn and Lichtenstein equal | Acceptable (+) |
| Clyde DR et al  2020 Hernia  **RCT** | 297 femoral hernias | Of the 138 cases with complete follow-up, 25 patients experienced recurrence at 5 years (18%), with 60% of recurrences evident within the first post-operative year. | - | Acceptable (+) |
| Youssef T et al  2015 Int J Surg  **RCT** | 85 Desarda  86 Lichtenstein | Desarda and Lichtenstein equal after 2 years | Desarda and Lichtenstein equal after 2 years | Acceptable (+) |
| Ahmed AE et al  2018 Int Surg J  **RCT** | 65 Desarda  65 Lichtenstein | Desarda and Lichtenstein equal after 6 months | Desarda and Lichtenstein equal after 6 months | Acceptable (+) |
| Arafa AA.  2020 The Egyp. J. of Surgery  **RCT** | 40 Desarda (DT)  40 Lichtenstein (LT)  (80 patients in 3 years!?) | Desarda and Lichtenstein equal (2 patient in DT, 1 patient inLT group) | Chronic groin pain and numbness, nerve entrapment are equal (DT: 2 patients, LT: 4 patients) | Acceptable (+) |
| Rodriguez PRL et al  2018 Biomed J Sci & Tech Res  **RCT** | (**2225** patients with **2336 hernias** from 2002-2016)  1,075 Desarda  1,150 Lichtenstein, (median Follow-up: 6.5 years) | Desarda (recurrence 0.6%) and Lichtenstein (recurrence 0.4%) equal | Desarda and Lichtenstein equal | Acceptable (+) |
| [Olasehinde](https://pubmed.ncbi.nlm.nih.gov/?term=Olasehinde+O&cauthor_id=27146504) O et al., Hernia. 2016;  **RCT** | 34 Darning repair  33 Lichtenstein  (mean follow-up 7.5 months) | no recurrence in both groups after 6 months | A greater proportion of patients who had darning  (47.1 %) experienced pain at 10 days postoperatively  compared to those who had Lichtenstein repair (24.2 %). While none of the patients in the Lichtenstein repair group  had pain at 1 month postoperatively, pain still persisted in 8.8 % of patients in the darning group.  But: **chronic pain** (one patient (2.9%) in each group after 3 month) is **equal** in both groups | Acceptable (+) |
| Barbaro A et al.  2017 Hernia  **RCT** | 72 SHOULDICE  35 TEP  20-year Follow-up | There were 7/72 (9.7%) recurrences in the open group and 9/35 (25.7%) recurrences in the laparoscopic group. This difference in recurrence rates was statistically significant (HR = 2.94; 95% CI 1.05-8.25; p = 0.041.) | - | Acceptable (+) |
| Gasior et al  2015  **Retrospective Cohort Study** | 210 patients with high ligation  phone interviews at 18.6 to 159.5 months postrepair.  Mean age was 14.6 ± 1.8 (range: 12.0-19.0 years). | There were four (1.9%) patients with a second operation, two of which confirmed a recurrent hernia. | Fourteen patients had pain (6.7%) and five had numbness (2.4%). | Acceptable (+) |
| Van Kerckhofen et al.  2016  **Retrospective Cohort Study** | 234 patients with Herniotomy | recurrence rate was 4.7 %. When we focus on the patients in the ages ranging from 18 to 25 years old at the time of the surgery, we found a recurrence rate of 0 versus 9.2 % in ages 26–40. | - | Acceptable (+) |
| Haastrup et al  2017  **Database Study** | 52,281 primary repairs of open indirect inguinal hernia were included of which 49,951 were Lichtenstein repairs and 2330 were annulorrhaphies. | The overall reoperation rates increased year after year in both groups but the 18-29 year old males had a significant lower risk of reoperation after annulorrhaphy compared with all other age groups (cumulative reoperation rate 8.1% versus 12.5%, log rank p = 0.001). | - | Acceptable (+) |
| Taylor et al  2020 J. Pediatr. Surg  **RCT** | 296 patients | 6.0% recurrence rate, and all patients that developed recurrence underwent a reoperation. The median time from surgery to reoperation for recurrence was 3.1 years. | - | Acceptable (+) |
| Malik et al  2021  **Retrospective Study** | 198 patients  15 years follow-up | After 15 years of follow-up,  three recurrences were found (1.5%). Recurrences occurred 2, 3, and 5 years after the surgery. | Twenty-eight patients (14.4%) reported a rare occurrence of mild  pain while performing certain activities. Three patients reported persistent chronic pain (1.5%). | Acceptable (+) |

6d. Pre-peritoneal repair

**Table Quality scores**

11 selected studies for KQ1

| **Author, Year, Journal,**  **(Study Type)** | **Comparison**  **(n of patients)** | **Recurrence** | **Chronic pain** | **Other Complications** | **Others** | **Quality of studies** |
| --- | --- | --- | --- | --- | --- | --- |
| Arslan K, 2015  Hernia  (RCT)^4^ | 101 TIPP modified Kugel  vs.  105 Lichtenstein | 3% Kugel  1% Lichtenstein  - | 0.66% Kugel  0.87% Lichtenstein after 2 years | More postoperative Pseudohernias, but less operation time in the Kugel group | Kugel equal to Lichtenstein  benefits regarding chronic pain | Low (-) |
| Bökkerink WJV 2019 BJS  (RCT)  TULIP Trial^5^ | 119 TIPP  vs.  132 Lichtenstein | 1.7% TIPP  3.8% Lichtenstein | 2.5% TIPP  3.0% Lichtenstein  after 1 year | - | TIPP equal to Lichtenstein | Aceptable (+) |
| Cadanova D, 2017 Hernia  (RCT)^6^ | 122 TIPP  vs.  116 Lichtenstein with Progrip | 2.5% TIPP  2.6% Lichtenstein | 6.7% TIPP  4.2% Lichtenstein  After 1 year | More early postoperative pain 2 weeks 3 months , more Seroma, more hypersensitivity more scrotal pain in Lichtenstein group | TIPP equal to Lichtenstein, benefits early postoperative time | Aceptable (+) |
| Djokovic A  2019 Acta Chirurgica Belgica  (RCT)^7^ | 100 TIPP  vs.  100 Shouldice  vs.  100 Lichtenstein | No data | Less in the TIPP group  2 weeks | Less acute pain  Higher patients satisfaction in TIPP group | TIPP better outcome than Shouldice and Lichtenstein but more expensive | Aceptable (+) |
| Oprea V  2019 Chirurgia  (RCT) ^8^ | 104 TIPP  vs.  101 Lichtenstein  Only complex cases  Recurrences, large hernias, femoral | 2% TIPP  3% Lichtenstein | 3% TIPP  9% Lichtenstein  After 1 year | Less severe pain in TIPP group | TIPP significantly less recurrences and pain after 1 year and less severe pain | Aceptable (+) |
| Romain B  2018 Hernia  (Database Study)^11^ | 1259 TIPP  vs.  1012 Lichtenstein  vs.  1414 TEP  vs.  1905 TAPP | No data | 5.9% TIPP  7.8% Lichtenstein  6.2% TEP  7.5% TAPP after 2 years | No difference, only independent risk factor for CPIP is severe pain at day one after surgery | No difference | Aceptable (+) |
| Sharma P  2015 Health Technology Assess  (Meta-Analysis with 12 RCT´s)^2^ | 771 TIPP  vs.  797 Lichtenstein | Non significant favor for TIPP | Non significant favor for TIPP | TIPP patients showed lower incidence of pain and numbness, less recurrences,  less complications | TIPP patients Significant quicker return to normal activities | Aceptable (+) |
| Suwa K  2020 Hernia  (RCT)^9^ | 100 TIPP modified Kugel  vs.  100 Lichtenstein | 1% TIPP  0% Lichtenstein | 7.2% TIPP  11.1% Lichtenstein | TIPP patients less complications, less chronic pain, NRS less foreign body sensation after one year  NRS less numbness after one month | TIPP patients significant lower operation time | High (++) |
| Decker E  2019 Hernia  (Meta-Analysis with 7 RCT)^3^ | 691 Gilbert  with PHS  vs.  686 Lichtenstein | 1.3% recurrences pooled  Non significant favor for Gilbert | Non significant favor for Gilbert | Non significant favor for Gilbert  in case of operating time,  complications reinterventions | Equal results for Gilbert and Lichtenstein, both should be possible | Aceptable (+) |
| Magnusson J  2016 Hernia  (RCT)^10^ | 99 Gilbert PHS  vs.  102 Gilbert UHS  vs.  108 Lichtenstein | 1.6% recurrences  Not enough power for any recommendation  No differences | No difference after 3 years | - | Equal results regarding pain, discomfort and QoL after 3 years | Low (-) |
| Magnusson J  2018 Hernia  (Database Study)^12^ | 1229 Gilbert PHS  vs.  78230 Lichtenstein | 1.5% Gilbert  2.7% Lichtenstein | No data | Re-operating time after Gilbert was non-significant shorter, not more complications | Significant lower recurrence rate after Gilbert  Re-Operations are not more complicated after Gilbert | Aceptable (+) |

4 selected studies for KQ2

| **Author, Year, Journal,**  **(Study Type)** | **Comparison**  **(n of patients)** | **Outcome** | **Recurrence** | **Chronic pain** | **Complications** | **Others** | **Quality of studies** |
| --- | --- | --- | --- | --- | --- | --- | --- |
| Akgüll N, 2016  *Hernia*  (RCT) | TEP vs Stoppa repair  25vs25  (unilateral hernias) | Activity parameters lower extremity muscles | - | - | - | TEP better than Stoppa | Low (-) |
| Kushwaha JK, 2017  *SLEPT*  (RCT) | TEP vs Stoppa repair  20vs20  (Unilateral and bilateral hernias) | QOL  Postop Comp | - | - | No differences | TEP better QOL than Stoppa | Aceptable (+) |
| Haroon M, 2019  *Chirurg* (Observational) | hTIPP vs TEP  46vs44 | Postop Comp | - | - | No differences | - | Aceptable (+) |
| Aksoy N, 2019  Turk J Surg  (RCT) | TEP vs MIP  112vs113  (Kugel) | Compl.  Recurrence  Chronic pain | No differences | No differences | No differences | MIP technique is as safe as TEP repair | Aceptable (+) |

6f. Endo-laparoscopic repair

**Table Quality scores**

| **Author, Year, Journal,**  **(Study type)** | **Comparison**  **(n of patients)** | **Recurrence** | **Pain** | **Learning curve** | **Postoperative recovery** | **Costs** | **Quality of studies** |
| --- | --- | --- | --- | --- | --- | --- | --- |
| **Gutlic, N; 2019**  **BJS**  **(RCT)[2]** | 202 TEP  vs  214 Lichtenstein | 4 TEP (2.2%)  2 Lichtenstein (1%) | 6.9% TEP  9.8% Lichtenstein | No data | Sick leave and recovery time shorter in TEP (p<0.001) | No data | Acceptable (+) |
| **Gürbulak, E.K., 2015**  **Surgery**  **(RCT) [3]** | 64 TEP  vs  70 Lichtenstein | 1 TEP  1 Lichtenstein | No data | No data | No data | Similar duration of hospital stay | High (++) |
| **Sevinç, B; 2019**  **Turk J Med SCI**  **(RCT) [4]** | 147 TEP  vs  155 Lichtenstein | TEP 3.4%  Lichtenstein 5.2%  p=0.45 | TEP 3.4%  Lichtenstein 25.2%  p=0.001 | No data | No data | Lichtenstein longer hospital stay  p=0.001 | High (++) |
| **Pedroso, L; 2017**  **Arq Bras Cir Dig**  **(RCT) [5]** | 30 TAPP  vs  30 Lichtenstein | TAPP 0  Lichtenstein 0 | TAPP 3.6%  Lichtenstein 32.1%  p<0.003 | No data | No data | No data | Aceptable (+) |
| **Bullen, NL; 2019**  **Hernia**  **(SR)[6]** | TEP 1653  TAPP 387  Vs  Lichtenstein 1926 | Laparoscopic vs  Lichtenstein  (OR 1.14, CI 0.51-2.55, p=0.76) | Less pain in laparoscopic vs  Lichtenstein  (OR 0.41, 95% CI 0.30-0.56, p≤0.00001) | No data | No data | No data | Aceptable (+) |
| **Lyu, Y; 2020**  **Medicine**  **(MA)[9]** | TEP 2067  TAPP 1228  Vs  Lichtenstein 2299 | TAPP, TEP, Lichtenstein  (OR (95%CrI): 1.7 (0.56-5.5), 0.85 (0.26-2.0), 0.51 (0.13-0.14)) | Greatest chronic pain for Lichtenstein in rank plot. No difference in pooled network meta-analysis | No data | Return-to-work days longer in Lichtenstein | Lichtenstein has the longest hospital stays but no significant differences | Aceptable (+) |
| **Gavriilidis, P; 2019**  **Hernia**  **(SR)[8]** | TEP 3242  Vs  Lichtenstein 3331 | TEP 6%  Lichtenstein 4%  OR= 1.58 (1.22, 2.04) p=0.005 | TEP 11%  Lichtenstein 13%  OR= 0.81 (0.66, 1.00) p=0.05 | No data | Return to usual activities shorter for TEP  (p<0.001) | No differences in time to discharge | Acceptable (+) |
| **Aiolfi, A; 2019**  **Hernia**  **(SR) [7]** | TEP 15687  TAPP 17112  rTAPP 103  Lichtenstein 18135 | Similar for:  TAPP vs Lichtenstein  TEP vs Lichtenstein | Similar for:  TAPP vs Lichtenstein  TEP vs Lichtenstein | No data | No data | Similar length of hospital stay | Aceptable (+) |
| **Aiolfi, A; 2021**  **Ann Surg**  **(Network MA)[10]** | TEP 3012  TAPP 1269  Lichtenstein 3496 | Similar for:  TAPP vs Lichtenstein  TEP vs Lichtenstein | Less pain in laparoscopic vs  Lichtenstein | No data | Return to work significantly shorter for TEP and TAPP | Comparable length of hospital stay | High (++) |
| **Köckerling, F; 2019**  **Ann Surg**  **(Cohort)[11]** | TEP 14559  TAPP 21236  Lichtenstein 22111 | Similar for:  TAPP vs Lichtenstein  TEP vs Lichtenstein | Less pain in laparo-endoscopic vs  Lichtenstein | No data | No data | No data | High (++) |
| **Köckerling, F; 2016**  **Surg Endosc**  **(Cohort) [12]** | TEP 6833  Lichtenstein 10555 | Comparable  p=0.146 | TEP less pain at rest and on exertion. No differences on chronic pain | No data | No data | No data | High  (++) |
| **Quispe, MRF; 2019**  **Acta Cir Bras**  **(Cohort)[13]** | TAPP 22  Lichtenstein 37 | TAPP 1 (2.7%)  Lichtenstein 0 | Comparable | No data | No data | No data | Low (-) |

8. Occult hernia

| **Author And Year** | **Study Type** | **Population** | **Comparison** | **Outcome** | **Results** | **Sign Checklist** |
| --- | --- | --- | --- | --- | --- | --- |
| Dhanani, 2020 | Syst Rev | Laparoscopic IHR (TAPP/TEP)  5000 patients | Contralateral Exploration Vs No Exploration | Incidence Of Occult Inguinal Hernia and outcomes | Occult Hernia 14.6% (range 7.3–50.1)  1.5% Postoperative Complications for concomitant repair.  4.5% Subsequent Repair. | Acceptable (+) |
| Park JB,  2022 | Syst Rev | Laparoscopic IHR (TAPP/TEP)  1774 patients | unilateral inguinal repair vs prophylactic bilateral inguinal repair | Peroperative Outcomes and recovery | similar length of stay, return to activities, complications. Mean operating time lower for unilateral (mean  − 14.57 min, 95%CI − 25.59, − 3.45).  pain scores lower for unilateral repair (− 0.33 units, 95%CI − 0.48, − 0.18). | Acceptable (+) |
| Dickens, 2018 | Retrospective | Robotic unilateral IHR  652 patients | Unilateral Repair Vs Simultaneous Contralateral Repair | Incidence Of Complications During Intraoperative Contralateral Exploration | Similar surgical and postoperative morbidity  Longer Operative Time for Contralateral Repair (+19min, P<0.0001) | Acceptable (+) |
| Kou, 2021 | Retrospective | 432 Patients With PD Catheter Undergoing Laparoscopic Examination For Occult IH | RLEOH Vs Non-RLEOH | Risks/Benefits Of Contralateral Examination For Occult Hernia | RLEOH Group  Metachronous Ih Repair: (HR=0.426; 95% Ci 0.195–0.930, P=0.032)  A (Occult Hernia Repair)  B (No Occult Hernia)  C (Occult Hernia Without Repair)  Symptomatic IH After PD In A,B,C,Non-RLEOH  (0 Vs 5.6 Vs 22.2 Vs 13.4%) | Acceptable (+) |
| Ota, 2021 | Retrospective | 259 Patients Undergoing Laparoscopic IHR (TEP) | Occult Vs Non Occult | Incidence Of Bilateral And Contralateral Occult Hernia | Operative Time: (166±61 Min Vs 140±50 Min, P= <0.5)  Recurrence: (1.5% Vs 0.4% P=0.13) | Acceptable (+) |
| Kebabci, 2021 | Retrospective | 109 Patients Undergoing Laparoscopic IHR (TEP) | Radiological Diagnosis (Us) Vs Clinical Diagnosis | % Results And Complications In Patients With Contralateral Hernia | Unilateral Repairs Vs Bilateral Repairs  Morbidity Rate: (7.1% Vs 3.8%; P= 0.6)  Recurrence Rate: (3.6% Vs %.7% P= 0.6) | Acceptable (+) |
| Aly, 2021 | Retrospective | 76 Patients With Inguinale Hernia | Conservative Vs Surgical Management | Patient-Reported Outcomes (EuraHS-Qol) | No Differences  Pain at the site of hernia  (4.3 vs 5.4, p=0.535)  Restrictions of activities  (5.0 vs 7.4, p=0.406)  Cosmetic discomfort  (0.7 vs 1.2 p=0.289) | Acceptable (+) |

**Table Quality scores**

10. Mesh

**Table Quality scores**

| **AUTHOR AND YEAR** | **STUDY TYPE** | **POPULATION** | **COMPARISON** | **OUTCOME** | **RESULTS** | **SIGN CHECKLIST** |
| --- | --- | --- | --- | --- | --- | --- |
| Bona, 2017 | RCT | 808 patients Open IHR (Lichtenstein) | Lightweight vs heavyweight mesh | CPIP, discomfort and QoL | pain LW 25% vs HW 26% (ns)  QoL LW 84% vs HW76% no problem (p 0.01)  Discomfort LW 2.72 (± 2.2) vs HW 2.84(±2.1)  (p 0.08) | Low quality  (-) |
| Carro, 2018 | RCT | 67 patients Open bilateral IHR (Lichtenstein) | Lightweight vs heavyweight mesh | Pain, complications  recurrence  Sensation foreign body | Pain LW 0.75 ± 1.51 vs. HW 0.78 ± 1.37 (ns)  Recurrence LW 1.7% vs. HW 0 (ns)  Complications LW 6.8% vs. HW 8.6%  FbS  LW 5.2% vs. HW 10.2% (ns) | Low quality  (-) |
| Demetrashvili, 2014 | RCT | 226 patients Open IHR (Lichtenstein) | Lightweight vs heavyweight mesh | Pain, complications  recurrence  Sensation foreign body | Pain LW 6.3% vs. HW 8.8% ns  Recurrence LW 1.04% vs HW 0.98% ns  Complications LW 8,3% vs 9.8% ns  Fbs LW 6.3% vs HW 16.7% p 0.03 | Acceptable (+) |
| Lee, 2017 | RCT | 47 patients Open IHR (Lichtenstein) | Lightweight vs heavyweight mesh | Pain, Qol  recurrence  Sensation foreign body | Pain LW 0.7 ± 1.1 vs HW 0.8 ± 1.4 p 0.01  QoL LW 3.8 ± 6.6 vs HW6 ±13  p 0.001  recurrence 0  Fbs LW 4.2% vs HW 30.4% p0.02 | Acceptable (+) |
| Rutegard, 2018 | RCT | 412 patients Open IHR (Lichtenstein) | Lightweight vs heavyweight mesh | Pain, Qol  recurrence  Sensation foreign body | Pain no difference  Fbs LW 14% vs HW 23% p 0.05  Discomfort LW 18% vs HW 28.7% p 0.03  Qol LW better 91.8% vs HW better 90.8% ns  Recurrence 2,4% both groups | Acceptable (+) |
| Bakker, 2020 (Surgery) | SR+MA | Open IHR (Lichtenstein)- 21 studies- 4576 patients | Lightweight vs heavyweight mesh | CPIP  recurrence | CPIP LW 234/ 1603 vs. HW 322 /1683 RR 0.78; 95% CI: 0.64-0.96  Recurrence LWM 42/ 2068 vs. HWM 34/ 2132 RR 1.22; 95% CI, 0.76-1.96 | High quality (++) |
| Melkemichel, 2020 | National Registry study | 23259 patients Open IHR (Lichtenstein) | Lightweight vs lightweight absorbable vs heavyweight mesh | Pain | Pain LW 15.8% vs. LW-ab 15.5% vs. Hw 16.2% | High quality (++) |
| Melkemichel, 2019 | National Registry study | 76495 patients Open IHR (Lichtenstein) | Lightweight vs lightweight absorbable (PGC) vs  lightweight absorbable vs heavyweight mesh | Reoperation for recurrence | Reoperation rate LW 1.7% vs LW-pgc) 2.3% (p 0.001) vs LW-pg 4.1% (p 0.001) vs. HW 1,9% | High quality (++) |

| AUTHOR AND YEAR | STUDY TYPE | POPULATION | COMPARISON | OUTCOME | RESULTS | SIGN CHECKLIST |
| --- | --- | --- | --- | --- | --- | --- |
| Kalra, 2017 | RCT | 60 patients Laparoscopic IHR (TAPP) | Non absorbable vs partially absorbable mesh | Surgical outcomes | Pain (VAS) absorb 2.73±0.944 vs non abs 3.53 ± 0.973 (p 0.03)  Fbs abs 16.7% vs. non abs 36.6% ns  Lump non abs 6.6% vs abs 0 | Acceptable (+) |
| Prakash, 2016 | RCT | 140 patients Laparoscopic IHR (TAPP-TEP) | Lightweight vs heavyweight mesh | CPIP and QoL  Complication recurrence rate | CPIP 3% in both groups (ns)  QOL both groups improves (ns)  No difference in complication and recurrence | Acceptable (+) |
| Roos, 2018 | RCT | 950 patients Laparoscopic IHR (TEP) | Lightweight vs heavyweight mesh | Pain  Fbs  Recurrence (5y)  qol | Recurrence LW 3.8% vs HW1.1% (p 0.01) | High quality (++) |
| Wong, 2017 | RCT | 85 patients Laparoscopic IHR (TEP) | Non absorbable vs partially absorbable mesh | Handling mesh time  LOS  complications | Handling mesh non abs 206 (110-540) vs abs 152(41-360) sec p0.001  Los 0 (ns)  Complications non abs 9/38 vs. abs 5/39 (ns)  Recurrence non abs 0/39 vs. abs 2/38 (ns) | High quality (++) |
| Yang, 2018 | RCT | 102 patients Laparoscopic IHR (TAPP) | Titanium vs PP mesh | Seroma  fbs | Seroma pp 17.3 % vs titanium 6% p0.07  Fbs 32% pp vs 30% titanium (ns) | Acceptable (+) |
| Bakker, 2020 | SR+MA | Laparoscopic IHR (TAPP-TEP)-12 studies- 2909 patients | Lightweight vs heavyweight mesh | CPIP  Recurrence  fbs | Fbs LW 100/1074 vs. HW 103/ 913 (RR 0.94, 95% CI: 0.73–1.20) ns  Pain LW 123/1362 vs. HWM 127/1277 (RR 0.79, 95% CI: 0.52–1.20)  Recurrence LW 32/ 1571 and HWM 13/1508 patients, RR 2.21, 95% CI: 1.14 – 4.31). p 0.02 | High quality (++) |
| Hu, 2019 | SR + MA | Laparoscopic IHR (TAPP-TEP)-12 studies- 3092 patients | Lightweight vs heavyweight mesh | CPIP  Recurrence  Fbs (12 months) | Pain LW did not lower the incidence of chronic pain (RR = 0.93, 95% CI 0.69–1.26, P 0.66)  Recurrence significantly increased in LW (RR=2.28, 95% CI 1.17–4.44, P 0.02)  Fbs no significant difference (RR = 1.00, 95% CI 0.73–1.39, P 0. 99) | Acceptable (+) |
| Wu, 2020 | SR + MA | Laparoscopic IHR (TAPP-TEP)-10 studies- 2518 patients | Lightweight vs heavyweight mesh | Pain  recurrence | Recurrence LW 31/1501 vs HW 12/1575 RR 2.51 (1.33-4.47) p 0.05  CPIP LW 110/729 vs HW 91/713 RR 1.18 (0.91-1.51) p 0.21 | Acceptable (+) |
| Xu, 2019 | SR+MA | Laparoscopic IHR (TAPP-TEP)-11 studies- 1878 patients | Lightweight vs heavyweight mesh | Recurrence pain seroma fbs | Recurrence LW 19/927 vs HW 7/943 RR 2.52 (1.10-5.81) p 0.03  Pain LW 16/712 vs HW 9/723 RR 1.72 (0.77-3.84) p 0.19  Fbs LW 61/490 vs HW 60/477 RR 0.57 (0.10-3.27) p 0.08  Seroma LW 34/353 vs HW 43/364 RR 0.85 (0.56-1.27) p 0.42 | High quality (++) |
| Melkemichel, 2018 | National Registry study | 13839 patients Laparoscopic IHR (TEP) | Lightweight vs heavyweight mesh | recurrence | Recurrence  LW 4.0% (HR 1.56, 95% CI 1.29–1.88; P < 0.001) vs. HW3.2% | High quality (++) |

12. Antibiotic prophylaxis

**Table Quality scores**

| AUTHOR AND YEAR | STUDY TYPE | POPULATION | COMPARISON | OUTCOME | RESULTS | SIGN CHECKLIST |
| --- | --- | --- | --- | --- | --- | --- |
| Orelio 2020 | SR+ metanalysis | 5 RCT- 1865 patients | Suture groin hernia repair in low infection risk environment with vs. without AP | SSI | SSI 1.6% with AP vs. 32.% without AP p= 0.26 | Low (-) |
| Orelio 2020 | SR + metanalysis | 5 RCT- 1865 patients | Suture groin hernia repair in high infection risk environment with vs. without AP | SSI | SSI 8.8% with AP vs. 8.9% without AP p= 0.97 | Low (-) |
| Orelio 2020 | SR+ metanalysis | 27 RCT- 6443 patients | Open mesh groin hernia repair in low infection risk environment wth vs. without AP | SSI | 1.8% with AP vs 2.6% without AP p= 0.16 | Acceptable (+) |
| Orelio 2020 | SR+ metanalysis | 27 RCT- 6443 patients | Open mesh groin hernia repair in high infection risk environment wth vs. without AP | SSI | 4.3% with AP vs 8.5% without AP p= 0.00002 | Low (-) |
| Erdas 2016 | SR+ metanalysis | 16 RCT -5519 patients | Open mesh groin hernia repair in all risk environments | SSI | 3.2% with AP vs. 4.8% without AP (OR 0.68, 95% CI [0.51-0.91] | Low (+) |
| Boonchan 2017 | SR + metanalysis | 15 RCT- 5159 patients | Open groin hernia repair in all risk environments | SSI | Treatment effect for first generation cephalosporins and b-lactam/ b lactamase inibitors (OR 0.62, 95%CI [0.41-0.92] | Acceptable (+) |

13. Anesthesia

**Table Quality scores**

| **Title** | **Authors** | **Type of article** | **population** | **results** | **Quality** |
| --- | --- | --- | --- | --- | --- |
| Network meta-analysis of urinary retention and mortality afterLichtenstein repair of inguinal hernia under local, regional or general anaesthesia | Olsen JHH, Öberg S, Andresen K, Klausen TW, Rosenberg J | Systematic review | 53 studies-11683 patients | higher risk of urinary retention after both regional (odds ratio (OR) 15·73, 95 per cent c.i. 5·85 to 42·32; P < 0·001) and general (OR 4·07, 1·07 to 15·48; P = 0·040) anaesthesia compared with local anaesthesia, and a higher risk after regional compared with general anaesthesia (OR 3·87, 1·10 to 13·60; P = 0·035). Meta-analyses showed a higher risk of urinary retention after regional compared with local anaesthesia (P < 0·001), but no difference between general and local anaesthesia (P = 0·08) | High quality |
| Local VS. other forms of anesthesia for open inguinal hernia repair: A meta-analysis of randomized controlled trials | Argo M, Favela J, Phung T, Huerta S | Systematic review and meta-analysis | 18 rct-n of patients not available | Overall complication rate was similar in LA vs. AO (p = 0.06). Wound infection and hematomas were similar between LA vs. OA, but urinary retention was significantly decreased in LA (p = 0.0002). Patient satisfaction was not inferior with LA (p = 0.10). Surgical time was similar in LA vs. AO (p = 0.86), but operating room time was significantly decreased with LA (p < 0.0001). | High quality |
| Local anaesthesia versus spinal anaesthesia in inguinal hernia repair: A systematic review and meta-analysis | Prakash D, Leonie Heskin L, Doherty S, Galvin R | Systematic review | 10 studies-1379 patients | There was no significant difference in operative time between the groups [Random Effects Model, MD -0.70 min (95% CI, -5.80 to 4.40 min), p = 0.79, I^2^ = 84%]. Patients in the local anaesthetic group experienced significantly less pain than those in the spinal group [Fixed Effects Model, SMD -0.63 (95% CI, -0.81 to -0.46), p < 0.01, I^2^ = 49%], lower rates of urinary retention [FEM, RR 0.03 (95% CI 0.01-0.08), p < 0.01, I^2^ = 0%], decreased rates of anaesthetic failure [FEM, OR 0.17 (95% CI 0.06-0.45), p < 0.01, I^2^ = 0%], and increased satisfaction with the anaesthetic [FEM, OR 3.40 (95% CI 2.09-5.52), p < 0.01, I^2^ = 0%] | High quality |
| Efficacy of local anesthesia in repair of inguinal hernia | Rafiq MK, Sultan B, Malik MA, Khan K, Munir, Abbasi MA | RCT | 60 patients | The day-case rates were significantly higher when patients underwent surgery under LA compared to GA (82.6% versus 42.6%). The incidence of urinary retention was higher in the GA group (p<0.05). There were 17 (2.9%) re-admissions overall. The reasons for readmission included haematoma (n=6), severe pain (n=4), infection (n=3), fainting (n=2) and urinary retention (n=2). | Acceptable |
| Randomized comparison of the feasibility of three anesthetic techniques for day-case open inguinal hernia repair | Pere P, Harju J, Kairaluoma P, Remes V, Turunen P, Rosenberg PH | RCT | 156 patients | Surgery lasted longer in Lidocaine (LAI) group (median 40min) than in bupivacaine (SPIN)group (35min) (P=.003) and propofol (TIVa) group (33min) (P<.001). Although surgery was shortest in TIVA group, TIVA patients stayed longer in the operating room than LAI patients (P=.001). Time until readiness for discharge was shorter in LAI group (93min) than in TIVA (147min) and SPIN (190min) groups (P<.001). | High quality |
| A Comparison between Local and Spinal Anesthesia in Inguinal hernia Repair | Zamani-Ranani MS, Moghaddam NG, Firouzian A, Fazli M, Hashemi SA | RCT | 60 patients | The pain score at the 3, 6 and 12 hour periods after surgery was significantly lower in LA group (P<0.0001). But there was no significant difference between groups in pain score at the 24 hour period after surgery (P=0.24). Also, the LA group needed lower analgesic agents (P=0.001). | High quality |
| Tolerability and Outcome of Inguinal Hernia Repair under Local Anesthesia among elderly male patients high risk for General or Regional Anesthesia | Faisal MS, Farid MN, Mahmood M | Prospective study | 100 patients | Local anesthesia was tolerable in 95(95%) patients. Pain during procedure was seen in 3(3%) patients, while nausea/vomiting and headache in 1(1%) patient each. Postoperatively, pain was seen among 4(4%) patients, urinary retention in 1(1%), inguinodynia in 1(1%) patient, and scrotal hematoma in 2(2%) patients. | High quality |
|  |  |  |  |  |  |

19. CPIP

**Table Quality scores**

| **reference** | **patients** | **objective** | **results** | **quality** |
| --- | --- | --- | --- | --- |
| Wijayasinghe N, 2016, br j anesth- RCT | 14 | investigate the analgesic effects of local anaesthetic TP-blockade. | The median (95% CI) reduction in pain was 63% (44.1 to 73.6%) after bupivacaine compared with 36% (11.6 to 49.7%; P=0.003) after placebo. Significant increases in cool detection (P=0.01) and pressure pain thresholds (P=0.009) with decreases in supra-threshold heat pain perception (P=0.003) were seen after bupivacaine only | High (++) |
| Trainor, 2015 J Pain Res- observational | 36 | to compare the effectiveness of landmark-based and ultrasound-guided ilioinguinal/iliohypogastric nerve blocks in the treatment of CPGP. | The average VAS score preinjection was 7.08 in the landmark-based and 7.0 in the ultrasound-guided groups (P=0.65). A total of 14 patients (70%) in the landmark-based and eleven patients (79%) in the ultrasound-guided groups experienced at least a 50% reduction in VAS scores. There was no statistically significant difference between the two groups (P=1.0), and no complications were noted. | Acceptable (+) |
| burgmans, hernia, 2016- observational | 53 | in the present study the yield of MRI in evaluating chronic pain after TEP hernia repair is addressed | Imaging studies of 53 patients revealed information regarding 106 groins. None of the predefined disorders was observed statistically more often in the patients with painful groins. Only fibrosis appeared more prevalent in patients with chronic pain (P = 0.11). In 15 % of the patients, MRI revealed treatable findings explanatory for persisting groin pain. | Acceptable (+) |
| Verhagen T, 2018, ann surg- RCT | 54 | This study compares tender point infiltration (TPI) and a tailored neurectomy as the preferred treatment for chronic inguinodynia after inguinal herniorraphy. | Baseline VAS was similar (TPI: 55, range 10-98 vs neurectomy: 53, range 18-82, P = 0.86). TPI was successful in 22% (n = 6), but a neurectomy was successful in 71% (n = 17, P = 0.001). After unsuccessful TPI, 19 patients crossed over to neurectomy and their median VAS score dropped from 60 to 14 (P = 0.001). | Acceptable (+) |
| Valvekens E,2015, hernia, observational | 15 | This retrospective study reports the outcome and investigates patient and intra-operative factors to identify possible predictors of success | Overall, significant pain reduction was achieved in 1/3 of patients. There was no significant association between patient or intra-operative factors and favorable outcome. A complete concordance between subjective outcome and the ratio of VASmax (post/pre) was noted | Acceptable (+) |
| Magnusson N, hernia, 2015- registry | 237 | The aim of the present study was to assess the outcome results after reoperation for persistent pain after hernia surgery in a population-based setting. | Decrease in pain after the most recent reoperation was reported by 69 patients (62%), no change in pain by 21 patients (19%) and increase in pain in 21 patients (19%). There was no significant difference in outcome between mesh removal, removal of sutures at the tubercle or interventions aimed at the ilioinguinal nerve. All subscales of SF-36 were significantly reduced when compared to the age- and gender-matched general population (p < 0.05). | Acceptable (+) |
| Sun P, 2016, Am j surg- observational | 44 | reoperation for treatment of CPIP (R4GP) | 26 (93%) respondents indicated they experienced pain after their last R4GP for a median duration of 12.5 months. At study completion, 5 patients continued to have debilitating chronic groin pain, 5 had moderate pain, 6 had minimal discomfort, and 12 were pain-free. Twenty-four respondents (86%) would proceed with reoperation(s) again if they could go back in time. | Acceptable (+) |
| Moore AM, 2016, am j surg- observational | 62 | This prospective study evaluated long-term outcomes associated with laparoscopic retroperitoneal triple neurectomy. | Mean numerical pain scores were significantly decreased (baseline, 8.6) at all postoperative time points (POD 1, 3.6; P < .001: POD 90, 2.3, P < .001) with durable efficacy from POD 90 to 3 years (P < .001). | Acceptable (+) |
| Bjurström MF, 2017, pain practice- observational | 10 | results of laparosocpic retroperitoneal triple neurectomy | Quantitative Sensory Testing revealed marked increases in mechanical, pressure, thermal, and pain thresholds in the areas with maximum pain prior to LRTN surgery for the immediate (P < 0.01; mean 160.9 minutes, range 103 to 255 minutes after extubation) and late postoperative (P < 0.05; mean 27.9 days, range 14 to 78 days after surgery) assessments compared to baseline. | Acceptable (+) |
| Moreno-Egea A, 2016, surg end- observational | 16 | The objective of this study is to evaluate the selective transabdominal preperitoneal laparoscopic neurectomy for treatment of refractory inguinodynia. | One patient developed hypoesthesia in the territory of the femorocutaneous nerve by nerve injury. Reoperation was performed 6 months afterward to complete ilioinguinal nerve neurectomy. Neuropathic pain medications were continued by five patients. Pain was completely eliminated in 11 (68.75 %). | Acceptable (+) |
| Slooter GD, 2018, surg end- observational | 14 | effects of laparoscopic mesh removal for the treatment of CPIP | Eight months postoperatively (median), pain scores had dropped from eight to four (p < 0.01). Satisfaction was good or excellent in ten patients. A recurrent hernia developed in two patients requiring an open mesh repair in one. | Acceptable (+) |
| Karampinis I, 2017, bmc surg- observational | 9 | analysis of results of a minimal-invasive approach to neurectomy on quality of life and pain relief. | Four patients were free of pain after neurectomy, three described an improved pain status, whereas two did not observe any change in pain. Within a follow-up period of 14,3 months, no deterioration of pain or other complications were observed. Patients who underwent neurectomy had significantly lower quality of life compared to the control group. | Acceptable (+) |
| Pedersen KF, 2021, scand surg- observational | 240 | to analyze pain-related functional impairment using a simplified clinical treatment algorithm for a standardized surgical treatment | Follow-up was a median 3 months (range: 3-13). Activity Assessment Scale scores were clinically relevant improved in 43 patients (68%), not clinically relevant different in 19 (30%), and clinically relevant worsened in one (2%). Secondary outcome scores were all significantly improved (*P* < 0.05) except for the risk of postoperative depression (*P* = 0.092). Fifty-one patients (77%) reported that chronic groin pain was reduced after the operation. | Acceptable (+) |
| Gangopadhyay N, 2020, ann pl surg- observational | 12 | retroperitoneal approach to the ipsilateral ilioinguinal, iliohypogastric, and genitofemoral nerves with neurectomy and proximal transposition of these nerves-for management of neuropathic pain | Pain visual analog scores (VASs) demonstrated significant improvement after neurectomy (preoperative pain VAS of 85 ± 11 vs postoperative pain VAS of 47 ± 32, P = 0.0027). | Acceptable (+) |
| Ramshaw B, 2017, surg end- observational | 68 | results of laparoscopic approach to CPIP | Forty-five patients (48%) reported significant improvement, 39 patients (41%) reported moderate improvement, and 10 patients (11%) reported little or no improvement | Acceptable (+) |
| Zwaans WA, 2017, WJS- observational | 74 | define mesh-related pain symptoms, to investigate long-term effects of a meshectomy and to provide recommendations on meshectomy. | A patient with a pure mesh-related groin pain characteristically reports a 'foreign body feeling'. Pain intensifies during hip flexion (car driving) and is attenuated following hip extension or supine position. Palpation is painful along the inguinal ligament whereas neuropathic characteristics (hyperpathic skin, trigger points) are lacking. | Acceptable (+) |
| Zwaans WA, 2015, WJS- observational | 136 | to identify potential patient- or surgery-related factors predicting the surgical efficacy for inguinodynia following Lichtenstein repair. | Factors contributing to success were removal of a meshoma (OR 4.66) or a neuroma (OR 5.60) and the use of spinal anaesthesia (OR 4.38). In contrast, female gender (OR 0.30) and preoperative opioid use (OR 0.38) were significantly associated with a less favourable outcome. Using a multivariate analysis model, surgery under spinal anaesthesia (OR 4.04), preoperative use of opioids (OR 0.37), and meshoma removal (OR 5.31) greatly determined surgical outcome. | Acceptable (+) |
| Marconi M,2015, j urol- observational | 50 | prospectively evaluation of results of microsurgical spermatic cord denervation in a series of patients with chronic scrotal content pain in a multicenter study | Six months after surgery 40 patients (80%) were completely pain-free. In 6 patients (12%) intermittent testicular discomfort persisted, which could be managed by acetaminophen on demand. Four patients (8%) had no change in pain severity after surgery. | Acceptable (+) |
| calixte N, 2018, j urol- observational | 772 | to present the outcomes of ligation of these nerves using a technique of targeted robotic assisted microsurgical denervation of the spermatic cord. | During a median followup of 24 months (range 1 to 70) 718 cases (83%) showed a significant reduction in pain and 142 (17%) had no change in pain by subjective visual analog scale scoring. | Acceptable (+) |
| Poh F, 2019, Am j Roent- observational | 106 | the role of MR neurography (MRN) in the evaluation of border nerve abnormalities and the results of treatments directed at the MRN-detected nerve abnormalities. | mprovement in subjective pain was seen in 53 of 63 cases (84.2%). A statistically significant improvement in pain response was noted in the isolated ilioinguinal nerve block group as compared with the isolated genitofemoral nerve block group (p = 0.0085) | Acceptable (+) |
| Lee KS,2019, j vasc & int radiol- observational | 12 | To evaluate the feasibility and efficacy of ultrasound-guided microwave ablation for the treatment of inguinal neuralgia. | Improved pain levels immediately after the procedure and at 1, 6, and 12 months were statistically significant (P = .0037, .0037, .0038, .0058, respectively). Also, 91.7% (11/12) of the procedures resulted in immediate pain relief and at 1 month and 6 months | Acceptable (+) |
| Shaw A, 2016, br j neurosurg- observational | 6 | results of peripheral nerve stimulation has become an effective and minimally invasive option for the treatment of refractory pain. | All six patients had an average improvement of 62% in the immediate post-operative follow-up. | Acceptable (+) |
| Calixte N,2019, urology- observational | 279 | To assesses the efficacy of ultrasound-guided targeted cryoablation (UTC) of the perispermatic cord as a salvage treatment for patients who failed microsurgical denervation of the spermatic cord | Subjective visual analog scale outcomes: 75% significant reduction in ain (11% complete resolution and 64% ≥50% reduction in pain). Objective Pain Index Questionnaire-6 outcomes: 53% significant reduction at 1 month (279 cases), 55% at 3 month (279 cases), 60% at 6 month (279 cases), 63% at 1 year (279 cases), 65% at 2 years (275 cases), 64% at 3 years (232 cases), 59% at 4 years (128 cases) and 64% at 5 years (53 cases) post-op. | Acceptable (+) |
| Calixte N, 2017, curr urol report- review | na | to review and discuss the management of patients with chronic orchialgia using currently available literature | - | Acceptable (+) |
| Landry M, 2020, surg end- observational | 129 | application of a prehabilitation program including Cognitive Behavioral Therapy (CBT), aim to improve the surgical outcomes of surgical approach to CPIP | Overall, 15 (14.7%) patients had no improvement in symptoms after surgery from the non-CBT group, whereas there was improvement in chronic pain for all patients who underwent CBT. | Acceptable (+) |

21. Emergency

**Table Quality scores**

| **Author** | **Journal** | **Title** | **Study Type** | **results** | **Quality of studies** |
| --- | --- | --- | --- | --- | --- |
| Chen P, 2019 | American Journal of Emergency Medicine | Risk factors for bowel resection among patients with incarcerated hernias: A meta-analysis | meta-analysis  7 studies-762 patients | Risk for bowel resection· 8 risk factors for bowel resection · Helps to establish which patient will need acute surgery and will have poorer outcomes. | Acceptable(+) |
| Azari Y, 2015 | Hernia | Strangulated groin hernia in octogenarians. | retrospective cohort- 200 patients | Emergency surgery for strangulated hernia repair in patients over 80 years is more complicated than in younger patients, mostly due to the existing comorbidities | Acceptable (+) |
| Reinke CE, 2020 | J Gastrointest Surg. | What's New in the Management of Incarcerated Hernia | narrative review | Useful for definition for acutely irreducible hernia | Low (-) |
| Bessa SS, 2015 | Hernia | Results of prosthetic mesh repair in the emergency management of the acutely incarcerated and/or strangulated groin hernias: a 10-year study. | prospective cohort- 234 patients | Use of a mesh in surgeries with bowel necrosis is Acceptable. | Acceptable (+) |
| Köksal H, 2018 | Ulus Travma Acil Cerrahi Derg | Predictive value of preoperative neutrophil-to-lymphocyte ratio while detecting bowel resection in hernia with intestinal incarceration | retrospective cohort- 102 patients | Use of biomarkers to distinguish viability of bowel. | Low (-) |
| Duan SJ, 2018 | Am Surg. | Prosthetic Mesh Repair in the Emergency Management of Acutely Strangulated Groin Hernias with Grade I Bowel Necrosis: A Rational Choice. | retrospective cohort- 208 patients | Grading of surgeries according to intra-op findings. Use of mesh based on grades | Acceptable (+) |
| Zhao F., 2019 | Minerv. Chir. | Clinical effects of prosthetic mesh in the treatment of incarcerated groin hernias. | retrospective cohort- 219 patients | Use of definition | Acceptable (+) |
| Xie X., 2017 | Med Sci Monit | Neutrophil-to-Lymphocyte Ratio Predicts the Severity of Incarcerated Groin Hernia. | retrospective cohort - 95 patients | Acute hernia definition. Neutrophil to leucocyte ratio. – biomarkers | Acceptable (+) |
| Tebala GD, 2019 | Hernia | Hernioscopy: a reliable method to explore the abdominal cavity in incarcerated or strangulated inguinal hernias spontaneously reduced after general anaesthesia. | retrospective series- 8 patients | laparoscopy through hernial sack. 3/8 influence outcome. | Low (-) |
| Duan SJ, 2015 | int Surg | A novel bowel necrosis classification system and examination of patient outcomes in incarcerated groin hernia patients. | retrospective cohort- 108 patients | Grading of surgeries according to intra-op findings. Use of mesh based on grades | Acceptable (+) |
| Chen P, 2020 | Medicine | Analysis of risk factors associated bowel resection in patients with incarcerated groin hernia. | retrospective cohort- 323 patients | Risk factors on bowel ischemia and need for bowel resection | Acceptable (+) |
| Chen F., 2020 | Surg Innov. | Tension-Free Mesh Repair for Incarcerated Groin Hernia: A Comparative Study. | retrospective cohort- 118 patients | suture had worse outcomes | Acceptable (+) |
| Ceresoli M, 2020 | Hernia | Emergency hernia repair in the elderly: multivariate analysis of morbidity and mortality from an Italian registry. | prospective registry- 259 patients | major complications and risk of mortality directly link to midline laparotomy. | Acceptable (+) |
| Kohga A,2020 | ANZ J Surg | Emergency surgery versus elective surgery after reduction for patients with incarcerated groin hernias | prospective cohort- 76 patients | Manual reduction safe option, Time from manual reduction to surgery | Acceptable (+) |
| Chung PJ, 2005 | Hernia | Predicting 30-day postoperative mortality for emergent anterior abdominal wall hernia repairs using the American College of Surgeons National Surgical Quality Improvement Program database. | retrospective database review- 1046 patients | Risk factors for surgical outcome | Acceptable (+) |
| Dai W., 2005 | Hernia | Risk factors of postoperative complications after emergency repair of incarcerated groin hernia for adult patients: a retrospective cohort study. | retrospective cohort- 64 patients | Mesh repair for incarcerated groin hernia is associated with decreased recurrence rate compared with non-mesh repair | Acceptable (+) |
| Liu J., 2020 | Surg Endo. | The results of open preperitoneal prosthetic mesh repair for acutely incarcerated or strangulated inguinal hernia: a retrospective study of 146 cases. | retrospective cohort- 146 patients | No statistically significant difference between two subgroups of patients with viable content or ischemic bowel (and bowel resection) who underwent mesh repair, as long as a contaminated surgical field is not present. | Acceptable (+) |
| Chihara N, 2019 | Laparoendosc Adv Surg Tech | Is the Laparoscopic Approach Feasible for Reduction and Herniorrhaphy in Cases of Acutely Incarcerated/Strangulated Groin and Obturator Hernia?: 17-Year Experience from Open to Laparoscopic Approach | prospective non-randomized compare- 106 patients | The prospective non-randomized study by Chihara *et al.* comparatively analyzed the surgical outcomes of 106 patients who underwent open (50.9%) and laparoscopic repair (49.1%) for acutely incarcerated/strangulated groin and obturator hernias. | High (++) |
| East B, 2020 | Hernia | A manual reduction of hernia under analgesia/sedation (Taxis) in the acute inguinal hernia: a useful technique in COVID-19 times to reduce the need for emergency surgery-a literature review. | systematic review- n of patients na | Taxis can be successful in up to 70%; The is a linear relationship between the time from the onset of symptoms to strangulation | Acceptable (+) |
| B. East et al.2020 | Rozhledy v chirurgii | Recommendations for patient management after manual reduction of incarcerated inguinal hernia: a literature review | descriptive review- n of patients na | A review published by East et al. has also reported on serum levels of D-dimer above 300 ng/ml and serum phosphokinase levels of 140 IU/l and higher (compared to 90 in the control group) together with signs of bowel obstruction as good predictors of bowel ischaemia with relatively low specificity but both sensitivity and negative predictive values over 90% | Low (-) |
| Birindelli, A, 2017 | World J Emerg Surg | 2017 update of the WSES guidelines for emergency repair of complicated abdominal wall hernias. | Guidelines | In case of strangulated hernia surgery ASAP is indicated (within 2h) | - |
| Köckerling F, 2021 | Front Surg. | Trends in Emergent Groin Hernia Repair-An Analysis From the Herniamed Registry | Large cohort- 13028 patients | Atemt taxis if no strangulation, laparoscopi aproach | High (++) |
| Pawlak M,2021 | Hernia | Algorithm for management of an incarcerated inguinal hernia in the emergency settings with manual reduction. Taxis, the technique and its safety. | narrative review | Save aproach oward manual reduction | Low (-) |
| Liu, J, 2021 | World J Emerg Surg | If laparoscopic technique can be used for treatment of acutely incarcerated/strangulated inguinal hernia? | retrospective cohort- 94 patients | Safety and feasibility of TAPP for treatment of patients with acutely incarcerated/strangulated inguinal hernias | High (++) |

28. Non-commercial mesh

**Table Quality scores**

| reference | Type of publication | content | conclusion | Sign checklist |
| --- | --- | --- | --- | --- |
| Patterson et al  *A systematic review and meta-analysis of the post-operative adverse effects associated with mosquito net mesh in comparison to commercial hernia mesh for inguinal hernia repair in low income countries.* | Systematic review-5 studies- 628 patientss | Comparison mesh versus low-cost mesh | No sign difference after one year follow-up | High quality (++) |
| Ahmad  *Meta-analysis of the use of sterilized mosquito net mesh for inguinal hernia repair in less economically developed countries* | Meta-analysis- 9 studies- 1085 patients | Comparison mesh versus low-cost mesh | No sign difference after one year follow-up | Acceptable (+) |
| Lofgren-  *Cost-effectiveness of groin hernia repair from a randomized clinical trial comparing commercial versus low-cost mesh in a low-income country.* | RCT 302 patients | Cost analysis | low-cost mesh cost effective | High quality (++) |
| Mitura-  *The influence of different sterilization types on mosquito net mesh characteristics in groin hernia repair* | Review- n patients na | Chemical and biomechanical properties after sterilisation | Quality of the different types of non-commercial mesh especially after sterilization remains a concern. | Acceptable (+) |
